# Drift-diffusion dynamics of hippocampal replay

**DOI:** 10.1371/journal.pcbi.1014761

**Published:** 2026-09-22

**Authors:** Zhongxuan Wu, Xue-Xin Wei

**Affiliations:** 1 Department of Neuroscience, The University of Texas at Austin, Austin, Texas, United States of America; 2 Center for Perceptual Systems, The University of Texas at Austin, Austin, Texas, United States of America; 3 Center for Learning and Memory, The University of Texas at Austin, Austin, Texas, United States of America; 4 Center for Theoretical and Computational Neuroscience, The University of Texas at Austin, Austin, Texas, United States of America; 5 Department of Psychology, The University of Texas at Austin, Austin, Texas, United States of America; The University of Edinburgh, UNITED KINGDOM OF GREAT BRITAIN AND NORTHERN IRELAND

## Abstract

Replay in the hippocampus during sharp-wave ripples is thought to play major roles in learning and memory. However, existing analysis methods often lead to inconsistent and inaccurate metrics for characterizing replay dynamics. We develop a novel computational framework that models the replay dynamics using a drift-diffusion process. Further, to capture the potentially rich population-level dynamics during sharp-wave ripples, our model allows switching between multiple well-motivated types of dynamics. Applications of our method to rat hippocampal recordings lead to a number of insights. First, our results reveal that a small fraction of SWRs are stationary, and those with drift-diffusion dynamics generally have a much higher speed than the animal’s movement. Second, we find that replay dynamics are heterogeneous and inconsistent with a random walk model proposed previously. The mean-squared displacement of replay trajectories scales quadratically with time, supporting the presence of substantial drifts. Third, we find that only a tiny fraction of SWR events (less than 1%) exhibit sequential structure at the timescale of 100 ms before the animal had spatial experience in an environment. This suggests that, while neural activity in the hippocampus may be coordinated before the animal’s spatial experience (i.e., preplay), the level of coordination is much weaker than that after spatial experience. Overall, our approach enables precise characterizations and unambiguous interpretations of population dynamics during sharp-wave ripples, which more broadly can provide a better understanding of the functions and underlying mechanisms of replay.

## Author summary

During sharp-wave ripples (SWRs), neural populations in the mammalian hippocampus sometimes replay the neural activity patterns reminiscent of previous experience. Such replay events have been implicated in various cognitive functions, such as learning, memory and planning. Despite extensive prior research, the field still lacks appropriate inferential methods to accurately characterize the potentially rich neural dynamics during SWRs. This issue has led to substantial limitations in interpreting the experimental data collected in previous studies. To address this gap, we have developed a computational modeling framework to infer and quantify the neural dynamics during SWRs. This approach is flexible in that it captures different types of neural dynamics as well as the switching of neural dynamics in a unified model. Meanwhile, the parameters in the model are highly interpretable. Applications of this method to neural population recordings from a one-dimensional spatial task in rats reveal new insights into a number of questions that are currently under debate regarding the structure and property of hippocampal replay.

## 1 Introduction

Neural activities in the mammalian hippocampus during sharp-wave ripples (SWRs) have been found to “replay” previous experience in forward [1,2] or reverse [3,4] order, both during sleep [5–8] and awake immobility [3,9]. Replays in the hippocampus are also found to be coordinated with neural activities in other brain regions, e.g., prefrontal cortex [10–12], visual cortex [13], and brain-wide spontaneous dynamics [14]. While initially reported in rodents, experimental evidence for replay in the human brain has also been reported in recent studies [15–19]. As a form of selective and experience-dependent neural activity, replays have been proposed to play an important role in learning, memory consolidation, and planning [20–23], although their precise functions remain to be determined.

Surprisingly, even given decades of research on hippocampal replay, the methodology of detecting replays and, at the same time, identifying their properties on an event-by-event basis remains an ongoing challenge, both statistically and also with respect to defining the phenomenon [20,24]. Most studies rely on template matching, where putative replay activity is compared to a template generated from the population activity during “online” periods, typically taken to be running [5,6]. More specifically, this template can be defined using either a sequential order of neurons or the sequential structure in a decoded variable, conventionally spatial location. However, many SWR events remain ambiguously classified according to this approach, posing a challenge for subsequent analyses and, further, suggesting that many replays may have structure that is not well-captured by existing approaches. As an example, in prior methods, the speed and quality (coherence) of replays are entangled. Replay events with high speed tend to be significant despite having lower coherence, even though these variables are ideally independently estimated. Indeed, recent studies suggest that replays may exhibit richer dynamics than traditionally considered or assumed [25,26], and the properties of replay dynamics may have major implications for learning [27]. In this way, the lack of a principled and appropriately general computational framework for characterizing the dynamics of replay constitutes a major obstacle to analyzing and interpreting ongoing experimental data and findings, respectively.

Here we address this gap by developing a principled probabilistic approach for unbiased characterization of the dynamics of replay. This method builds upon prior work [25,26,28–30] and overcomes multiple limitations in accurate identification of replay dynamics. We will show that applications of our modeling framework to neural population recordings from the rodent hippocampus lead to new insights into the precise neural dynamics of hippocampal replay. First, in contrast to recent observations in [25], our results show that only a small fraction of SWRs (~ 5%) are stationary, and furthermore, those with drift-diffusion dynamics generally have a much higher speed than the animal’s movement. Second, we find that replay dynamics in 1-D environments are heterogeneous, and are inconsistent with a random walk model proposed previously [31]. The mean squared displacement of replay trajectories scales quadratically with time, supporting the presence of substantial drifts. Third, we find that only a tiny fraction of SWR events (less than 1%) exhibit sequential structure at the timescale of 100 ms before the animal had spatial experience in an environment. This suggests that, while neural activity in the hippocampus may be coordinated before the animal’s spatial experience (i.e., preplay [32]), the level of coordination is much weaker than that after spatial experience. By enabling more flexible and interpretable characterization of replays, this new analysis framework may provide a path toward better understanding of the functions and mechanisms of replay.

## 2 Results

A replay event is generally thought to be a sequence of neural activity that encodes a traversal of a particular state space (e.g., positions on the track). Most previous studies use a two-step procedure to analyze replays: (i) decode spatial location from neural population activity, typically using a Bayesian decoder that yields decoded posterior probabilities; (ii) apply a “manually” specified form of template matching to the decoded posterior probabilities to detect putative sequential structure that would qualify as a (traditional) replay. Despite the success of this approach in identifying classical replays, the major limitation is the rigidity of the pre-specified template, which effectively disregards the potential richness of replay dynamics. A second major limitation is that the estimation in the second step fails to incorporate uncertainty, which limits quantitative evaluation of robustness. To address these issues, we develop a novel set of state-space models based on drift-diffusion dynamics to characterize the structure of replay events. By basing dynamics on drift-diffusion, a general yet interpretable and parsimonious process, our approach rigorously formalizes past intuition regarding replays, and moreover allows for direct probabilistic inference of replay dynamics from neural data.

### 2.1 Drift-diffusion models of replay dynamics

**Fig 1:**
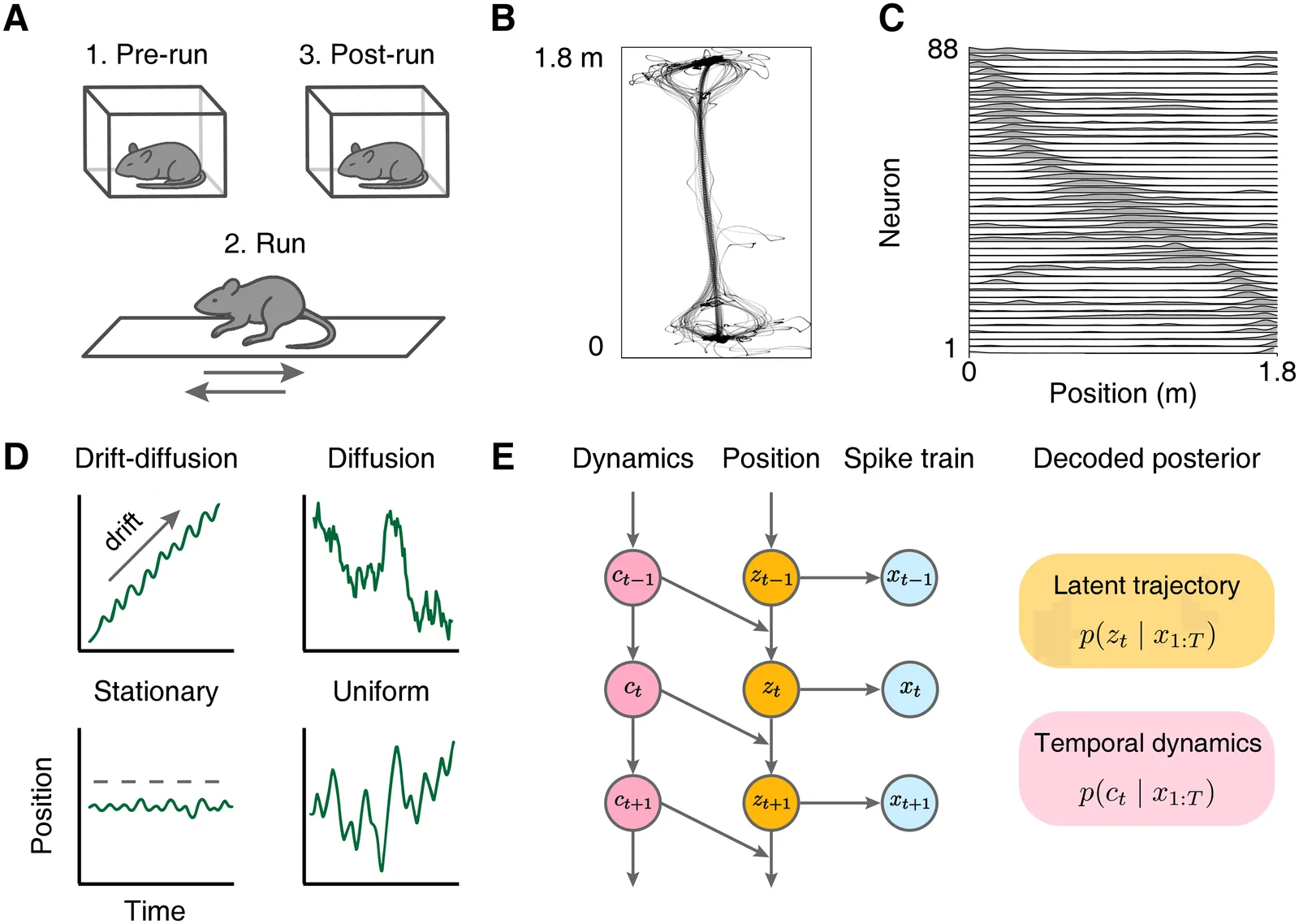
Behavioral paradigm and the overall framework for characterizing hippocampal replay. **A**. Schematic of the behavioral paradigm to study hippocampal replay. Each recording session consisted of three behavioral epochs: pre-run sleep in an isolated box, active running on a 1-D linear track, and post-run sleep in the same box. **B**. Head-position tracking during the run epoch from an example recording session. The animal performed repeated back-and-forth traversals on a 1.8 m track. The track was 6.35 cm wide, with reward wells at each end that expanded to 12.7 cm. Data are replotted from [33]. **C**. Place fields of recorded CA1 neurons estimated from neural activity during running (> 5 cm/s). For clarity, only half of the recorded population is shown. Data are replotted from [33]. **D**. Candidate spatiotemporal dynamics (regimes) for latent trajectories during sharp-wave ripples (SWRs). *Drift-diffusion* combines deterministic drift with stochastic diffusion, dz=λdt+σdW, where λ controls speed and σ controls Brownian motion amplitude. *Diffusion* is pure Brownian motion, dz=σdW, corresponding to an unbiased random walk. *Stationary* dynamics, *dz* = 0, constrain the latent position to remain fixed over time. *Uniform* is temporally discontinuous dynamics in which position is independently resampled from a uniform distribution at each time bin, approximating a drift-diffusion process with near-zero drift and large diffusion. **E**. Left, probabilistic graphical model of the switching hidden Markov model. At each time bin, the discrete regime variable $c_{t}$ selects a transition kernel for latent position $z_{t}$, which generates observed spike counts $x_{t}$ through Poisson emissions parameterized by the estimated place fields. Right, probabilistic inference yields both the marginal posterior over latent trajectory, $p(z_{t}|x_{1:T})$, and the marginal posterior over regimes, $p(c_{t}|x_{1:T})$.

Consider an experiment where the animal is running on a one-dimensional track (Fig 1A and 1B), a common behavioral paradigm for studying replays. Although we focus on modeling replays in a 1-D environment, our model can be generalized to more complex environments. We assume that hippocampal activity during a replay event encodes a sequence of positions on the track (Fig 1C). However, in contrast to previous work, we propose to model changes in encoded positions during replays as evolving according to the dynamics specified by drift-diffusion. Formally, these dynamics of replayed positions *z* during putative replays (population activity during SWRs) evolve according to (Fig 1D):


$$\begin{array}{c} dz=\lambda dt+\sigma dW \end{array}$$ (1)

where λ and σ are the drift and diffusion parameters, respectively. dW denotes the standard Brownian increment. This model can be implemented in discrete time steps, with the dynamics described by the state-transition matrix. We further assume that, given a latent state, the activity of individual neurons follows an independent Poisson process with firing rate dependent on location-specific fields (place fields) –an assumption shared with the conventional Bayesian decoding method for analyzing replays (see Methods).

In this framework, the expected latent state at the next time step is determined by the current state and the drift rate, with the variability of the states determined by the diffusion parameter. In this way, both parameters (drift and diffusion) are highly interpretable. The drift parameter is analogous to the speed (or “virtual speed”) of the replay event. The diffusion parameter quantifies how noisy the replay trajectory is. Different values of the two parameters can capture a variety of dynamical patterns while retaining interpretability (Fig 1D). In the case of zero drift and nonzero diffusion, the latent trajectory is equivalent to one-dimensional Brownian motion, a pattern recently proposed to capture replay dynamics [31]. In the case where both drift and diffusion parameters are near zero, the trajectory is largely stationary, a dynamical pattern emphasized in [25]. Finally, in the case where the drift rate is nearly zero, and diffusion is large, the discretized trajectory would behave similarly to a collection of independently sampled states having little coherence. We refer to this type of event as a uniform event. Conceptually, this is the opposite regime of replay events discussed in previous studies.

**Fig 2:**
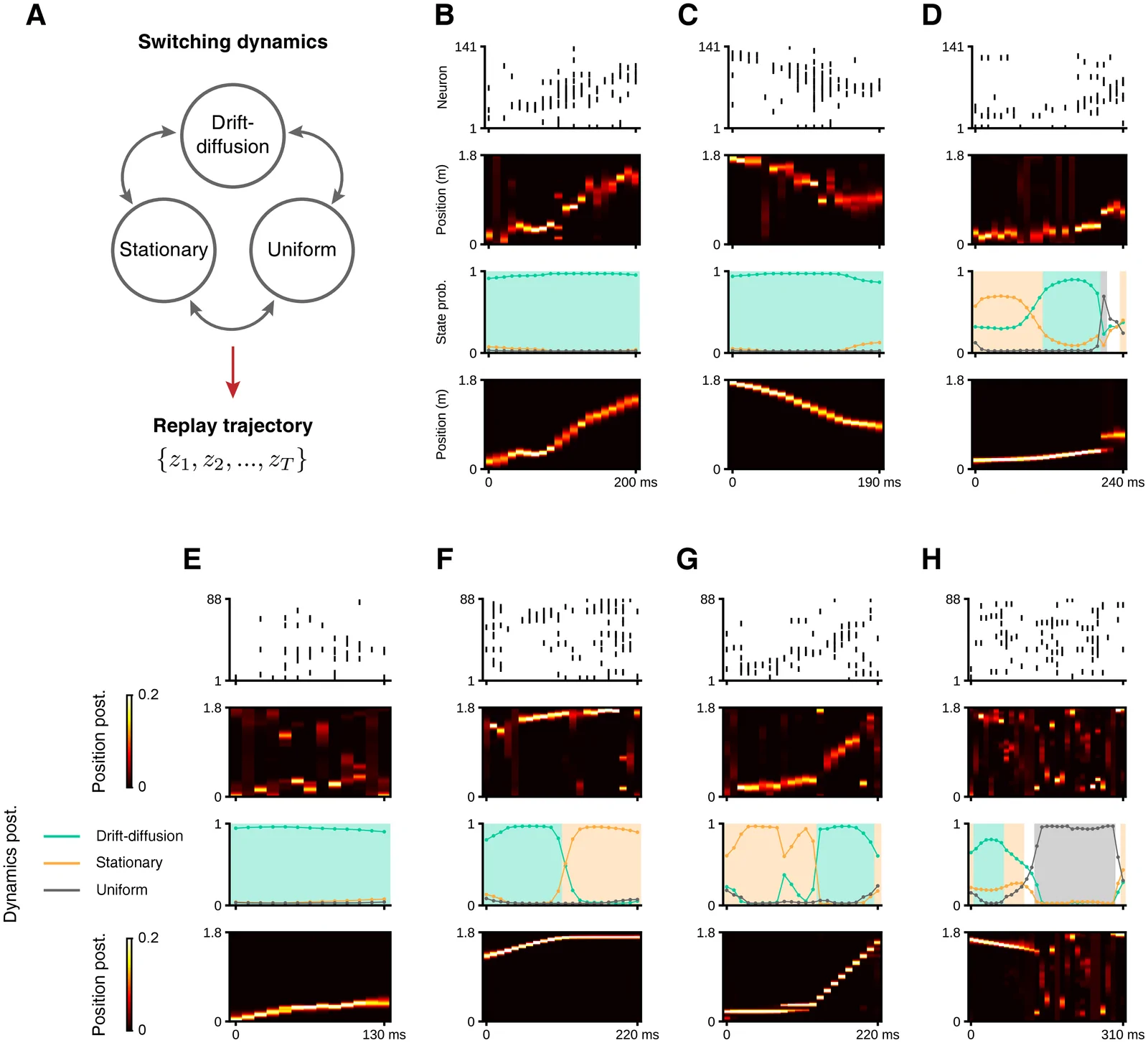
Decoding heterogeneous dynamics in rat hippocampal sharp-wave ripples with a switching hidden Markov model. **A**. Schematic of the switching-dynamics framework. At each time step, the latent trajectory can evolve according to one of three transition regimes: drift-diffusion, stationary, or uniform dynamics (defined in Fig 1D). The inferred sequence of regimes determines the transition probabilities between successive latent positions, $p(z_{t}|z_{t-1})$, and thereby the trajectory of SWRs $\left\{z_{1},z_{2},...,z_{T}\right\}$. **B–H**. Seven representative SWRs from a rat hippocampal recording dataset [33]. Each example is shown in four rows. From top to bottom: spike raster for the SWR, with neurons ordered by place-field peak location and spikes binned in 10 ms non-overlapping bins; position posterior decoded with a memory-less Bayesian decoder [34]; posterior probability of the three dynamic regimes inferred by the switching HMM, $p(c_{t}|x_{1:T})$; and position posterior inferred by the switching HMM, $p(z_{t}|x_{1:T})$. Background shading in the dynamics-posterior row denotes the assigned regime at each time bin. A time bin is assigned to a regime when that regime has posterior probability greater than 0.7. The rest of the time bins are assigned to stationary when the summed drift-diffusion and stationary posterior probabilities exceed 0.7. This combination corresponds to a reduction of drift-diffusion to stationary dynamics. Remaining bins are labeled as unclassified (No Class). Dynamics color code: drift-diffusion, green; stationary, orange; uniform, gray; no class, none.

**Extended modeling framework to incorporate switching dynamics.** A fundamental matter that remains unresolved regarding replays is whether and to what extent individual SWR events express multiple dynamical patterns serially [25]. To capture the potential richness of the dynamics of an SWR event, we incorporate the switch between different types of dynamics within an individual event (Fig 1E). Importantly, both the type of dynamics and the states (i.e., positions) can be inferred from the neural activity based on our model. The resulting one-step framework represents a powerful way to clarify whether and to what extent there are mixtures of dynamics. Specifically, we propose that an SWR event may be serially comprised of three types of dynamics (regimes): drift-diffusion, stationary, and uniform sampling (Fig 2A). For a given event, in addition to the latent position evolution, there is also a finite probability of switching to a different type of dynamics. We assume that the transition probability matrix between regimes has equal self-transition probability *q* and identical switching probabilities $\frac{1-q}{2}$ of switching to the other two regimes. The parameter *q* is optimized for individual SWR events and thus can capture variability of dynamical switching which may be expressed across different events, as suggested in previous work [35]. The transition matrix between different regimes can be easily incorporated into the model by conditioning the state transition matrix for each type of dynamics. We develop inference procedures for estimating the parameters from the neural population activity, leveraging established techniques in HMMs and state-space models [36–43]. For each SWR event, we optimize the model parameters {λ,σ,q} by maximizing the likelihood of observing the neural population activity. With optimized parameters, we can calculate the posterior distribution of dynamics and positions (see Methods and S6 Text for details). Fig 2B–2H shows example events from analyzing a hippocampal population recording dataset [33].

### 2.2 Validation of the modeling framework

Our full model of replay dynamics can be interpreted as a specific type of switching HMM. Although identifiability has been established for related models [44,45], two potential issues are relevant for our formulation: (i) under maximum-likelihood estimation (MLE), are the drift and diffusion parameters governing the latent-position transition probability recoverable, and (ii) can dynamic regimes be correctly classified? A practical constraint is that replay events are brief with a typical length of 100–250 ms and recordings comprise only dozens to a few hundred place cells. This relatively limited quantity of data may make estimation challenging. Thus, it is materially important to evaluate the performance of our inference procedures under conditions that are relevant to this experimental constraint.

**Fig 3:**
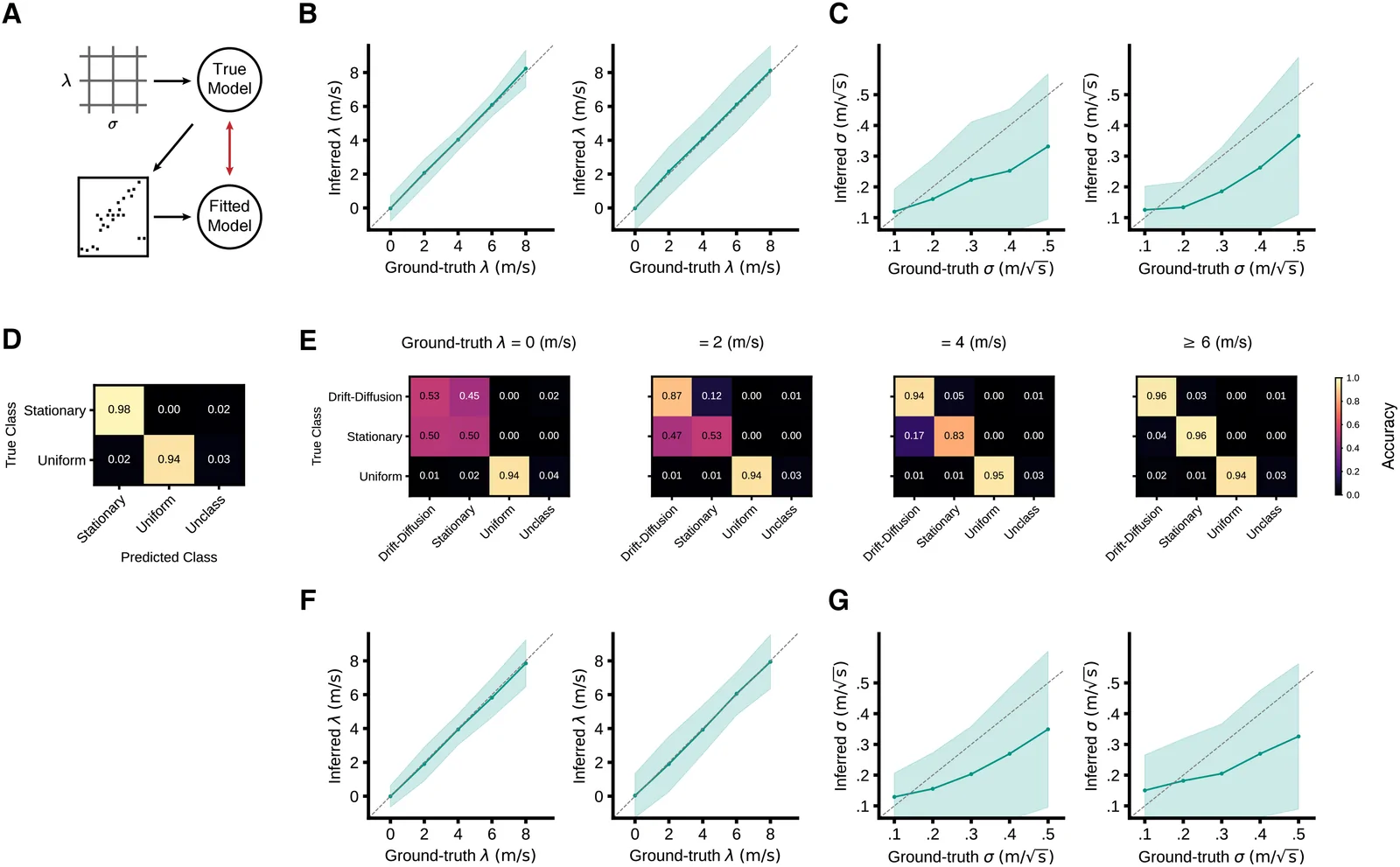
Model identifiability under realistic experimental conditions. **A**. Simulation and recovery pipeline. Two simulation settings were used. In the pure drift-diffusion setting **(B,C)**, ground-truth drift and diffusion parameters, (λ,σ), were sampled on a grid and used to generate latent trajectories on a 2 m linear track. Each trajectory generated one 150 ms SWR population event from *N* = 120 simulated place cells with place fields that uniformly tiled the track. In the regime-switching setting **(D–G)**, each simulated SWR was formed by concatenating two 150 ms segments whose ground-truth dynamics were sampled from drift-diffusion, stationary, or uniform regimes. Drift-diffusion segments used the same trajectory-generation procedure as in the pure drift-diffusion setting, whereas stationary and uniform segments were generated from their corresponding transition regimes. Each simulated event was analyzed with the inference pipeline to estimate marginal posteriors and, when drift-diffusion dynamics were present, the drift-diffusion parameters $\hat{\lambda }$ and $\hat{\sigma }$. Dynamics posteriors were converted to discrete inferred regime sequences, $\left\{{\hat{c}}_{t}\right\}$, using the classification rule in Fig 2B–2H and compared with the ground truth. **B**. Recovery of λ in the pure drift-diffusion setting with σ fixed at 0.2 (left) or 0.4 (right) $m/\sqrt{s}$. **C**. Recovery of σ in the pure drift-diffusion setting with λ fixed at 0 (left) or 2 (right) m/s. **D**. Confusion matrix for regime classification in switching events that contained only stationary and uniform segments. **E**. Confusion matrices for switching events containing a drift-diffusion segment, grouped by ground-truth drift: λ= 0, 2, 4, or ≥ 6 m/s. Color indicates classification accuracy. **F**. Recovery of λ in the regime-switching setting, restricted to events containing drift-diffusion dynamics, with σ fixed at 0.2 (left) or 0.4 (right) $m/\sqrt{s}$. **G**. Recovery of σ in the regime-switching setting, restricted to events containing drift-diffusion dynamics, with λ fixed at 0 (left) or 2 (right) m/s. In **B**, **C**, **F**, and **G**, solid curves show the mean over 100 repeats, shaded bands show ±1 standard deviation, and dashed diagonal lines indicate perfect recovery.

We first validated parameter identifiability for the basic drift-diffusion model without switching dynamics. We generated latent position trajectories from a two-dimensional grid of drift and diffusion values, then simulated spike trains from synthetic place fields conditioned on each trajectory. By fitting our model to these synthetic data, we can assess how well the model parameters can be recovered (Fig 3A). In general, both drift and diffusion parameters were satisfactorily recovered (Fig 3B and 3C). We found that the diffusion parameter was considerably more variable than the drift, and the variance of the diffusion parameter tended to increase with the true diffusion magnitude. We also found that the diffusion estimates tend to be underestimated because each replay event only contains a limited number of time steps (see S7 Text for a further discussion on the origin of this estimation bias).

We next evaluated identifiability in the switching HMM, the model that enables inference of switching between dynamical regimes. As before, we simulated latent trajectories with regime switches and generated spike trains from synthetic place fields, then optimized the full model. We first asked whether dynamics classes could be correctly decoded. We found that fitting yielded robust regime classification overall, as shown by confusion matrices comparing inferred and true regimes for simulated SWRs (Fig 3D and 3E). As expected, when both drift and diffusion are near zero and the observed replay sequence is short, the separability between drift-diffusion and stationary regimes is reduced. With a substantial drift magnitude, separability improves and classification accuracy between drift-diffusion and stationary increases markedly (Fig 3E). Consistent with the basic drift-diffusion analysis, the full model also yields accurate parameter recovery. MLE estimates of both drift and diffusion recover the ground truth across simulated conditions reasonably well (Fig 3F and 3G). However, there did remain modest underestimation of the diffusion parameter, attributable to short events. Together, these results show that our full model can reliably identify the dynamical regimes and the drift-diffusion parameters under experimentally realistic data sizes.

**Comparison to prior methods.** We compared our modeling framework with the traditional two-step analysis procedure, which first uses Bayesian decoding to map the neural population activity onto the posterior distribution over positions [34], then estimates descriptive statistical parameters (such as absolute correlation, slope of replay trajectory, maximum jump distance) [46,47] from the resulting posteriors (see S1A Fig). Using simulated data, we compared the drift and diffusion parameters to the key metrics used in prior studies. Based on extensive simulations, we found that, while the three key metrics may capture some structure in replays as conceived when they were developed, each measure is relatively confounded with the estimated speed and quality of the candidate replay event (see S1 Text and S1 Fig for detailed explanation). Thus, in practice the traditional two-step approach may not allow for an unambiguous interpretation of the replay dynamics. In contrast, our method adopts a more principled approach, namely explicit modeling of drift-diffusion, as a means to systematically disambiguate the speed (drift) and quality (diffusion) of a replay event. Importantly, this approach enables parameter estimation in a single step, allowing more principled modeling of uncertainty.

### 2.3 Applications to experimental data to investigate open questions about replay

We next apply our modeling framework to investigate the structure and dynamics of hippocampal replay. We will investigate three questions that remain unresolved in research on hippocampal replay. First, does hippocampal replay depict trajectories that evolve at real-world speeds or is it even stationary [25]? Second, does hippocampal replay evolve similarly to Brownian motion (random walk) [31]? Third, how does the structure of the hippocampal “preplay” events (during pre-run sleep) compare with replay events [32,46]? We analyzed a previously published dataset [33] that includes nine dorsal CA1 recording sessions from four rats navigating on a one-dimensional linear track (see Fig 1A and 1B for the task paradigm). For our main analysis, candidate SWR events were detected from LFPs and were subsequently restricted to population-active intervals for further analysis (similar to [33]; see Methods). Awake-rest and sleep SWRs were analyzed separately (a total of N = 1,412 awake SWRs and 15,111 sleep SWRs from 4 rats). In addition, we analyzed candidate population-burst events detected using multiunit activity (MUA) [30,48,49], and found qualitatively similar results (see Supplementary information).

**Fig 4:**
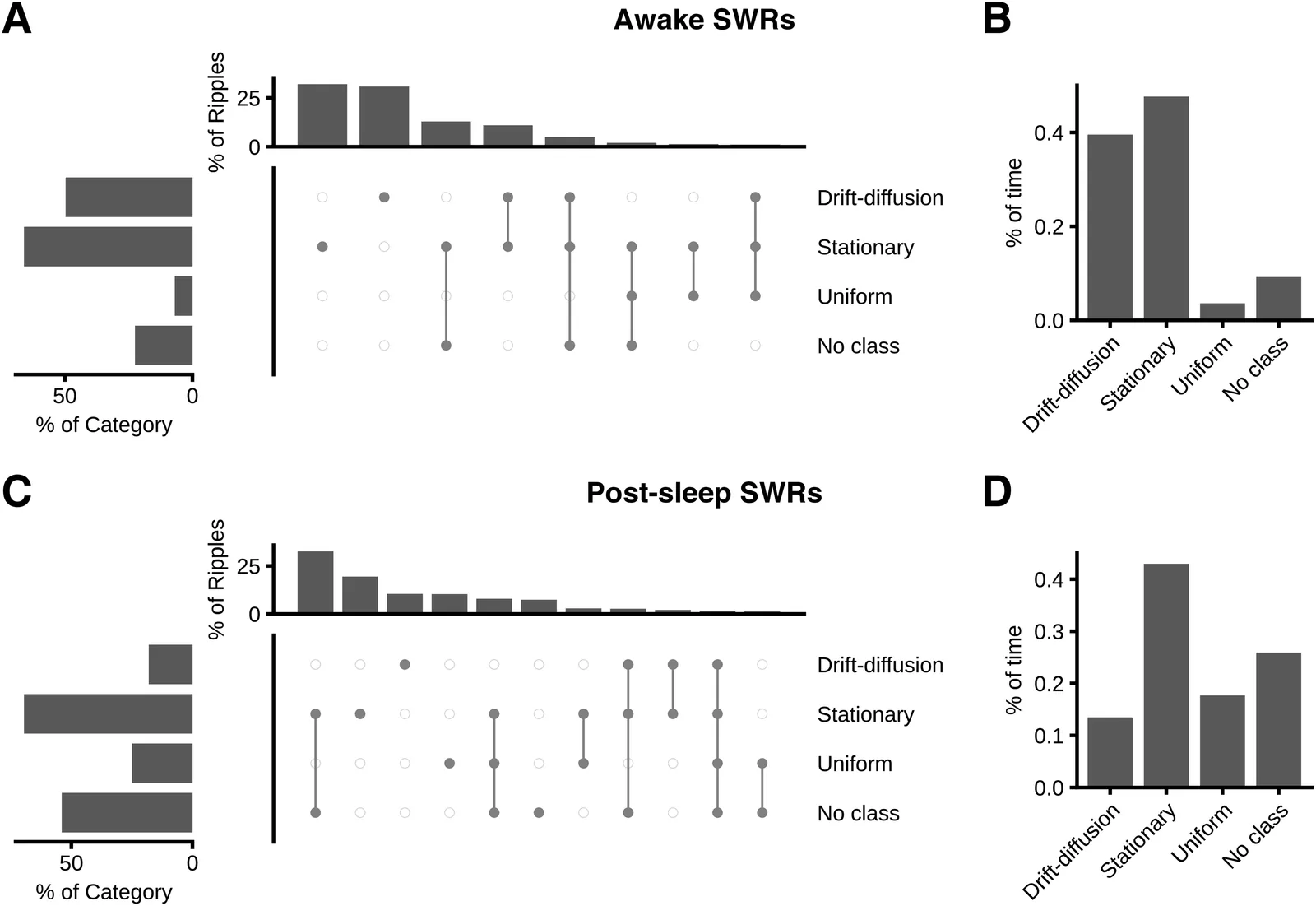
Composition of inferred spatiotemporal dynamics in awake and post-sleep SWRs. The marginal posterior of dynamics of each SWR was assigned a class label following the procedure described in Fig 2. For event-level summaries, a dynamic class was counted as present in an SWR only when it persisted for at least two consecutive 10 ms bins. **A**. UpSet-plot [50] summary of dynamic-class combinations in awake SWRs. The top bar plot shows the percentage of SWRs exhibiting each class combination, as indicated by the dot matrix below. Filled dots mark classes present in the combination, connected filled dots indicate co-occurring classes within the same SWR, and open dots mark absent classes. The left horizontal bar plot shows the percentage of SWRs containing each individual class. **B**. Fraction of awake SWR time bins assigned to each dynamic class. **C**. UpSet-plot summary of dynamic-class combinations in post-sleep SWRs, using the same conventions as in **A**. **D**. Fraction of post-sleep SWR time bins assigned to each dynamic class. Only class combinations occurring in more than 1% of SWRs are shown.

As an initial step of the analysis, we fit our full switching dynamics model to neural population responses during SWRs collected in [33]. A summary of the results is shown in Fig 4. An SWR event is considered to have one dynamic segment if it contains at least two consecutive 10 ms bins with a given dynamic label (see the criterion in Fig 4). We find that 30.81% of awake SWRs and 10.45% of post-sleep SWRs in this dataset can be classified as drift-diffusion dynamics only. In addition, about 18.91% of awake SWRs and 7.60% of post-sleep SWRs are best described as a mixture of drift-diffusion and other types of dynamics. Overall, the method classifies 49.72% of awake SWRs and 18.05% of post-sleep SWRs as containing at least a segment of a drift-diffusion period (i.e., at least 20 ms). These results are generally consistent with previous reports on replay, analyzed using standard Bayesian decoding methods [4,48]. Compared to previous methods, ours enables a more principled characterization of the rich dynamics within individual events (see examples in Figs 2B–2H and 6A, and additional examples in S4 Fig).

**Application 1: Do most replay events exhibit real-world moving speed or even remain stationary?**

Recent work suggests that a large fraction of SWR events were either stationary or had a replay speed that is comparable to the animal’s moving speed (< 1 m/s) [25]. This result seems to contrast with many previous reports showing that replay is compressed temporally, and its speed is often ten times faster than the animal’s movement speed [3,7,48].

**Fig 5:**
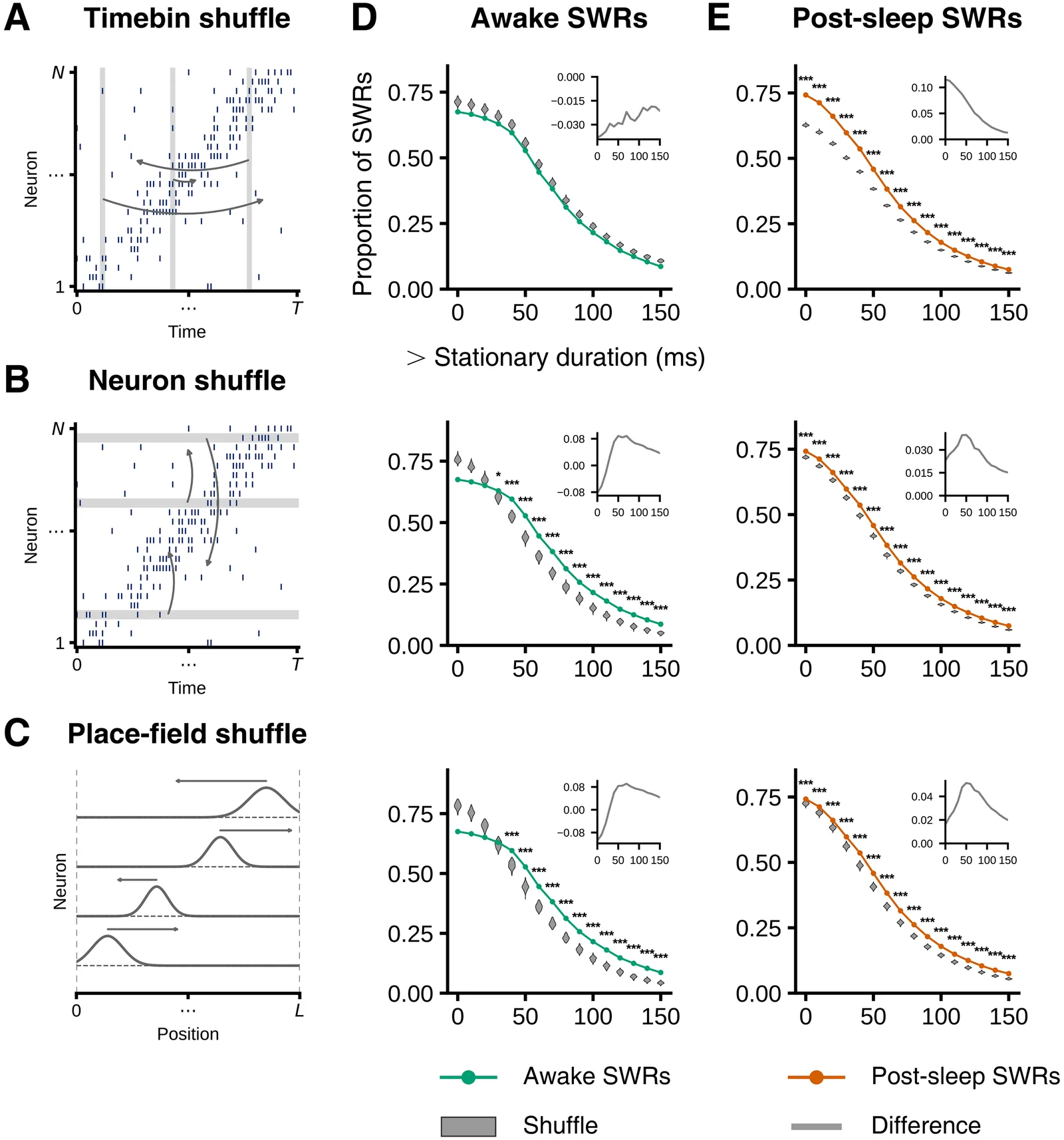
The fraction of stationary events in awake and post-sleep SWRs slightly exceeds chance levels. **A–C**. Schematic of the three shuffle controls used throughout the shuffle analyses. **A**. Time-bin shuffle, which permutes time bins within each SWR and disrupts temporal ordering while preserving the population activity in each time bin. **B**. Neuron-identity shuffle, which permutes neuron indices and disrupts the correspondence between spiking activity and place-field order. **C**. Place-field rotation shuffle, which circularly shifts each neuron’s place field along the linear track while preserving field shape. **D,E**. Real-versus-shuffle comparison of stationary duration in **D** awake and **E** post-sleep SWRs. Rows correspond to the shuffle controls in **A–C**: time-bin shuffle, neuron-identity shuffle, and place-field rotation shuffle. For each SWR, stationary duration was defined as the total time assigned to the stationary regime from the marginal posterior of dynamics. Curves show the proportion of real SWRs whose stationary duration exceeded each threshold on the x-axis. Gray violins show the corresponding distribution from 120 shuffle repeats per SWR. Insets show the difference between the real proportion and the mean shuffled proportion across thresholds, where positive values indicate more stationary structure in the data than expected under the shuffle. Asterisks indicate one-sided permutation tests comparing real data against the shuffle distribution (*p < 0.05, **p < 0.01, ***p < 0.001).

In light of this controversy, we next focus on investigating the stationary dynamics of SWRs. While our model fitting results indicate that 47.65% of awake SWR time and 42.95% of post-sleep SWR time exhibit stationary dynamics and seem to be consistent with the conclusion of [25], crucially, these results need to be interpreted together with the chance levels, *i.e.,* the fraction of the stationary dynamics inferred from the shuffled data. We consider three shuffle procedures (Fig 5A–5C). First, time-bin shuffle permutes the spike raster of each SWR across time bins, preserving per-neuron spike counts while disrupting within-event temporal structure. Second, neuron-identity shuffle randomly shuffles spike trains across neurons, preserving the spiking profile of each neuron but breaking the neuron-position mapping. Third, place-field rotation shuffle circularly shifts the place field of each neuron by an independent random offset. We compared the results obtained from real data and three shuffles. At a threshold of more than 50 ms total stationary duration, 52.76% of awake SWRs exceeded the threshold, compared with 55.65% for the time-bin shuffle, 43.93% for the neuron-identity shuffle, and 44.44% for the place-field rotation shuffle. Thus, for awake SWRs, the real fraction was 2.89 percentage points below the time-bin shuffle but 8.83 and 8.32 points above the neuron and place-field shuffles. For post-sleep SWRs, 45.82% of real events contained more than 50 ms total stationary duration, compared with 38.31%, 41.84%, and 40.68% for the three controls, corresponding to differences of 7.51, 3.98, and 5.14 percentage points (Fig 5D and 5E). Together, these results suggest that only a small fraction of SWR events are *bona fide* stationary events, and this fraction is much smaller than that reported in [25].

**Fig 6:**
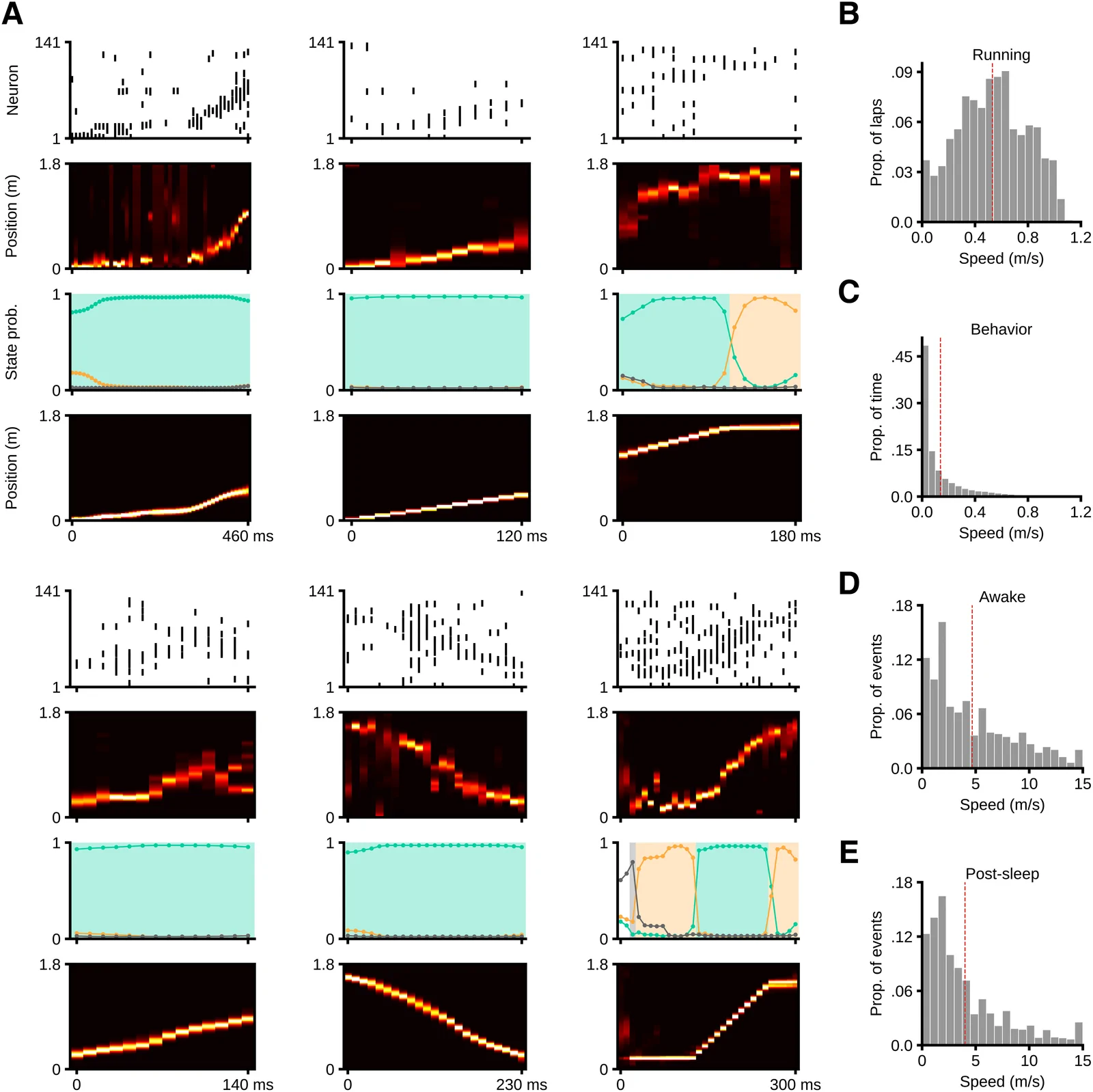
Replay trajectories are substantially faster than overt behavior. **A**. Representative events with different replay speeds. Plotting conventions follow Fig 2B–2H: spike raster, memory-less Bayesian position posterior, switching-HMM dynamics posterior, and switching-HMM position posterior are shown from top to bottom for each event. **B**. Distribution of per-lap running speeds on the linear track. Running speed was estimated for each traversal by fitting a linear regression to position as a function of time. **C**. Distribution of instantaneous behavioral speeds during running. Behavioral speed was estimated from the finite-difference derivative of tracked position. **D**. Distribution of replay speeds for awake events classified as drift-diffusion events. **E**. Distribution of replay speeds for post-sleep events classified as drift-diffusion events. Replay speed was estimated from the inferred drift rate. Drift-diffusion events were defined as events with at least five consecutive 10 ms bins assigned to the drift-diffusion regime. Red dashed lines indicate distribution means: running, 0.531 m/s; behavior, 0.138 m/s; awake replay, 4.677 m/s; post-sleep replay, 4.002 m/s.

Next, we examine a subset of SWR events with marginal posterior of drift-diffusion dynamics exceeding 0.7 for at least 50 ms. These events are particularly interesting because they exhibit strong sequential structures and thus are consistent with the classic notion of replay events. These selected events account for 44.62% of awake SWRs (N = 630) and 14.76% of post-sleep SWRs (N = 2,231). Fig 6A shows example SWR events with varying replay speed. The distribution of the replay speed inferred for these events is shown in Fig 6D and 6E, with a mean of 4.68 m/s for awake events and 4.00 m/s for post-sleep events. The speed of these replay events is substantially higher than that of running (Fig 6B) or movement periods (Fig 6C). These results are insensitive to the exact criteria used to select these events. Thus, for the subset of SWR events that exhibit substantial drift-diffusion dynamics, the speed of the drift is one order of magnitude faster than the running speed, generally consistent with earlier reports on replay [8,48].

Our results suggest that the subset of SWR events (~45% for awake, and ~15% for post-sleep) with substantial sequential structure indeed exhibit a speed that is approximately an order of magnitude faster than the animal’s typical running speed, consistent with classic studies of replay [8,48]. Our results also provide evidence for the existence of stationary dynamics in a small fraction of SWR events [25], by carefully taking into account the chance levels based on the shuffled controls.

**Application 2: Does hippocampal replay in a 1-D environment resemble Brownian motion?**

Although earlier studies of replay emphasize the linear-sequential structure of replays, we found that the decoded trajectories of many SWR events have substantial variability compared to the path of the animals’ behavioral trajectories. Intriguingly, a recent study [31] on hippocampal activity argues that sleep replays after random foraging in a 2-D open arena resemble Brownian motion (but see [26]). So far, it remains unclear whether similar results hold in other experiments, in particular in 1-D environments, where replays are typically studied.

One of our results reported above seems to contradict the proposition that Brownian motion generally describes the dynamics of SWRs, that is, a considerable proportion of SWRs exhibit a substantial drift (Fig 6D and 6E). However, it remains possible that the noise may cause the empirically estimated drift to substantially exceed zero even when the trajectories are sampled from a pure diffusion process. To rule out this possibility, we generated a null distribution of the estimated speed from the diffusion process, by sampling and analyzing trajectories from diffusion models (S5 Fig). We find that this null distribution is substantially different from the distribution obtained from the rat data. In particular, the speeds obtained from the simulated diffusion process are substantially slower. These results suggest that the subset of drift-diffusion events identified by our method is unlikely to have been characteristically generated by Brownian motion.

**Fig 7:**
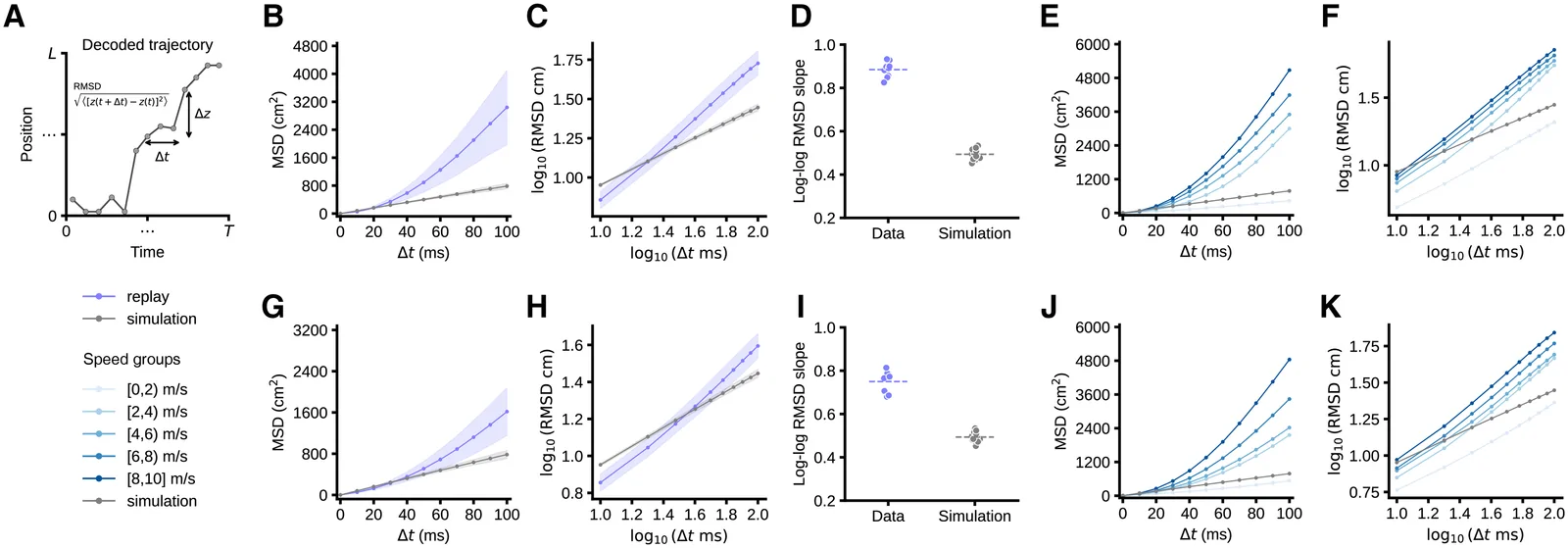
Replay trajectory differs from pure diffusion dynamics (Brownian motion), and is more consistent with drift-diffusion. **A**. Schematic of the displacement analysis. For a decoded latent trajectory, displacement was measured as Δz=|z(t+Δt)−z(t)| over time lags Δt. Mean-square displacement (MSD), $\langle \Delta z^{2}\rangle$, and root-mean-square displacement (RMSD), $\sqrt{\langle \Delta z^{2}\rangle }$, were then computed across trajectory samples. **B–F**. Displacement analysis applied to awake SWRs classified as drift-diffusion events. Replay trajectories were decoded under a fixed pure-diffusion model (σ=0.8 m$/\sqrt{s}$) and compared with pure Brownian-motion simulations [26]. **B**. MSD as a function of Δt for replay (purple) and simulated diffusion trajectories (gray). **C**. Log-log RMSD as a function of Δt for the same replay and simulation data. **D**. Slopes from linear fits to the log-log RMSD curves, with each point representing one recording session or one simulated session. Dashed horizontal lines indicate session means. **E**. MSD as a function of Δt for awake replay trajectories grouped by inferred replay speed. **F**. Log-log RMSD scaling for replay trajectories grouped by inferred replay speed. **G–K**. Displacement analysis applied to post-sleep SWRs classified as drift-diffusion events. **G**. MSD for replay and simulated diffusion trajectories. **H**. Log-log RMSD for replay and simulated diffusion trajectories. **I**. Slopes from linear fits to log-log RMSD curves. **J**. MSD by replay-speed group. **K**. Log-log RMSD scaling by replay-speed group. Speed groups are λ∈[0,2), [2,4), [4,6), [6,8), and [8,10] m/s. In **C**, **D**, **H**, and **I**, solid lines and shaded bands show the mean ±1 standard deviation across sessions. Mean log-log RMSD slopes were 0.885 for awake replay, 0.750 for post-sleep replay, and 0.493 for simulated Brownian trajectories.

To make the results directly comparable to prior work, we further analyzed the data using an approach generalized from [31], which examines how the root-mean-square displacement (RMSD) scales with the time interval (Fig 7A). Their key idea is that Brownian motion predicts that RMSD should increase linearly with the square root of the time interval ($\sqrt{\Delta t}$), i.e., $\text{RMSD}\propto \sqrt{\Delta t}$. Empirically, Stella et al. [31] reported that the slope between these two quantities is reasonably close to 0.5 in a log-log plot. To evaluate this prediction independently, we generalize the previous analysis framework from [31] to more general drift-diffusion dynamics. Importantly, if the replay trajectory follows a drift-diffusion process, the mean-squared displacement (MSD) should be characterized by a quadratic rather than a linear function, that is, $\text{MSD}=(\lambda \Delta t{)}^{2}+\sigma^{2}\Delta t$. Under this view, Brownian motion is only expressed in the special case of zero drift, in which $\text{MSD}=\sigma^{2}\Delta t$.

In examining log-log plots of RMSD vs. Δt (limited to drift-diffusion events, namely events with drift-diffusion time lasting at least 50 ms), we observed a mean slope of 0.89 for awake drift-diffusion events and 0.75 for post-sleep drift-diffusion events, both substantially greater than 0.5. The results are consistent across nine sessions (std = 0.034 for awake and std = 0.048 for post-sleep) and deviate substantially from the predictions of Brownian motion (Fig 7C–7D and 7H–7I). Importantly, to avoid biases toward finding systematic drifts, we used a fixed diffusion model (with σ = 0.8 m$/\sqrt{\text{s}}$, zero drift) to decode the trajectories [26].

Our theoretical analysis reveals one important limitation of the approach based on the slope between RMSD and time interval in the log-log space. The issue is that, while the slope in the log-log space may be adequate to recover the exponent α for a power-law relation $\text{MSD}=C(\Delta t{)}^{\alpha }$, it is insufficient to test the more general prediction of drift-diffusion models in the form of $\text{MSD}=(\lambda \Delta t{)}^{2}+\sigma^{2}\Delta t$. To overcome this limitation, we further examined how MSD scales with time interval in the original scale for drift-diffusion events. Consistent with the prediction of the drift-diffusion dynamics, we find that the relationship between MSD and time interval can be well described by a quadratic, rather than a linear, relationship (Fig 7B and 7G). A further prediction of the drift-diffusion model is that the relationship between MSD and time interval should be systematically modulated by the drift rate. To test this, we sorted and separately analyzed the drift-diffusion SWR events with different drift rates, finding an even stronger quadratic relationship for the events with higher speed (Fig 7E and 7J). In the log-log plots, this yielded a slope that was closer to 1 for high speed events (Fig 7F and 7K). These results provide strong empirical support for the drift-diffusion dynamics in hippocampal replay.

A key advantage of our modeling approach is that it potentially enables the identification and characterization of different types of replay dynamics. Indeed, our empirical results in Fig 4 suggest that SWRs exhibit different types of dynamics. If these different types of events are indeed meaningful, one substantive prediction, generalizing the one suggested specifically for Brownian motion above, would be systematically different scaling relationships between MSD and time interval. To test this prediction, we additionally analyzed the subset of events that contain only stationary dynamics, as well as the subset of events that contain only uniform dynamics. Consistent with our prediction, for SWRs with stationary dynamics (S9A and S9C Fig), the slope in the log-log plot is substantially smaller than 0.5 (awake: mean = 0.27, std = 0.16; post-sleep: mean = 0.21, std = 0.11). Furthermore, for events with uniform dynamics (S9D Fig), the slope is also smaller than 0.5 (post-sleep: mean = 0.39, std = 0.07). When all awake SWR events are combined, the resulting slope between RMSD and time interval in the log-log plots (mean = 0.82, std = 0.06) is substantially higher than 0.5. Interestingly, for post-sleep SWRs, the corresponding slope is closer to 0.5 (mean = 0.57, std = 0.05). However, given the heterogeneity of the dynamics reported above, an overall slope close to 0.5 is insufficient to support the Brownian motion hypothesis for post-sleep SWRs. Together, these results suggest that the scaling relationship between RMSD and time varies across different types of dynamics, highlighting the importance of analyzing different types of dynamics separately.

**Application 3: Is there evidence for preplay during SWRs?**

It has been suggested that the hippocampal “preplay” sequences of neural activity occur before the animal has any experience in a given environment [32,51]. While preplay potentially has important implications for the mechanisms and functions of the hippocampus, the existence and prevalence of preplay remain controversial [46]. This is in part due to the difficulty of precisely characterizing the dynamics of the SWR events. Using our method, we sought to re-examine the evidence for preplay, and if preplay exists, how its dynamics compare with replay dynamics after spatial experience in a given environment.

We analyzed the SWRs during pre-run sleep (data from [33], a total of N = 6,749 events from 4 rats). We first quantified the composition of dynamics (see S10 Fig). For pre-run sleep, drift-diffusion dynamics account for 5.83% of total SWR time, which is substantially lower than in awake rest (39.53%) and post-run sleep (13.46%). At the event level, 5.54% of pre-sleep SWRs can be classified as drift-diffusion dynamics only and 9.82% contain at least one drift-diffusion segment. These fractions are again much smaller than those in awake rest (30.81% and 49.72%) and post-run sleep (10.45% and 18.05%), respectively. Thus, pre-sleep SWRs exhibit less sequential structure than post-sleep SWRs.

**Fig 8:**
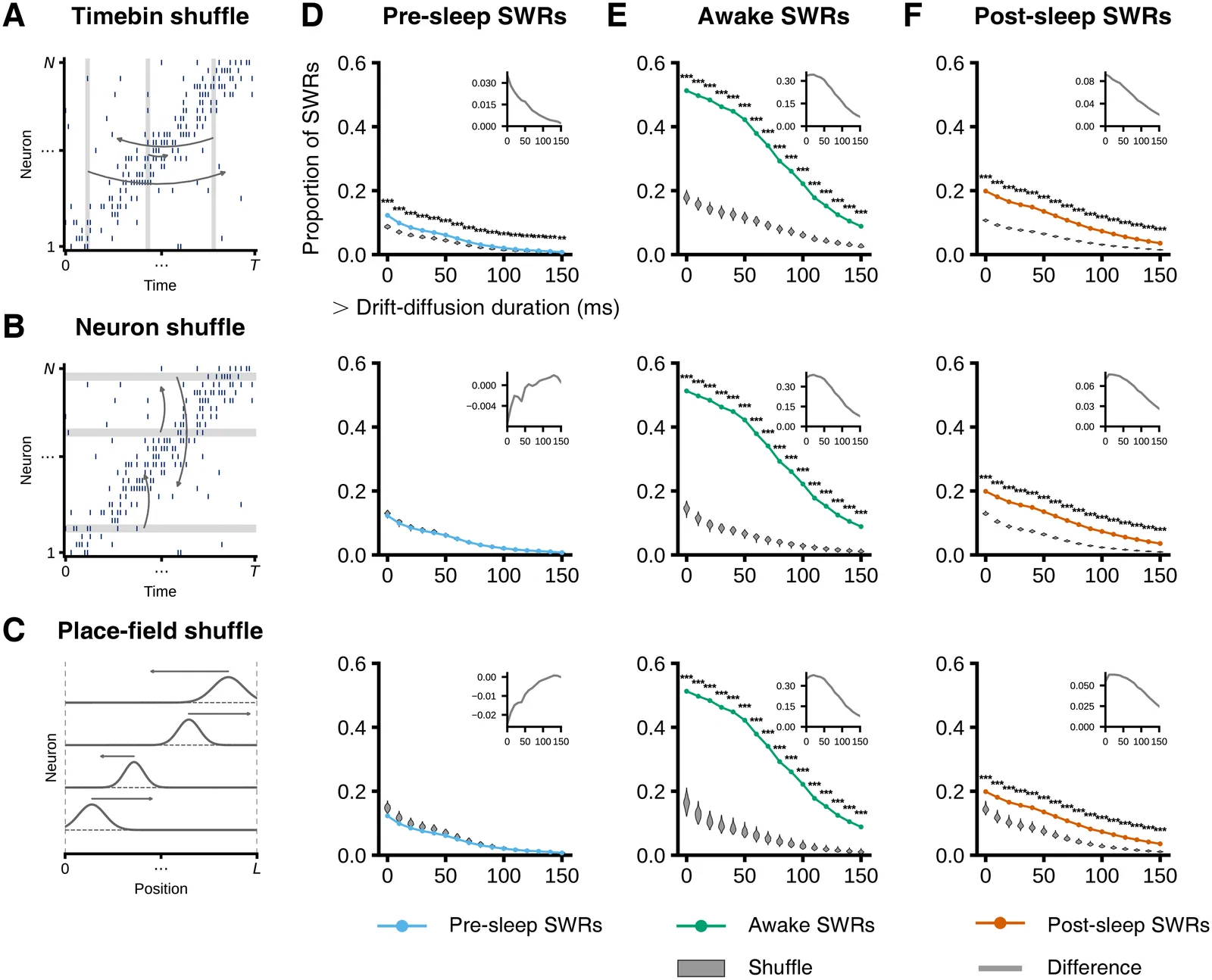
Preplay events show substantially weaker temporal structure than replay. **A–C**. Schematic of the same shuffle controls described in Fig 5: time-bin shuffle, neuron-identity shuffle, and place-field rotation shuffle. **D–F**. Real-versus-shuffle comparison of drift-diffusion duration in **D** pre-sleep, **E** awake, and **F** post-sleep SWRs. Rows correspond to the shuffle controls in **A–C**: time-bin shuffle, neuron-identity shuffle, and place-field rotation shuffle. For each SWR, drift-diffusion duration was defined as the total time assigned to the drift-diffusion regime from the marginal posterior of dynamics. Curves show the proportion of real SWRs whose drift-diffusion duration exceeded each threshold on the x-axis. Gray violins show the corresponding distribution from 120 sh...19 tokens truncated...ortion and the mean shuffled proportion across thresholds, where positive values indicate more drift-diffusion structure in the data than expected under the shuffle. Asterisks indicate one-sided permutation tests comparing real data against the shuffle distribution (*p < 0.05, **p < 0.01, ***p < 0.001).

To assess whether the observed fraction of “preplay” events is significantly higher than chance levels, we conducted the three shuffle analyses mentioned earlier (time-bin shuffle, neuron-identity shuffle, and place-field rotation shuffle) on preplay using data from pre-run sleep periods [46] (Fig 8A–8C). We further compare the results with those obtained from the shuffle analyses on awake and post-sleep SWRs. Taking advantage of our drift-diffusion framework, we used the duration of drift-diffusion dynamics within an event as a metric to characterize the sequentiality of an event (Fig 8D–8F). Across a range of thresholds, we computed the fraction of SWR events that exceeded each threshold to assess statistical significance. Based on either neuron-identity shuffle or place-field rotation shuffle, we did not find evidence for preplay, consistent with results reported in [46]. However, for time-bin shuffle, the fraction of events that exhibited sequentiality was higher than the chance level, consistent with [51]. These results may help reconcile the previous discrepancy on the existence of preplay from studies that used different shuffle procedures [46,51]. It is also interesting to note that the time-bin shuffle has been suggested to be relatively less conservative than the neuron-identity and place-field shuffles [20], which also underscores the finding of significant preplay structure as surprising.

When comparing pre-run sleep, awake, and post-sleep SWRs, we consistently find that the fraction of events exceeding any given threshold was higher for awake and post-run sleep SWRs than for pre-sleep. This suggests that at the level of individual events, pre-sleep SWRs also exhibited shorter drift-diffusion segments than awake and post-sleep SWRs, mirroring the pooled result in Figs 4 and S10. Awake and post-sleep SWRs showed differences from all three controls with strong significance (p < 0.001 for each; one-sided permutation test). In contrast, pre-sleep SWRs differed only from the time-bin shuffle (p < 0.001; one-sided permutation test) and not significantly from the neuron-identity or place-field rotation shuffles. These results suggest that replay after experience can be detected robustly, while preplay is detectable statistically only when neuron-position mappings are preserved, as in time-bin shuffle, but not in neuron-identity and place-field shuffles.

The results above suggest that hippocampal population activity during pre-sleep SWRs may be coordinated at a level that is higher than chance. We next examined whether such coordination happens at a timescale that is comparable to the classic replay events (typically around 100 ms). By taking the difference of the distribution of drift-diffusion durations between real data and shuffles (Fig 8D–8F inset), we obtained an estimate of the fraction of drift-diffusion events with relatively long durations. According to this metric, for awake and post-sleep SWRs, the fractions of events that exhibit drift-diffusion dynamics for longer than 100 ms based on time-bin shuffle are about 16.07% and 4.21%, respectively. For pre-sleep SWRs, this fraction is 0.64%. Thus, while hippocampal population activity during pre-sleep SWRs exhibits slightly higher sequentiality than time-bin shuffled control, long temporal sequences are rather rare.

Together, these results provide evidence for pre-existing structures in place-cell population activity before rats have spatial experience in a given environment. However, the level of coordination in these structures is substantially weaker than that seen after spatial experience (i.e., awake rest or post-run sleep). In particular, we found that long sequences during pre-sleep SWRs are rather rare and are much weaker than replay sequences after spatial experience.

## 3 Discussion

We have developed a new framework, conceptually and statistically, for analyzing and understanding the dynamics of replays. This framework is formulated based on drift-diffusion dynamics while allowing switching between several types of dynamics. Drift-diffusion models have been widely used to model the neural basis of decision-making [52–54]. Here we show that the drift-diffusion framework also provides a powerful framework to study the dynamics of the hippocampal activity during SWRs. Compared to standard methods for analyzing replays, there are several important differences that make our method advantageous. First, using a generative modeling approach, our method enables principled probabilistic inference on replay structure while fully taking into account the estimation uncertainty. Second, our framework explicitly models speed and quality of classic replay events (i.e., drift-diffusion events) with drift and diffusion parameters. Third, with the capability of switching between different types of dynamics, our method is able to characterize the putatively rich dynamics in a broader set of hippocampal SWRs, which continues to remain an important open question. Evaluation and comparison of our method to others reveal that our method allows for more direct and unambiguous interpretation of replay dynamics.

Application of our modeling framework to hippocampal population recording data revealed several insights that helped resolve controversies about replays. First, our results clarify the recent debate on the replay speed and stationary dynamics during SWRs. Consistent with the results reported in [25], we find that some SWR events contain segments of stationary dynamics. However, the fraction of *bona fide* stationary events is likely much smaller than that reported in [25], when considering the chance levels obtained from the shuffled data. Somewhat surprisingly, a large fraction of events from the shuffle data exhibit stationary dynamics. This suggests that stationary dynamics observed in decoding may arise from multiple sources and by itself cannot be interpreted as conclusive evidence for strongly coordinated neural activity with slow dynamics. While the exact reasons for observing many stationary events in the shuffle data remain to be determined in future work, we hypothesize that the following two factors may contribute. First, the heterogeneity in the distribution of place fields may bias the decoded posterior, causing it to remain concentrated at the same spatial position across consecutive time bins. Second, if a small subset of neurons consistently exhibits high firing rates during segments of SWRs, a stationary posterior distribution over position may arise in decoding. We also find that, for a subset of SWR events that exhibit consistent sequential structures, the speed is about 10 times faster than the animal’s running speed. Thus, our results shed light on the discrepancy between the results in [25] and those of classic studies of replay. Our results also highlight the rich and diverse types of neural dynamics during SWRs. The role that different types of neural dynamics play in learning and memory remains unclear–an important question that needs to be determined in future work.

Second, our results clarify the dynamics of SWRs, at least on 1-D linear tracks. By carefully analyzing the scaling relationship between the MSD and time interval, we found that the two follow a quadratic relationship for events with high sequentiality. This result is highly consistent with the prediction of drift-diffusion dynamics, but not with Brownian motion as proposed in [31]. Importantly, we also found that different types of events exhibited different scaling relationships. For stationary events and uniform events, the relationship is sublinear. These results provide further support for the distinctness of different types of dynamics identified using our method. Our results suggest that it is more informative to analyze the scaling relationship between MSD and time interval in the original scale, rather than the log-log scale proposed previously [31]. The latter may confound potential contributions from diffusion and drift. While our results are based only on data collected in 1-D environments and do not directly contradict the 2-D results reported in [31], they do motivate revisiting or further exploration of replay dynamics in 2-D and additional environment and behavioral settings. In particular, it would be interesting to examine or re-examine the 2-D data to see whether a general quadratic relation also holds in 2-D for the replay events they identified during post-sleep SWRs [31]. It would also be important to examine whether there is substantial heterogeneity in the dynamics of SWR events in 2-D. One possibility is that, for SWRs in 2-D environments, the dynamics are also heterogeneous, and combining all events together might accidentally produce an overall slope (between RMSD and time) close to 0.5 in the log-log scale. If true, this would be consistent with our results on post-sleep SWRs in 1-D, for which a slope of 0.57 was obtained when pooling the data with different types of dynamics together. Regardless, our results demonstrate that the dynamics of SWRs are diverse, and cannot be adequately described by Brownian motion in 1-D environments.

Third, regarding the question of the existence of pre-existing temporal structures before spatial experience in a given environment [32], we only found weak evidence suggestive of sequential structure in pre-run sleep, by comparing the data distribution to the null distribution generated through time-bin shuffle [47,51]. No sequential structure was detected based on two other shuffle methods (neuron shuffle and place-field shuffle). Furthermore, the results based on time-bin shuffle show that long drift-diffusion sequences that are comparable to classic replay events with a timescale of 100 ms are exceptionally rare (less than 1% of SWRs in pre-run sleep). Additional caution needs to be taken, because time-based shuffle may destroy the temporal structure in individual neurons (*i.e.,* the firing rates of individual neurons become less smooth over time) and thus may provide a weaker null distribution and inflate the estimated sequential structure [55]. Furthermore, given that the animals may have been exposed to environments with somewhat similar spatial structure, sequences observed during pre-sleep SWRs may still reflect a type of replay of analogous experiences [9,11,56]. Exactly what properties of the pre-existing hippocampal circuits lead to the higher-than-chance sequential structure during pre-run sleep remains to be determined [57]. One possibility is that the structure during pre-run sleep is caused by the cellular correlation between “rigid” place cells that is preserved across environments [58].

Earlier studies have used state-space models to decode positions and reconstruct “mental” trajectories from hippocampal activity during running and sleep, although they did not explicitly characterize the spatiotemporal structure of replay [28–30,38,39]. More recently, state-space models have been applied to explicitly model dynamics during SWRs [25,26]. In particular [25], proposed a method for analyzing hippocampal replay by considering switching dynamics during decoding. There are two crucial differences between the method in [25] and the method presented here. First, the parameters of their method were manually specified rather than inferred from data. Second, their method does not model drift, in part as a consequence of the manual specification. Instead, it relies solely on Brownian motion as its spatially “continuous” dynamics to capture the sequential structure of replay events. As we have shown, Brownian motion is inadequate for capturing sequential replay dynamics. These limitations may introduce biases in estimating dynamics and cause decoding errors in estimating positions. For example, the replay speeds based on their method could be underestimated if the replay in neural data has a substantial drift, which does not conform to the manually specified structure in the model. In contrast, our method directly estimates two essential model parameters from individual replay events and is thereby more flexible. Another recent study [26] formulated HMM models to study transition dynamics in 2-D open-field environments. They found that the momentum of the trajectory is important for fitting replay events. One important difference is that their model does not allow for switching between different types of dynamics. Also, their model is a second-order HMM; our model is first-order. From what we have shown, a first-order switching HMM appears sufficient to capture the key dynamical patterns of replay. Moreover, its dynamics and parameters (e.g., drift and diffusion) map directly onto first-order transition statistics, leading to a more transparent interpretation of how replay trajectories evolve over time. Our first-order formulation also simplifies the probabilistic inference procedure and potentially improves parameter identifiability, leading to more stable and accurate characterizations of replay dynamics.

Extensions and additional applications of the proposed modeling framework are possible. First, our current implementation primarily deals with a 1-D track or a circular environment, two popular environments that have been used to study hippocampal replay. Other types of environments have also been used in the study of replay, such as 2-D open fields and 1-D environments with more complex geometry. Adaptation of our approach to these environments should be feasible, but would require modifications to the probabilistic transition matrix for latent positions. In particular, extending the model to a 2-D environment would require additional considerations on how to parameterize drift and diffusion in a two-dimensional environment jointly and how to detect jumping events within the same probabilistic framework. Second, another natural extension would be a switching drift-diffusion model that switches between multiple drift-diffusion processes with distinct parameters or other types of dynamics (such as Levy flight). However, practically the limited amount of data available per SWR event may prevent one from estimating the parameters reliably. In this paper, we therefore adopt a more parsimonious model by explicitly including two stereotyped dynamics (i.e., stationary and uniform sampling) alongside a single drift-diffusion component. This choice preserves tractability while maintaining flexibility to capture the spatiotemporal heterogeneity of individual SWRs. Crucially, by allowing stationary and uniform regimes in the model, the method can isolate SWR segments with substantial drift and spatial coherence. Future experiments with much larger numbers of neurons recorded simultaneously from the hippocampus may enable reliable inference of parameters from these even more sophisticated models. Third, the present model assumes that the latent state of neural activity at any moment represents a position in current environment. Neural activity representing another environment or only weakly representing spatial information of the current environment may also be present in the data. To further improve the model, one can incorporate a “not-on-track state” or, more generally, model the latent state as a distribution over position. Finally, while we only focus on replay sequences during SWRs, our method is in principle applicable to the study of theta sequences in rodents during running [59,60]. Theta sequences represent another important type of dynamical structure in the hippocampus [61–63] and entorhinal cortex [64], which may have equally important functional implications for the role of the hippocampus or other areas in cognition. Previous analysis typically relied on analyzing the averaged slope of the theta sequences. Using our method, one may potentially reveal the sequentiality and variability of the dynamics within individual theta sequences.

A better understanding of replay dynamics in the brain may help inform the development of better learning algorithms in artificial intelligence. Algorithms based on replaying past experiences have been developed [65] and shown to improve performance across a variety of tasks in reinforcement learning [66,67]. Yet most current AI algorithms still struggle in continual learning without catastrophic forgetting [68–70]. Developing brain-inspired algorithms to replay past experiences efficiently [71,72], or to sample from a generative model built on past experiences (i.e., generative replay) [73–75], remains a promising path forward [76,77]. More accurate and interpretable data-driven techniques, such as the one presented here, will likely be instrumental toward clarifying the exact role of replay in natural cognition, thereby informing brain-inspired replay mechanisms for continual learning in AI. Mechanistically, recent computational models show that firing-rate adaptation in continuous attractor neural networks (CANNs) can generate stationary, diffusive, and super-diffusive dynamics that resemble replays [78,79]. These studies address how such dynamics may arise from network computations, whereas the present framework estimates these dynamics statistically from population recording data, and thus is synergistic with recent mechanistic modeling.

Hippocampal SWRs have direct implications for cognitive functions. It was reported that disrupting SWRs in rodents impairs spatial memory in an AD-developmental mouse model [80] or spatial learning in wild-type [81], whereas enhancing SWRs may improve subsequent task performance [82]. Recent work shows that in a mouse model of Alzheimer’s disease, the reactivation of hippocampal activity during SWRs was disrupted [83]. Most prior work used simple metrics such as the frequency of SWRs. Using our method to analyze the detailed dynamics during different task conditions may reveal new insights into the relationship between the properties of SWRs and behavior. Finally, adapting our method to the online setting may enable the design of more targeted, dynamics-specific, SWR-based neurofeedback protocols for cognitive perturbation and enhancement [84,85].

## 4 Methods

### 4.1 Description of the datasets

We analyzed hippocampal recordings from the one-dimensional linear-track experiments of Pfeiffer and Foster (2015) [33]. The dataset consisted of nine recording sessions from four adult Long–Evans rats: four sessions from Janni, two from Harpy, one from Imp, and two from Ettin. In seven sessions, the behavioral protocol consisted of a pre-run sleep epoch in an isolated box, a run epoch on the linear track, and a post-run sleep epoch in the same box. In the other two sessions, an initial pre-run sleep epoch was followed by three repeated run and post-run sleep epochs. Run epochs lasted approximately 40 min, whereas pre- and post-run sleep epochs lasted 40–60 min. During run epochs, rats traversed a 1.8 m linear track that was 6.35 cm wide along most of its length and widened to 12.7 cm at each end. The track was bounded by 3.8 cm-high walls. Reward wells at both ends were filled outside the animal’s view with 200–250 μL of chocolate-flavored liquid, encouraging back-and-forth traversal. Dorsal CA1 activity was recorded while the animals moved on the track, and position and head direction were tracked at 60 Hz using an overhead camera.

### 4.2 Place field estimation

Place fields were estimated from neural activity during run epochs. Position and speed were first interpolated onto 50 ms time bins, and only bins in which the animal’s speed was at least 5 cm/s were used. The animal’s position was linearized along the track axis and discretized into 2 cm spatial bins. For each neuron *i*, spikes were accumulated within each spatial bin across the selected time bins, and spatial occupancy was computed as the total time spent in the same bin. The occupancy-normalized spike count yielded a firing-rate estimate in each spatial bin. The firing-rate vector was smoothed with a one-dimensional Gaussian kernel with a standard deviation of 4 cm, corresponding to two spatial bins. The resulting smoothed rate map, denoted $f_{i}(z)$, was used as the place field of neuron *i*. Putative inhibitory neurons were excluded, and a neuron was retained for subsequent analyses only if the peak of its smoothed place field was at least 1 Hz.

### 4.3 Sharp-wave ripple detection

Sharp-wave ripple (SWR) candidates were detected from the local field potential (LFP) using a ripple-band thresholding procedure based on Karlsson and Frank (2009) [9]. The LFP trace was band-pass filtered between 150 and 250 Hz with a second-order Butterworth filter. We then computed the instantaneous ripple-band amplitude as the absolute value of the Hilbert transform and smoothed this amplitude envelope with a Gaussian kernel (σ=12.5 ms). The smoothed envelope was z-scored within each session, and candidate events were defined as intervals in which the z-scored envelope crossed 3 standard deviations above the session mean. Event boundaries were extended to the nearest surrounding crossings of the session mean. Candidate events were retained only if their LFP-defined duration was greater than 50 ms and less than 2 s. For events occurring during run epochs, we further required the animal’s mean speed during the event to be below 5 cm/s. Events during pre-run and post-run sleep epochs were retained without a speed criterion. For downstream spike-train analyses, retained SWRs were projected onto non-overlapping 10 ms spike-count bins. The event window was then restricted to the population-burst interval spanning from the first 10 ms bin whose mean population firing rate exceeded 2 spikes/second/neuron to the last such bin, and the resulting interval was required to last at least 50 ms. To examine whether the results are robust to the event detection criterion, we performed a complementary analysis using population-burst events detected from multiunit activity (MUA) alone, without requiring ripple-band activity in the LFP. We found that the main results generally hold for events detected using MUA (see Supplementary information for details).

### 4.4 State-space modeling

#### 4.4.1 Transition dynamics.

We modeled the temporal evolution of latent position during SWRs using three classes of transition dynamics: drift-diffusion, stationary, and uniform transitions. Let $z_{t}$ denote the latent position in time bin *t*, and let the linear track be represen*t*ed by a finite grid $\left\{z^{1},...,z^{M}\right\}$. The continuous transition models below define densities for the latent position, which are evaluated on this grid to form the transition matrices used for inference. Let Δt denote the time-bin width.

**Drift-diffusion.** Drift-diffusion dynamics were defined by the continuous-time stochastic differential equation


$$\begin{array}{c} dz_{t}=\lambda \;dt+\sigma \;dW_{t} \end{array}$$

where λ is the drift parameter, σ is the diffusion parameter, and *W* is a Wiener process with $𝔼[dW_{t}]=0$ and $Var[dW_{t}]=dt$. Discretizing this process over one time bin gives the Gaussian transition density


$$\begin{array}{c} p(z_{t}|z_{t-1})=𝒩\;\left(z_{t};\;z_{t-1}+\lambda \Delta t,\;\sigma^{2}\Delta t\right) \end{array}$$

where $𝒩(z;\mu ,\sigma^{2})$ denotes the normal density with mean μ and variance $\sigma^{2}$. On the finite spatial grid, the corresponding transition weight from bin *i* to bin *j* is


$$\begin{array}{c} A_{ij}^{\left(DD\right)}=\frac{1}{\sqrt{2\pi \sigma^{2}\Delta t}}\exp\;\left[-\frac{(z^{j}-z^{i}-\lambda \Delta t{)}^{2}}{2\sigma^{2}\Delta t}\right] \end{array}$$

**Stationary.** Stationary dynamics constrain the latent position to remain fixed across adjacent time bins. In continuous time this corresponds to


$$\begin{array}{c} dz_{t}=0 \end{array}$$

and the discrete-time transition kernel is therefore


$$\begin{array}{c} p(z_{t}|z_{t-1})=\delta (z_{t}-z_{t-1}) \end{array}$$

where δ(·) is the Dirac delta. On the finite spatial grid, this is the identity transition matrix,


$$\begin{array}{c} A_{ij}^{\left(stat\right)}=\left\{ \begin{array}{cc} 1, & j=i,\\ 0, & j\neq i. \end{array}\right. \end{array}$$

**Uniform.** Uniform dynamics represent the absence of temporal structure: the latent position is resampled independently at each time bin. This model has no meaningful continuous-time limit, and is defined directly on the finite spatial grid as


$$\begin{array}{c} p(z_{t}=z^{j}|z_{t-1}=z^{i})=\frac{1}{M} \end{array}$$

or equivalently


$$\begin{array}{c} A_{ij}^{\left(unif\right)}=\frac{1}{M}\;\forall \;i,j \end{array}$$

The drift-diffusion family contains the stationary and uniform kernels as limiting cases on the discretized track. As λ→0 and σ→0, the Gaussian transition collapses to a point mass at the previous position, reproducing the stationary transition. Conversely, when σ is large relative to the track length, the Gaussian varies negligibly across destination bins; after normalization over the finite grid, the transition approaches the uniform kernel. Thus, for SWR events whose latent dynamics are dominated by one of these limiting regimes, extreme drift-diffusion parameter values can produce marginal posterior probabilities similar to those of the corresponding stationary or uniform dynamical category.

#### 4.4.2 Switching HMMs.

Individual SWR events may contain transitions between distinct dynamical regimes rather than being governed by a single transition process throughout the event [25]. To capture this structure, we used a Markov-switching hidden Markov model with a continuous latent position $z_{t}$, represented on a finite spatial grid $\left\{z^{1},...,z^{M}\right\}$, and a discrete latent regime $c_{t}$. The regime variable selects the transition kernel used for the position dynamics at each time bin.

For an event with spike-count observations *x*_1:*T*_, latent positions *z*_1:*T*_, and regimes *c*_1:*T*_, the joint distribution factorizes as


$$\begin{array}{c} p(x_{1:T},z_{1:T},c_{1:T})=\underset{\text{emissions}}{\underbrace{\prod_{t=1}^{T}p(x_{t}|z_{t})}}\underset{\text{position dynamics}}{\underbrace{p(z_{1}|c_{1})\prod_{t=2}^{T}p(z_{t}|z_{t-1},c_{t-1},c_{t})}}\underset{\text{regime switching}}{\underbrace{p(c_{1})\prod_{t=2}^{T}p(c_{t}|c_{t-1})}} \end{array}$$

In the models used here, the initial distribution over position and regime was uniform.

The emission model was conditionally independent across neurons and time bins given the latent position. Let $x_{t,i}$ be the spike count of neuron *i* in time bin *t*, and let $f_{i}(z)$ denote its place field a*t* position *z*. We used a Poisson observation model,


$$\begin{array}{c} p(x_{t}|z_{t})=\prod_{i=1}^{N}\mathrm{Poisson}\;\left(x_{t,i};\;\Delta t\;f_{i}(z_{t})\right) \end{array}$$

where Δt is the time-bin width. In the analyses reported here, Δt=10ms. The Poisson probability mass function is


$$\begin{array}{c} \mathrm{Poisson}(x;\lambda )=\frac{\lambda^{x}}{x!}\exp(-\lambda ) \end{array}$$

To allow mixtures of transition dynamics within an event, the regime chain was defined as


$$\begin{array}{c} c_{t}\in \{1,2,3\} \end{array}$$

where the three regimes corresponded to drift-diffusion, uniform, and stationary dynamics, respectively. The initial regime probabilities were


$$\begin{array}{c} p(c_{1}=i)=\pi_{i}^{\left(c\right)} \end{array}$$

with $\pi^{\left(c\right)}$ uniform in the implementation. Regime switching was governed by a Markov transition matrix


$$\begin{array}{c} p(c_{t}=j|c_{t-1}=i)=\Pi_{ij} \end{array}$$

We used a shared self-transition probability *q* for all regimes and assigned the remaining probability mass uniformly across the other regimes:


$$\begin{array}{c} \Pi =(\begin{array}{ccc} q & \frac{1-q}{2} & \frac{1-q}{2}\\ \frac{1-q}{2} & q & \frac{1-q}{2}\\ \frac{1-q}{2} & \frac{1-q}{2} & q \end{array}) \end{array}$$

Thus, *q* controls the persistence of the inferred dynamical regime, while switches away from the current regime are a priori equally likely. We fit *q* separately for each SWR event, together with the drift-diffusion parameters.

Conditional on the current regime, the latent position evolves according to the corresponding transition matrix,


$$\begin{array}{c} p(z_{t}=z^{j}|z_{t-1}=z^{i},c_{t}=k)=A_{ij}^{\left(k\right)} \end{array}$$

where *A*^(1)^, *A*^(2)^, and *A*^(3)^ denote the drift-diffusion, uniform, and stationary transition matrices defined above. Combining the regime and position transitions gives the filtering update


$$\begin{array}{c} p(c_{t}=k,z_{t}=z^{j}|x_{1:t-1})=\sum_{\ell =1}^{3}\sum_{i=1}^{M}\Pi_{\ell k}\;A_{ij}^{\left(k\right)}\;p(c_{t-1}=\ell ,z_{t-1}=z^{i}|x_{1:t-1}) \end{array}$$

followed by multiplication by the emission likelihood $p(x_{t}|z_{t}=z^{j})$ and normalization. This recursion yields the posterior over both latent position and dynamical regime for each SWR event.

**Optimization.** For each SWR event, we fit the dynamical parameters


$$\begin{array}{c} \theta =\{q,\lambda ,\sigma \} \end{array}$$

where *q* is the regime self-transition probability and λ and σ are the drift and diffusion parameters. Parameters were estimated by maximum marginal likelihood,


$$\begin{array}{c} \hat{\theta }=\text{arg}\max_{\theta }p(x_{1:T}|\theta )=\text{arg}\max_{\theta }\sum_{c_{1:T}}\sum_{z_{1:T}}p(x_{1:T},z_{1:T},c_{1:T}|\theta ) \end{array}$$

The marginal likelihood was computed with a forward pass over the joint state $\left(c_{t},z_{t}\right)$. Let


$$\begin{array}{c} \alpha_{t}(c,z)=p(x_{1:t},c_{t}=c,z_{t}=z|\theta ) \end{array}$$

denote the unnormalized forward variable. The initialization is


$$\begin{array}{c} \alpha_{1}(c,z)=p(c_{1}=c,z_{1}=z)\;p(x_{1}|z_{1}=z) \end{array}$$

with a uniform initial distribution over regimes and spatial bins. For t=2,...,T, the recursion is


$$\begin{array}{c} \alpha_{t}(k,z^{j})=p(x_{t}|z_{t}=z^{j})\sum_{\ell =1}^{C}\sum_{i=1}^{M}\alpha_{t-1}(\ell ,z^{i})\;\Pi_{\ell k}\;A_{ij}^{\left(k\right)} \end{array}$$

where *C* = 3 is the number of regimes, *M* is the number of spatial bins, $\Pi_{\ell k}=p(c_{t}=k|c_{t-1}=\ell )$, and $A_{ij}^{\left(k\right)}=p(z_{t}=z^{j}|z_{t-1}=z^{i},c_{t}=k)$. The marginal likelihood is obtained by summing the final forward variable,


$$\begin{array}{c} p(x_{1:T}|\theta )=\sum_{k=1}^{C}\sum_{j=1}^{M}\alpha_{T}(k,z^{j}) \end{array}$$

The loss minimized during fitting was the negative marginal log likelihood. Optimization used scipy.optimize.minimize with the L-BFGS-B algorithm and box constraints. In physical units, these constraints were


$$\begin{array}{c} 0.50\leq q\leq 0.99,\;-15\leq \lambda \leq 15\;m/s,\;0.1\leq \sigma \leq 1\;m/\sqrt{s} \end{array}$$

Equivalently, for the discretized one-dimensional grid used in the implementation, the bounds were applied as −750≤λ≤750 bins/s and 5≤σ≤50 bins/$\sqrt{s}$. We used the default L-BFGS-B stopping rules in SciPy, including stopping when the relative objective decrease was less than or equal to $2.2\times 10^{-9}$, when the projected-gradient $\ell_{\infty }$-norm was less than or equal to 10^–5^, or when the default iteration or function-evaluation limit of 15,000 was reached.

**Inference.** After fitting $\hat{\theta }$ for an SWR event, we computed the posterior over regimes and positions at each time bin,


$$\begin{array}{c} p(c_{t},z_{t}|x_{1:T},\hat{\theta }) \end{array}$$

The causal filtering distribution is the forward variable,


$$\begin{array}{c} p(c_{t}=k,z_{t}=z^{j}|x_{1:t},\hat{\theta })=\frac{\alpha_{t}(k,z^{j})}{\sum_{k^{\prime }=1}^{C}\sum_{j^{\prime }=1}^{M}\alpha_{t}(k^{\prime },z^{j^{\prime }})} \end{array}$$

We then applied a backward smoothing recursion to incorporate future observations. Equivalently, defining


$$\begin{array}{c} \beta_{t}(c,z)=p(x_{t+1:T}|c_{t}=c,z_{t}=z,\hat{\theta }) \end{array}$$

with terminal condition $\beta_{T}(c,z)=1$, the backward recursion is


$$\begin{array}{c} \beta_{t}(k,z^{i})=\sum_{\ell =1}^{C}\sum_{j=1}^{M}\Pi_{k\ell }\;A_{ij}^{\left(\ell \right)}\;p(x_{t+1}|z_{t+1}=z^{j})\;\beta_{t+1}(\ell ,z^{j}) \end{array}$$

for t=T−1,...,1. The smoothed joint posterior is then


$$\begin{array}{c} p(c_{t}=k,z_{t}=z^{j}|x_{1:T},\hat{\theta })=\frac{\alpha_{t}(k,z^{j})\;\beta_{t}(k,z^{j})}{\sum_{k^{\prime }=1}^{C}\sum_{j^{\prime }=1}^{M}\alpha_{t}(k^{\prime },z^{j^{\prime }})\;\beta_{t}(k^{\prime },z^{j^{\prime }})} \end{array}$$

Marginal posteriors over regimes and positions were obtained by summing the smoothed joint posterior:


$$\begin{array}{c} p(c_{t}=k|x_{1:T})=\sum_{j=1}^{M}p(c_{t}=k,z_{t}=z^{j}|x_{1:T}) \end{array}$$

$$\begin{array}{c} p(z_{t}=z^{j}|x_{1:T})=\sum_{k=1}^{C}p(c_{t}=k,z_{t}=z^{j}|x_{1:T}) \end{array}$$

These marginal distributions were used to summarize the latent trajectory and the segmentation of each SWR into dynamical regimes.

**Simulation.** The switching HMM also defines a generative model for synthetic SWR events. Given ground-truth parameters


$$\begin{array}{c} \theta_{gt}=\{q_{gt},\lambda_{gt},\sigma_{gt}\}, \end{array}$$

place fields $\{f_{i}(z){\}}_{i=1}^{N}$, and a sequence length *T*, we generated latent regimes, latent positions, and spike-count observations from the same transition and emission models used for inference.

We first initialized the latent regime and position,


$$\begin{array}{c} c_{1}^{sim}\sim p(c_{1}),\;z_{1}^{sim}\sim p(z_{1}) \end{array}$$

For each subsequent time bin t=2,...,T, the regime was drawn from the ground-truth regime-transition matrix,


$$\begin{array}{c} c_{t}^{sim}\sim p(c_{t}|c_{t-1}^{sim};q_{gt}) \end{array}$$

and the position was then drawn from the transition kernel selected by the new regime,


$$\begin{array}{c} z_{t}^{sim}\sim p\;\left(z_{t}|z_{t-1}^{sim},c_{t}^{sim};\lambda_{gt},\sigma_{gt}\right) \end{array}$$

On the finite spatial grid, this corresponds to sampling


$$\begin{array}{c} z_{t}^{sim}=z^{j}\;\text{with probability}\;A_{ij}^{\left(c_{t}^{sim}\right)} \end{array}$$

where $z_{t-1}^{sim}=z^{i}$. The drift-diffusion, uniform, and stationary transition matrices were therefore identical to those used in the fitted model. When needed for model recovery analyses, we also generated events with a prescribed regime sequence, including fixed single-regime events or events with a deterministic switch between two regimes.

Given the simulated position sequence, spike counts were sampled independently across neurons and time bins according to the Poisson emission model,


$$\begin{array}{c} x_{t,i}^{sim}\sim \mathrm{Poisson}\;\left(\Delta t\;f_{i}(z_{t}^{sim})\right),\;i=1,...,N,\;t=1,...,T \end{array}$$

Equivalently,


$$\begin{array}{c} p(x_{t}^{sim}|z_{t}^{sim})=\prod_{i=1}^{N}\mathrm{Poisson}\;\left(x_{t,i}^{sim};\;\Delta t\;f_{i}(z_{t}^{sim})\right) \end{array}$$

The standard simulations used Δt=10ms.

This procedure produced synthetic spike-count data $x_{1:T}^{sim}$ together with the ground-truth regime sequences $c_{1:T}^{sim}$ and latent trajectories $z_{1:T}^{sim}$. We then applied the same maximum-likelihood fitting and forward–backward posterior inference used for real SWR events to quantify recovery of $\theta_{gt}$ and of the underlying dynamical regime sequence.

### 4.5 Classic Bayesian decoding and analysis metrics

We applied standard Bayesian decoding as a reference analysis for decoding latent position during SWR events. Each SWR was represented as spike counts in non-overlapping 10 ms time bins. Let ${𝐱}_{t}=(x_{t,1},...,x_{t,N})$ denote the spike-count vector in time bin *t*, and let $z^{1},...,z^{M}$ denote the spa*t*ial bins. For each time bin, we computed the posterior over position using Bayes’ rule with a uniform spatial prior,


$$\begin{array}{c} p(z^{k}|{𝐱}_{t})=\frac{p({𝐱}_{t}|z^{k})}{\sum_{\ell =1}^{M}p({𝐱}_{t}|z^{\ell })} \end{array}$$

equivalent to setting $p(z^{k})=1/M$ for all spatial bins.

The likelihood assumed conditionally independent Poisson spike counts across neurons,


$$\begin{array}{c} p({𝐱}_{t}|z^{k})=\prod_{i=1}^{N}\frac{{\left(\Delta t\;f_{i}(z^{k})\right)}^{x_{t,i}}}{x_{t,i}!}\exp\;\left[-\Delta t\;f_{i}(z^{k})\right] \end{array}$$

where Δt=0.01s is the decoding-bin width and $f_{i}(z^{k})$ is the place-field firing rate of neuron *i* in spatial bin *k*. The posterior was normalized independently within each time bin so that $\sum_{k=1}^{M}p(z^{k}|{𝐱}_{t})=1$. A point estimate of decoded latent position was obtained from the maximum a posteriori (MAP) bin index,


$$\begin{array}{c} {\hat{k}}_{t}=\text{arg}\max_{1\leq k\leq M}p(z^{k}|{𝐱}_{t}) \end{array}$$

**Absolute correlation.** To quantify sequential structure, we computed the posterior-weighted Pearson correlation between time and position. Let


$$\begin{array}{c} w_{tk}=p(z^{k}|{𝐱}_{t}),\;\tau_{t}=(t-1)\Delta t,\;y_{k}=(k-1)\Delta z \end{array}$$

where Δz is the spatial bin size. The posterior-weighted means are


$$\begin{array}{c} \bar{\tau }=\frac{\sum_{t=1}^{T}\sum_{k=1}^{M}w_{tk}\tau_{t}}{\sum_{t=1}^{T}\sum_{k=1}^{M}w_{tk}},\;\bar{y}=\frac{\sum_{t=1}^{T}\sum_{k=1}^{M}w_{tk}y_{k}}{\sum_{t=1}^{T}\sum_{k=1}^{M}w_{tk}} \end{array}$$

The weighted covariance and variances are


$$\begin{array}{c} \mathrm{cov}(\tau ,y)=\frac{\sum_{t=1}^{T}\sum_{k=1}^{M}w_{tk}(\tau_{t}-\bar{\tau })(y_{k}-\bar{y})}{\sum_{t=1}^{T}\sum_{k=1}^{M}w_{tk}} \end{array}$$

$$\begin{array}{c} \mathrm{var}(\tau )=\frac{\sum_{t=1}^{T}\sum_{k=1}^{M}w_{tk}(\tau_{t}-\bar{\tau }{)}^{2}}{\sum_{t=1}^{T}\sum_{k=1}^{M}w_{tk}},\;\mathrm{var}(y)=\frac{\sum_{t=1}^{T}\sum_{k=1}^{M}w_{tk}(y_{k}-\bar{y}{)}^{2}}{\sum_{t=1}^{T}\sum_{k=1}^{M}w_{tk}} \end{array}$$

The weighted correlation is


$$\begin{array}{c} \rho =\frac{\mathrm{cov}(\tau ,y)}{\sqrt{\mathrm{var}(\tau )\mathrm{var}(y)}} \end{array}$$

We report |ρ|, where values near 1 indicate a monotonic sequential trajectory and values near 0 indicate little monotonic relationship between decoded position and time.

**Replay speed.** Replay speed was estimated from the MAP trajectory. We fit a linear regression of the decoded latent trajectory,


$$\begin{array}{c} {\hat{k}}_{t}=\beta_{0}+\beta_{1}t+\varepsilon_{t} \end{array}$$

where ${\hat{k}}_{t}$ is the MAP spatial-bin index at time *t*. The replay speed was defined as the absolute fi*tt*ed slope converted to physical units,


$$\begin{array}{c} v_{replay}=|\beta_{1}|\;\frac{\Delta z}{\Delta t} \end{array}$$

Thus, the reported speed has units of distance per second.

**Maximum jump distance.** We quantified the largest discontinuity in the decoded MAP trajectory by computing adjacent-bin jumps,


$$\begin{array}{c} \Delta_{t}=\Delta z\;|{\hat{k}}_{t}-{\hat{k}}_{t-1}|,\;t=2,...,T \end{array}$$

and taking


$$\begin{array}{c} D_{\max}=\max_{2\leq t\leq T}\Delta_{t} \end{array}$$

Large values indicate abrupt spatial jumps in the decoded trajectory, whereas small values indicate more spatially continuous decoded sequences.

### 4.6 Additional figure details

#### 4.6.1 Model recovery experiments.

We performed simulation analyses to assess recovery of drift-diffusion parameters and recovery of the latent dynamical regime sequence in the switching HMM. For Fig 3 recovery analyses, we simulated a population of 120 place cells on a one-dimensional 2 m linear track discretized into 100 spatial bins of width Δz=0.02m. Place fields were Gaussian bumps,


$$\begin{array}{c} f_{i}(z)=20\;Hz\;\exp\;\left[-\frac{(z-\mu_{i}{)}^{2}}{2(0.1\;m{)}^{2}}\right], \end{array}$$

where each preferred position $\mu_{i}$ was sampled uniformly from the spatial bins. Spike counts were generated in Δt=10ms bins.

**Drift-diffusion parameter recovery.** To test whether the model could recover drift-diffusion parameters, we generated single-regime drift-diffusion events under a partial grid of ground-truth drift and diffusion values. In physical units, the grid consisted of


$$\begin{array}{c} \lambda_{gt}\in \{0,2,4,6,8\}\;m/s\;\text{at}\;\sigma_{gt}\in \{0.2,0.4\}\;m/\sqrt{s}, \end{array}$$

and


$$\begin{array}{c} \sigma_{gt}\in \{0.1,0.2,0.3,0.4,0.5\}\;m/\sqrt{s}\;\text{at}\;\lambda_{gt}\in \{0,2\}\;m/s. \end{array}$$

Equivalently, in model units these corresponded to $\lambda_{gt}\in \{0,100,200,300,400\}$ bins/s and $\sigma_{gt}\in \{5,10,15,20,25\}$ bins/$\sqrt{s}$. For each grid condition, we simulated 100 independent SWR events. Each event contained 15 time bins, corresponding to 150 ms of simulated activity. The latent position evolved according to the drift-diffusion transition kernel with the specified ground-truth parameters. Conditional on the latent position sequence, Poisson spike counts were sampled from the synthetic place-cell population. The full inference and maximum-likelihood fitting pipeline was then applied to each simulated event, yielding estimates $\hat{\lambda }$ and $\hat{\sigma }$. For plotting, estimates were summarized across the 100 repeats for each ground-truth condition.

**Switching dynamics recovery.** To test recovery of regime structure, we simulated two-segment events using the three candidate dynamics: drift-diffusion, uniform, and stationary. We considered every ordered pair of distinct regimes for the first and second segments. When either segment was drift-diffusion, we simulated that condition across the same partial $\left(\lambda_{gt},\sigma_{gt}\right)$ grid used for the single-regime drift-diffusion model recovery analysis. When neither segment was drift-diffusion, placeholder drift-diffusion parameters were included but not optimized.

For each ordered regime-pair and parameter condition, we generated 100 independent SWR events. Each event contained 30 time bins, corresponding to 300 ms total duration, with the leading 15 bins generated from the first regime and the latter 15 bins generated from the second regime. Latent positions were sampled from the transition matrix associated with the current ground-truth regime: drift-diffusion used the specified $\left(\lambda_{gt},\sigma_{gt}\right)$, stationary used the identity transition, and uniform used a spatially uniform transition. Poisson spike counts were then sampled from the synthetic place-cell population along the latent trajectory.

We applied the same fitting and posterior-inference pipeline to these simulated events. For drift-diffusion parameter recovery in the full switching model, we compared $\hat{\lambda }$ and $\hat{\sigma }$ with the ground-truth values for conditions that contained at least one drift-diffusion segment. For regime recovery, we computed the marginal posterior over dynamical regimes at each time bin and assigned a predicted label using a posterior threshold of 0.7. Time bins with posterior probability at least 0.7 for a single regime were assigned to that regime. Bins whose combined stationary and drift-diffusion posterior exceeded 0.7 were counted as stationary, whereas other bins were labeled unclassified. We then constructed confusion matrices comparing predicted and ground-truth regimes, separately for uniform-stationary switches and for conditions containing drift-diffusion segments with $\lambda_{gt}=0$, 2, 4, or at least 6m/s.

#### 4.6.2 Speed measures.

We computed three speed measures for Fig 6: per-lap running speed, instantaneous behavioral speed, and replay speed.

**Per-lap running speed.** Running laps were identified from the linearized position trace for each running epoch. For each epoch, we defined the track midpoint as


$$\begin{array}{c} m=\frac{\min x(t)+\max x(t)}{2} \end{array}$$

and defined lower and upper lap endpoints as m−25cm and m+25cm, respectively. Upward laps were identified by upward crossings of the midpoint. For each upward crossing, the lap start was the most recent sample at or below the lower endpoint and the lap end was the first subsequent sample at or above the upper endpoint. Downward laps were defined analogously, using the most recent sample at or above the upper endpoint and the first subsequent sample at or below the lower endpoint. For each extracted lap, we fit a linear regression of linearized position on time,


$$\begin{array}{c} x(t)=a+vt+\epsilon (t) \end{array}$$

and defined the per-lap running speed as |*v*|. Slopes were divided by 100 to report speed in m/s as the position trace was recorded in centimeters.

**Instantaneous behavioral speed.** Instantaneous behavioral speed was computed from the two-dimensional position tracking data (before position linearization). Speed was estimated with a centered finite difference,


$$\begin{array}{c} s_{i}=\frac{\sqrt{[x_{i+1}-x_{i-1}{]}^{2}+[y_{i+1}-y_{i-1}{]}^{2}}}{t_{i+1}-t_{i-1}} \end{array}$$

with forward and backward differences used for the first and last samples, respectively. Speeds were concatenated across running epochs and converted from cm/s to m/s.

**Replay speed.** Replay speed was computed for SWRs classified as drift-diffusion events. For each such event, we used the fitted drift parameter from the switching HMM. The fitted parameter $\hat{\lambda }$ is stored in units of spatial bins per second, so replay speed was defined as


$$\begin{array}{c} v_{replay}=|\hat{\lambda }|\;\Delta z \end{array}$$

where Δz=0.02m is the spatial bin width. Replay speeds were therefore reported in m/s and summarized separately by behavioral state where applicable.

#### 4.6.3 Shuffle analysis.

Null distributions were generated using three shuffle procedures, each repeated for 120 times. For the time-bin shuffle, the time bins of the spike-count raster were randomly permuted within each SWR using a single permutation shared across neurons. This preserved the population activity vector in each time bin and the total spike count of each neuron while disrupting temporal order. For the neuron-identity shuffle, all neuron columns included in the analysis for a recording session were randomly permuted within each SWR, including neurons that were silent during that event. A single permutation was applied across all time bins of the SWR, and an independent permutation was drawn for each SWR and shuffle repeat. For the place-field rotation shuffle, the spike rasters were left unchanged and each neuron’s place field was independently circularly shifted by a uniformly sampled integer number of spatial bins. One shuffled place-field ensemble was generated for each repeat and used for all SWRs from that recording session. Each shuffled realization was fitted and analyzed using the same pipeline as the real data.

## Supporting information

S1 Text Parameters in the drift-diffusion framework. Compare classical Bayesian replay metrics with the drift and diffusion parameters of the switching-HMM framework.
(PDF)

S2 Text Residual analysis. Compare position estimates from the switching HMM and a memory-less Bayesian decoder during awake and post-sleep SWRs.
(PDF)

S3 Text Additional event-detection analysis. Test the robustness of the inferred dynamics using population-burst events detected from multiunit activity.
(PDF)

S4 Text Shuffle-control discussion. Compare the time-bin, neuron-identity, and place-field rotation shuffle controls.
(PDF)

S5 Text Bidirectional place-field discussion. Discuss the use of place fields pooled across running directions.
(PDF)

S6 Text Posterior-probability derivation. Provide a derivation of the forward filtering and backward smoothing recursions, data likelihood, and marginal posterior probabilities used for analysis.
(PDF)

S7 Text Drift-diffusion estimation bias. Derive the finite-sample downward bias in diffusion estimates for short events and also extends the analysis from a single-regime model to the switching HMM.
(PDF)

S1 FigRelationship to classical Bayesian replay metrics.A. Classical two-step replay analysis pipeline. A spike-raster matrix is decoded into a position posterior $p(z_{t}|x_{t})\propto p(x_{t}|z_{t})p(z_{t})$, using Bayes’ rule with each place field. The decoded trajectory is then summarized by the maximum a posteriori estimate ${\hat{z}}_{t}=\text{arg}\max_{z}p(z_{t}|x_{t})$. Red annotations indicate commonly used replay metrics derived from either the spikes, posterior, or decoded trajectory: the temporal firing template relative to the animal’s running firing sequence; the absolute weighted time-position correlation $\left|\rho_{t,z}\right|$ under the posterior; the replay speed, defined as the absolute slope of a linear fit ${\hat{z}}_{t}=\beta_{0}+\beta_{1}t$; and the maximum jump distance $D_{\max}=\max_{t}|{\hat{z}}_{t}-{\hat{z}}_{t-1}|$. B. Dependence of the three classical Bayesian replay metrics on the drift-diffusion parameters. The top row varies drift λ from 0 to 10 m/s with σ fixed at 0.4 m/$\sqrt{s}$. The bottom row varies diffusion σ from 0.2 to 8.0 m/$\sqrt{s}$ with λ fixed at 2.0 m/s. Boxes show the median and interquartile range, whiskers extend to 1.5× the interquartile range, and points denote outliers.(TIF)

S2 Fig Residuals between switching-HMM and Bayesian decoded positions during sharp-wave ripples. Quantiles of the absolute position residual between the maximum a posteriori (MAP) estimate from the switching-HMM position marginal and the MAP estimate from the memory-less Bayesian decoder. Position residuals of SWR time bins are grouped by brain state and assigned dynamics class. Rows, top to bottom, show awake and post-sleep SWRs. Columns, left to right, show drift-diffusion, stationary, and uniform dynamics. Solid colored curves show the residual distributions in SWRs. Dashed light gray curves show a control baseline in which Bayesian MAP positions were permuted across time bins before recomputing the residual.
(TIF)

S3 FigComposition of inferred spatiotemporal dynamics in events detected from multiunit activity alone.Population-burst events were detected independently of the LFP by summing spikes across neurons in 10 ms bins, smoothing the population activity with a Gaussian kernel (σ=12.5 ms), and identifying excursions exceeding 3 SD above the session mean, with event boundaries extended to the surrounding mean crossings. Events lasting 50–2000 ms were retained, and events during run epochs were restricted to mean speeds below 5 cm/s. The same switching-HMM and dynamics-classification procedure used for the manuscript events was applied to these pure-MUA events. For event-level summaries, a dynamic class was counted as present only when it persisted for at least two consecutive 10 ms bins. A,C,E. UpSet-plot summaries of dynamic-class combinations in pre-sleep, awake, and post-sleep events, respectively. Filled dots mark classes present in each combination, connected filled dots indicate co-occurring classes within the same event, and open dots mark absent classes. The left horizontal bars show the percentage of events containing each class. B,D,F. Fractions of event time bins assigned to each dynamic class for the corresponding epochs. Only class combinations occurring in more than 1% of events are shown. Events were pooled across N = 9 recording sessions (pre-sleep, n = 11,409; awake, n = 5,437; post-sleep, n = 22,089).(TIF)

S4 Fig Additional examples of inferred spatiotemporal dynamics in real SWR events. Rows show drift-diffusion events, stationary events, uniform events, and events containing “No Class” time bins; columns show individual examples. For each event, panels from top to bottom show the spike raster, memory-less Bayesian position posterior, posterior probabilities of the three dynamic regimes, and switching-HMM position posterior. In the dynamic-probability panels, green, orange, and gray curves denote drift-diffusion, stationary, and uniform probabilities, respectively; background shading in the same colors indicates the assigned regime, and unshaded intervals indicate “No Class.” In the position-posterior heat maps, brighter colors indicate higher posterior probability.
(TIF)

S5 Fig Replay-speed distributions are inconsistent with a pure-diffusion null. A–B. Histograms of replay speed for drift-diffusion events in awake and post-sleep periods. Drift-diffusion events were identified from the marginal posterior of dynamics using a posterior threshold of 0.7 sustained for at least five consecutive 10 ms bins. Replay speed was estimated from the fitted drift rate. C. Speed distribution under a zero-drift diffusion model. For every drift-diffusion event in A–B, a pure-diffusion model was fit by maximum likelihood to estimate the diffusion parameter. From the fitted pure-diffusion model, 10 trajectories with the same duration as the corresponding event were simulated. The speed of each simulated trajectory was defined as the absolute slope from a linear regression of position against time. Dashed red lines mark the mean of each distribution: 4.677 m/s (awake events), 4.002 m/s (post-sleep events) and 1.488 m/s (pure-diffusion trajectories). Dashed dark gray lines in A–B mark the pure-diffusion mean. Brackets indicate one-sided Welch two-sample tests comparing the mean replay speed in awake or post-sleep against the mean speed of simulated pure-diffusion trajectories, with the alternative hypothesis that replay speed is greater than the pure-diffusion speed. Asterisks denote statistical significance (***, *p* < 0.001; awake vs. pure diffusion, *p* < 0.001; post-sleep vs. pure diffusion, *p* < 0.001).
(TIF)

S6 Fig Decoded and tracked positions for stationary-state time bins in awake SWRs. Histograms summarize all time bins labeled as stationary by the model within awake SWR events (total duration T = 88.99 s across 9 sessions). Each histogram is normalized to the proportion of stationary time. A. SWR-decoded latent position. B. Tracked animal position on the linear track at the same time bins. C. Absolute difference between the decoded and tracked positions.
(TIF)

S7 Fig Decoded position distributions for stationary dynamics in real data and shuffles from one session of rat Janni. Histograms show the decoded positions of time bins classified as stationary, pooled across awake and post-sleep SWRs. For each stationary bin, decoded position was defined as the maximum of the position posterior after marginalizing over dynamics states. Bar heights give the proportion of stationary bins in each position interval along the 1.8 m track. The dark gray histogram at upper left shows the distribution for the real data. Gray histograms show five independent realizations of each control: time bins were permuted within each SWR (top row), neuron identities were permuted within each SWR (middle row), or each neuron’s place field was independently circularly shifted along the track (bottom row). The model was fit and posterior inference was performed separately for each shuffled realization.
(TIF)

S8 Fig Decoded position distributions for stationary dynamics in real data and shuffles from one session of rat Ettin. Same analysis and conventions as in S7 Fig.
(TIF)

S9 FigMSD-Δt and RMSD-Δt scaling are distinct for stationary, uniform, and all SWR events.Each panel repeats the displacement scaling analysis (Fig 7) for the indicated SWR subset. Within each panel, the three plots show, from left to right, mean squared displacement (MSD) as a function of time lag Δt, $\log_{10}(\text{RMSD})$ as a function of $\log_{10}(\Delta t)$, and the fitted log-log RMSD slope for each included session. Data are shown in purple and Brownian-motion simulations in gray. Line plots show mean ± s.d. across sessions. Dashed horizontal lines in the slope plots mark the session mean. A. Awake SWRs with pure stationary dynamics (*n* = 6 sessions; mean data slope, 0.27; mean simulation slope, 0.49). B. All awake SWRs (*n* = 9 sessions; mean data slope, 0.82; mean simulation slope, 0.49). C. Post-sleep SWRs with pure stationary dynamics (*n* = 8 sessions; mean data slope, 0.21; mean simulation slope, 0.49). D. Post-sleep SWRs with pure uniform dynamics (*n* = 8 sessions; mean data slope, 0.39; mean simulation slope, 0.49). The data point at Δt=0 is omitted in the MSD plot because the zero-lag estimate is degenerate for the uniform-dynamics subset. E. All post-sleep SWRs (*n* = 8 sessions; mean data slope, 0.57; mean simulation slope, 0.49). Sessions with fewer than 10 qualifying SWRs were excluded from the corresponding panel. The awake-uniform panel is omitted because no awake session met this inclusion criterion.(TIF)

S10 Fig Composition of inferred spatiotemporal dynamics in pre-sleep SWRs. The marginal posterior of dynamics for each pre-sleep SWR was assigned to a dynamic class following the procedure described in Fig 2. For event-level summaries, a dynamic class was counted as present in an SWR only when it persisted for at least two consecutive 10 ms bins. A. UpSet-plot [50] summary of dynamic-class combinations in pre-sleep SWRs, using the same conventions as in Fig 4. The top bar plot shows the percentage of SWRs exhibiting each class combination, as indicated by the dot matrix below. The left horizontal bar plot shows the percentage of SWRs containing each individual class. B. Fraction of pre-sleep SWR time bins assigned to each dynamic class. Only class combinations occurring in more than 1% of pre-sleep SWRs are shown. Compared with post-sleep SWRs, pre-sleep SWRs showed less time in drift-diffusion dynamics and more time in uniform.
(TIF)

S11 Fig Drift-diffusion duration in shuffled SWRs across brain states. For each shuffle repeat, SWRs were assigned dynamic-class labels from the marginal posterior of dynamics, and drift-diffusion duration was defined as the total time assigned to the drift-diffusion regime. The y-axis shows the proportion of shuffled SWRs whose drift-diffusion duration exceeded each threshold on the x-axis. Solid colored curves show the mean proportion across 120 shuffle repeats for pre-sleep (blue), awake (green), and post-sleep (orange) SWRs. Violins show the corresponding distribution across shuffle repeats. Columns show the three shuffle controls used in Fig 8: time-bin shuffle, neuron-identity shuffle, and place-field rotation shuffle.
(TIF)

S12 Fig Place fields from an example session in rat Janni. Left, bidirectional place fields estimated by pooling spikes and occupancy across both running directions. Middle and right, direction-specific place fields estimated from left-to-right and right-to-left traversals, respectively. Each row shows one retained excitatory neuron, with firing rates normalized to the peak of that neuron’s place field. Neurons are ordered by the peak position of the bidirectional place field, and the same ordering is used in all panels.
(TIF)

S13 Fig Bayesian decoding of running position using bidirectional place fields. Heatmaps show the posterior probability of the animal’s position along the linear track, estimated from run-epoch place-cell activity with a memory-less Bayesian decoder. Brighter colors indicate higher posterior probability, and the white trace shows the tracked position. Panels show consecutive time segments from the same session as in S12 Fig.
(TIF)

S14 Fig Place fields from an example session in rat Harpy. Plotting conventions follow S12 Fig.
(TIF)

S15 Fig Bayesian decoding of running position using bidirectional place fields. Plotting conventions follow S13 Fig. Same session as in S14 Fig.
(TIF)
