# Supplementary material for: Drift-diffusion dynamics of hippocampal replay
Source: PLoS Comput Biol. 2026 Sep 22;22(9):e1014761. doi: 10.1371/journal.pcbi.1014761 (PMC13614660; doi:10.1371/journal.pcbi.1014761)
Supplement: Supplementary file 1 [file pcbi.1014761.s001.pdf]

## S1 Text. Parameters in the drift-diffusion framework

Previous methods first infer the posterior probability over space using the place fields inferred during running [34], then compute three metrics for interpretation (summarized in [46]): (i) (absolute) correlation between time and decoded trajectory; (ii) replay speed inferred from the slope of decoded trajectory; and (iii) maximum jump distance (S1A Fig). In contrast, our modeling framework uses a generative approach and describes a replay event using the drift and diffusion parameters, which unambiguously characterize the speed and the sequentiality (or quality) of the replay respectively. We next aim to determine the relationship between the key parameters in prior methods and those in ours.

We generate simulated neural population recording data with different combinations of drift and diffusion parameters, and then apply the traditional analysis method based on Bayesian decoding to extract the three metrics mentioned above, i.e., absolute correlation, speed of replay, maximum jump distance (S1A Fig). S1B Fig shows the results when fixing the diffusion parameter while allowing the drift parameter to vary, while S1C Fig shows the results when fixing the drift parameter while allowing the diffusion parameter to vary. These results suggest that the absolute correlation is positively correlated with drift and negatively correlated with diffusion. For the speed inferred from the Bayesian approach, it is positively correlated with both the drift and diffusion parameters. Thus, the speed can be overestimated when the diffusion parameter is large. Furthermore, the maximum jump distance [46] mostly reflects the magnitude of the diffusion parameter, i.e., large diffusion leads to a bigger maximum jump distance, yet it also increases systematically with the drift. This suggests that maximum jump distance reflects a combination of drift and diffusion, in particular when the diffusion parameter is small. Together, these results suggest that, while the three key metrics used in prior studies may capture some structures in the replay dynamics as conceived when they were developed, each of them reflects the combined effects of the speed and the quality of a candidate replay event. Thus, practically they may not allow for an unambiguous interpretation of the replay dynamics. In contrast, our approach clearly distinguishes the speed and quality of a replay event using separate drift and diffusion parameters, and it allows one to dissect the contribution of the two in a principled single-step inference framework.
