# Supplementary material for: Drift-diffusion dynamics of hippocampal replay
Source: PLoS Comput Biol. 2026 Sep 22;22(9):e1014761. doi: 10.1371/journal.pcbi.1014761 (PMC13614660; doi:10.1371/journal.pcbi.1014761)
Supplement: Supplementary file 2 [file pcbi.1014761.s002.pdf]

S2 Text. Residual analysis

As a complementary diagnostic of real-data decoding, we compared the maximum a posteriori (MAP) position estimate from the switching HMM with the estimate from a memory-less Bayesian decoder applied independently to each time bin (S2 Fig). For the three dynamics considered in the model, the residual error is much lower than the shuffle control and remains small. In awake SWRs, the 75th percentiles of the absolute residual were 12 cm for drift-diffusion bins, 10 cm for stationary bins, and 10 cm for uniform bins on a 180 cm track. In post-sleep SWRs, the corresponding values were 20, 32, and 2 cm. These values were far below the time-permuted controls, whose 75th percentiles were 116, 138, and 100 cm in awake SWRs and 112, 128, and 110 cm in post-sleep SWRs. This analysis indicates that the switching-HMM decoding remains close to the position information supported directly by the instantaneous spike activity. We note that our model includes switching dynamics to account for temporal structure in the position posterior. Therefore, we expect some difference between our decoding and memory-less Bayesian decoding, and the residual analysis is intended to verify that this difference is modest, rather than a large mismatch between the model and the spike-based position evidence.
