# Supplementary material for: Drift-diffusion dynamics of hippocampal replay
Source: PLoS Comput Biol. 2026 Sep 22;22(9):e1014761. doi: 10.1371/journal.pcbi.1014761 (PMC13614660; doi:10.1371/journal.pcbi.1014761)
Supplement: Supplementary file 3 [file pcbi.1014761.s003.pdf]

S3 Text. Additional event-detection analysis

To examine whether the inferred composition of dynamics depends on the event detection criterion, we performed a complementary analysis using population-burst events detected from MUA alone, without requiring ripple-band activity in the LFP (S3 Fig). We summed spikes across neurons in non-overlapping 10 ms bins, smoothed the resulting population activity with a Gaussian kernel (σ = 12.5 ms), and z-scored the smoothed activity within each recording session. MUA events were defined as excursions exceeding 3 SD above the session mean, with event boundaries extended to the surrounding mean crossings. We retained events lasting 50–2000 ms and, for events occurring during run epochs, required a mean running speed below 5 cm/s.

This procedure identified 38,935 pure-MUA events across nine recording sessions, compared with the 23,272 events considered in the primary manuscript analysis. We applied the same switching-HMM fitting and dynamics-classification pipeline to all pure-MUA events. Qualitatively, stationary dynamics occupied the largest fraction of time in every epoch, and awake events exhibited more drift-diffusion dynamics than pre- or post-sleep events. At the event level, the fractions of pure-MUA events containing at least one drift-diffusion segment were 10.78%, 38.27%, and 16.46% for pre-sleep, awake, and post-sleep, respectively, compared with 9.82%, 49.72%, and 18.05% for the primary manuscript events. At the time-bin level, drift-diffusion accounted for 5.81%, 28.12%, and 10.27% of pure-MUA event time, compared with 5.83%, 39.53%, and 13.46% for the primary manuscript events.

One quantitative difference was the lower fraction of drift-diffusion dynamics in the pure-MUA analysis during awake rest, with a smaller reduction during post-sleep and nearly unchanged results during pre-sleep. This suggests that the broader MUA-only criterion admits additional population bursts with weaker sequential spatial structure. Nevertheless, the qualitative relationships across brain states and the overall composition of dynamics remained consistent with the primary manuscript results.

Overall, the pure-MUA analysis provides a complementary robustness analysis showing that the qualitative results persist for events detected independently of ripple-band LFP activity.
