# Supplementary material for: Drift-diffusion dynamics of hippocampal replay
Source: PLoS Comput Biol. 2026 Sep 22;22(9):e1014761. doi: 10.1371/journal.pcbi.1014761 (PMC13614660; doi:10.1371/journal.pcbi.1014761)
Supplement: Supplementary file 4 [file pcbi.1014761.s004.pdf]

S4 Text. Shuffle-control discussion

When analyzing pre-run SWRs across shuffle controls, we observed a specific discrepancy for the time-bin shuffle (Figs 8 and S11). First, the fraction of SWRs exceeding each drift-diffusion duration threshold for time-bin shuffled data was consistently lower than that for the other two controls, whereas the neuron-identity and place-field rotation shuffles yielded similar null fractions. Second, the null fractions from time-bin shuffle did not align across pre- and post-run data. At every threshold, a larger fraction of post-run shuffled events exceeded the criterion than pre-run shuffled events did. This is in contrast to the overlapping pre/post null fractions produced by the neuron-identity and place-field rotation shuffles.

Note that a comparable divergence between time-bin and other shuffles was also reported by Takigawa et al. [47]. The reason is possibly that only the time-bin shuffle preserves spike–position correlations [51, 55]. But this interpretation is provisional, and the mechanism requires further investigation.
