# Supplementary material for: Drift-diffusion dynamics of hippocampal replay
Source: PLoS Comput Biol. 2026 Sep 22;22(9):e1014761. doi: 10.1371/journal.pcbi.1014761 (PMC13614660; doi:10.1371/journal.pcbi.1014761)
Supplement: Supplementary file 5 [file pcbi.1014761.s005.pdf]

S5 Text. Bidirectional place-field discussion

On a linear track, place cells may exhibit direction-dependent place fields. In our analyses, we estimate place fields of each neuron by pooling spikes and occupancy from both running directions. For clarity, we refer to these pooled estimates as bidirectional place fields throughout and use them in the main analyses. We chose to combine the data from the two directions for the following reasons. First, the peaks of individual place fields are highly consistent across directions (S12 and S14 Figs). Second, using the bidirectional fields, the animal's position during run periods can be decoded accurately (S13 and S15 Figs). Therefore, if replay recapitulates place cell activity during running and forms coherent trajectories, the bidirectional place fields should be appropriate for decoding the content of the replay events. Finally, our choice matches the preprocessing in the original report on this dataset [33], which did not split place fields by running directions.
