# Supplementary material for: Drift-diffusion dynamics of hippocampal replay
Source: PLoS Comput Biol. 2026 Sep 22;22(9):e1014761. doi: 10.1371/journal.pcbi.1014761 (PMC13614660; doi:10.1371/journal.pcbi.1014761)
Supplement: Supplementary file 6 [file pcbi.1014761.s006.pdf]

S6 Text. Posterior-probability derivation

We consider the switching hidden Markov model used in the main text. At each time bin t, the latent state consists of a discrete dynamical regime $c_t \in \{1, \ldots, C\}$ and a discretized position $z_t \in \{z^1, \ldots, z^M\}$. The observation x_t is the vector of spike counts in that time bin. The emission likelihood depends on position but not directly on the dynamical regime,

$$L_t(j) \equiv p(x_t \mid z_t = z^j)$$

Let

$$\Pi_{\ell k} = p(c_t = k \mid c_{t-1} = \ell)$$

denote the regime-transition matrix, and let

$$A_{ij}^{(\ell,k)} = p(z_t = z^j \mid z_{t-1} = z^i, c_{t-1} = \ell, c_t = k)$$

denote the position-transition matrix associated with a transition from regime ℓ to regime k.

The joint transition for the pair (c_t, z_t) factorizes as

$$p(c_t = k, z_t = z^j \mid c_{t-1} = \ell, z_{t-1} = z^i) = \Pi_{\ell k}\, A_{ij}^{(\ell,k)}$$

Forward filtering. The forward filter is the causal posterior

$$F_t(k, j) \equiv p(c_t = k, z_t = z^j \mid x_{1:t})$$

Let $\mu_1(k, j) = p(c_1 = k, z_1 = z^j)$ be the joint initial distribution over regime and position. This distribution is assumed to be uniform over all CM pairs. For the first time bin,

$$\widetilde{F}_1(k, j) = \mu_1(k, j)\, L_1(j), \qquad s_1 = \sum_{k=1}^{C} \sum_{j=1}^{M} \widetilde{F}_1(k, j)$$

and the normalized filter is

$$F_1(k, j) = \frac{\widetilde{F}_1(k, j)}{s_1}$$

For $t = 2, \ldots, T$, the one-step predictive distribution is obtained by marginalizing over the previous regime and position,

$$R_t(k, j) = \sum_{\ell=1}^{C} \sum_{i=1}^{M} \Pi_{\ell k}\, A_{ij}^{(\ell,k)}\, F_{t-1}(\ell, i)$$

Bayes' rule then gives the unnormalized update

$$\widetilde{F}_t(k, j) = L_t(j)\, R_t(k, j)$$

with normalizing constant

$$s_t = \sum_{k=1}^{C} \sum_{j=1}^{M} \widetilde{F}_t(k, j) = p(x_t \mid x_{1:t-1})$$

and normalized causal posterior

$$F_t(k,j) = \frac{\widetilde{F}_t(k,j)}{s_t}$$

Thus the data likelihood is accumulated as

$$p(x_{1:T}) = \prod_{t=1}^{T} s_t, \qquad \log p(x_{1:T}) = \sum_{t=1}^{T} \log s_t$$

This is the quantity returned by the implementation as the data log likelihood, while F_t is returned as the causal posterior.

Backward smoothing. The standard backward message is

$$B_t(k,j) \equiv p(x_{t+1:T} \mid c_t = k, z_t = z^j)$$

with terminal condition $B_T(k,j) = 1$. Marginalizing over the next latent pair gives the recursion

$$B_t(\ell,i) = \sum_{k=1}^{C} \sum_{j=1}^{M} \Pi_{\ell k}\, A_{ij}^{(\ell,k)}\, L_{t+1}(j)\, B_{t+1}(k,j)$$

for $t = T-1, \ldots, 1$. The smoothed posterior is therefore

$$\Gamma_t(k,j) \equiv p(c_t = k, z_t = z^j \mid x_{1:T}) = \frac{F_t(k,j)\, B_t(k,j)}{\sum_{k'=1}^{C} \sum_{j'=1}^{M} F_t(k',j')\, B_t(k',j')}$$

The implementation computes the same smoothed posterior using a normalized backward update rather than explicitly storing the unnormalized B_t messages. Define the prediction of the next latent pair from the causal filter at time t,

$$P_{t+1|t}(k,j) = \sum_{\ell=1}^{C} \sum_{i=1}^{M} \Pi_{\ell k}\, A_{ij}^{(\ell,k)}\, F_t(\ell,i)$$

Initialize the smoothed posterior at the final time by

$$\Gamma_T(k,j) = F_T(k,j)$$

For $t = T-1, \ldots, 1$, form the ratio

$$Q_{t+1}(k,j) = \frac{\Gamma_{t+1}(k,j)}{P_{t+1|t}(k,j)}$$

with a small numerical offset added in the implementation to avoid division by zero. The backward weight for the current latent pair is

$$W_t(\ell,i) = \sum_{k=1}^{C} \sum_{j=1}^{M} \Pi_{\ell k}\, A_{ij}^{(\ell,k)}\, Q_{t+1}(k,j)$$

The smoothed posterior is then updated by

$$\Gamma_t(\ell,i) = \frac{F_t(\ell,i)\, W_t(\ell,i)}{\sum_{\ell'=1}^{C} \sum_{i'=1}^{M} F_t(\ell',i')\, W_t(\ell',i')}$$

This recursion is the posterior returned by the implementation.

Marginal posteriors. The posterior probability of each dynamical regime is obtained by summing the smoothed joint posterior over positions,

$$p(c_t = k \mid x_{1:T}) = \sum_{j=1}^{M} \Gamma_t(k, j)$$

and the posterior probability of position is obtained by summing over regimes,

$$p(z_t = z^j \mid x_{1:T}) = \sum_{k=1}^{C} \Gamma_t(k, j)$$

These two marginals are used to summarize the inferred dynamical regime sequence and the latent spatial trajectory, respectively.
