# Supplementary material for: Drift-diffusion dynamics of hippocampal replay
Source: PLoS Comput Biol. 2026 Sep 22;22(9):e1014761. doi: 10.1371/journal.pcbi.1014761 (PMC13614660; doi:10.1371/journal.pcbi.1014761)
Supplement: Supplementary file 7 [file pcbi.1014761.s007.pdf]

S7 Text. Drift-diffusion estimation bias

We consider why estimates of the diffusion parameter can be biased downward in short replay events. For clarity, we first analyze a single-regime drift-diffusion HMM and then extend the argument to the switching HMM used in the main analyses. Let the latent position be represented on a one-dimensional grid $\{z^1, \ldots, z^M\}$, with time-bin width Δt. Ignoring boundary effects for the moment, the drift-diffusion transition kernel is

$$K_{ij}(\lambda, \sigma) \propto \mathcal{N}\big(z^j;\, z^i + \lambda \Delta t,\, \sigma^2 \Delta t\big)$$

where λ is the drift parameter and σ is the diffusion scale.

Let $B_j(x_t) = p(x_t \mid z_t = z^j)$ denote the emission likelihood from the place-field model. The observed-data log likelihood is

$$\ell(\lambda, \sigma) = \log \sum_{z_{1:T}} p(x_{1:T}, z_{1:T} \mid \lambda, \sigma)$$

and is evaluated with the forward algorithm. For notational convenience, let

$$\xi_t(i, j) = p(z_{t-1} = z^i, z_t = z^j \mid x_{1:T}, \lambda, \sigma), \qquad t = 2, \ldots, T$$

be the smoothed pairwise posterior over adjacent latent positions.

Score equations. By Fisher's identity,

$$\frac{\partial \ell}{\partial \theta} = \mathbb{E}\left[\frac{\partial}{\partial \theta} \log p(x_{1:T}, z_{1:T} \mid \theta) \middle| x_{1:T}, \theta\right], \qquad \theta = (\lambda, \sigma)$$

Only the transition terms depend on λ and σ. Writing

$$d_{ij} = z^j - z^i$$

the Gaussian approximation gives

$$\frac{\partial}{\partial \lambda} \log K_{ij}(\lambda, \sigma) = \frac{d_{ij} - \lambda \Delta t}{\sigma^2}$$

The stationary point for λ therefore satisfies

$$\hat{\lambda} = \frac{\sum_{t=2}^{T} \sum_{i,j} \xi_t(i, j) d_{ij}}{\Delta t \sum_{t=2}^{T} \sum_{i,j} \xi_t(i, j)}$$

Because $\sum_{i,j} \xi_t(i, j) = 1$, this is the posterior-weighted mean displacement divided by Δt.

Similarly, differentiating with respect to σ^2 yields

$$\frac{\partial}{\partial \sigma^2} \log K_{ij}(\lambda, \sigma) = -\frac{1}{2\sigma^2} + \frac{(d_{ij} - \lambda \Delta t)^2}{2\sigma^4 \Delta t}$$

The corresponding stationary point is

$$\hat{\sigma}^2 = \frac{\sum_{t=2}^{T} \sum_{i,j} \xi_t(i, j) \left(d_{ij} - \hat{\lambda} \Delta t\right)^2}{\Delta t \sum_{t=2}^{T} \sum_{i,j} \xi_t(i, j)}$$

These equations show that the diffusion estimate is a posterior-weighted residual variance of the inferred displacement increments.

Bias in the ideal known-state limit. To isolate the finite-sample effect, suppose the latent positions were known and define the increments

$$y_t = z_t - z_{t-1}, \qquad t = 2, \ldots, T$$

Let $n = T - 1$ be the number of transitions and assume

$$y_t \sim \mathcal{N}(\lambda^* \Delta t, (\sigma^*)^2 \Delta t)$$

independently. The drift MLE is

$$\hat{\lambda} = \frac{1}{n\Delta t} \sum_{t=2}^{T} y_t$$

so

$$\mathbb{E}[\hat{\lambda}] = \lambda^*$$

Thus, in this idealized Gaussian setting, the drift estimator has no finite-sample bias.

When λ is estimated from the same increments, however, the MLE for diffusion variance is

$$\hat{\sigma}^2 = \frac{1}{n\Delta t} \sum_{t=2}^{T} (y_t - \bar{y})^2, \qquad \bar{y} = \frac{1}{n} \sum_{t=2}^{T} y_t$$

Taking expectation gives

$$\mathbb{E}[\hat{\sigma}^2] = \frac{n-1}{n} (\sigma^*)^2$$

This is the usual finite-sample variance bias associated with using the same data to estimate the mean displacement and the residual variance. It is most visible for short events, where the number of transitions n is small. The bias is for $\hat{\sigma}^2$; because the square-root transformation is concave, $\hat{\sigma}$ itself can be slightly more downward-biased.

Observed-data HMM. In the HMM, positions are not observed directly, and the increments are replaced by posterior-weighted pairwise marginals $\xi_t(i, j)$. At the true parameter value, the expected score is zero under the correctly specified model, so the observed-data MLE is consistent in the usual large-sample regime. However, for a single SWR event the effective number of informative transitions can be small, and the same variance-estimation effect remains: estimating the drift from the event consumes one degree of freedom, causing the posterior-weighted residual variance to be biased downward. This explains why $\hat{\sigma}$ can be systematically underestimated even when $\hat{\lambda}$ shows little systematic bias.

Extension to the switching HMM. In the switching model, the transition kernel depends on the dynamical regime. Only transitions assigned to the drift-diffusion regime contribute directly to the score for (λ, σ). Let $\star$ denote the drift-diffusion regime, and define the drift-diffusion pairwise posterior

$$\xi_t^{(\star)}(\ell, i, j) = p(c_{t-1} = \ell, c_t = \star, z_{t-1} = z^i, z_t = z^j \mid x_{1:T})$$

where the sum over ℓ accounts for the previous regime. The total posterior weight of drift-diffusion transitions at time t is

$$w_t^{(\star)} = \sum_{\ell=1}^{C} \sum_{i,j} \xi_t^{(\star)}(\ell, i, j) = p(c_t = \star \mid x_{1:T})$$

The score equations become

$$\hat{\lambda} = \frac{\sum_{t=2}^{T} \sum_{\ell,i,j} \xi_t^{(\star)}(\ell, i, j) d_{ij}}{\Delta t \sum_{t=2}^{T} w_t^{(\star)}}$$

and

$$\hat{\sigma}^2 = \frac{\sum_{t=2}^{T} \sum_{\ell,i,j} \xi_t^{(\star)}(\ell, i, j) \left(d_{ij} - \hat{\lambda}\Delta t\right)^2}{\Delta t \sum_{t=2}^{T} w_t^{(\star)}}$$

Thus the relevant sample size is not the full SWR duration, but the number of transitions effectively assigned to drift-diffusion. A useful approximation is Kish's effective sample size,

$$N_{\text{eff}}^{(\star)} = \frac{\left(\sum_{t=2}^{T} w_t^{(\star)}\right)^2}{\sum_{t=2}^{T} \left(w_t^{(\star)}\right)^2}$$

Replacing n by $N_{\text{eff}}^{(\star)}$ gives the approximate finite-sample expectation

$$\mathbb{E}[\hat{\sigma}^2] \approx \frac{N_{\text{eff}}^{(\star)} - 1}{N_{\text{eff}}^{(\star)}} (\sigma^*)^2$$

The bias can therefore be larger in the switching HMM than in the single-regime HMM when drift-diffusion occupies only part of the event or when the regime posterior is diffuse.

Practical considerations. The derivation above uses an ideal Gaussian transition on an unbounded track. The implementation evaluates this kernel on a finite spatial grid. Near track boundaries, or for very broad diffusion kernels, the finite-grid transition can deviate from the ideal Gaussian calculation. These implementation details can modify the exact bias, but the main qualitative effect remains: short effective drift-diffusion segments tend to produce underestimated diffusion variance because the same limited transitions are used to estimate both the mean drift and the residual spread.
