# Supplementary material for: Drift-diffusion dynamics of hippocampal replay
Source: PLoS Comput Biol. 2026 Sep 22;22(9):e1014761. doi: 10.1371/journal.pcbi.1014761 (PMC13614660; doi:10.1371/journal.pcbi.1014761)
Supplement: Supplementary file 8 [file pcbi.1014761.s023.pdf]

Response to the reviews

Summary

We thank the reviewers for their constructive feedback as well as their overall positive assessment of our work. We have revised the manuscript according to their suggestions. Below, we first provide a brief summary of the key changes made in the revision. The point-by-point response can be found afterwards.

1. We have separately analyzed the awake and sleep SWRs and report these results throughout the revised manuscript. Importantly, the main conclusions remained consistent with what we initially reported. We found a higher fraction of drift-diffusion events during awake rest compared to post-run sleep. This also leads to clearer interpretations of some of the results, particularly when comparing our findings with prior studies conducted in various brain states.
2. We further strengthened the validation and testing of the modeling framework. In addition to the parameter recovery analysis, we have also incorporated a residual analysis comparing the switching-HMM position estimates with memory-less Bayesian decoding. The residuals were generally small relative to the track length, indicating that the switching-HMM estimates remained close to the position information supported by instantaneous spike activity.
3. We performed a set of control analyses based on detecting SWRs using multi-unit activity (MUA). We found that the majority of candidate SWR events overlapped with MUA-detected population bursts. Repeating the dynamics and replay speed analyses on the matched subset produced results that were consistent with what we initially reported.
4. We carefully revised the text to address the interpretation of the results. In particular, we now clarify the behavioral and recording contexts of comparisons across datasets, and we provide a more careful interpretation of stationary dynamics in relation to the shuffle-control analyses. We also discuss pre-existing hippocampal activity more precisely, and acknowledge the scope of the model assumptions more clearly.

In making these changes, we feel that the manuscript has been substantially improved. So we would like to thank the reviewers again for these valuable suggestions.

Point-by-point response

Reviewer #1: The manuscript presents a switching hidden Markov model that characterizes hippocampal population dynamics during sharp-wave ripples as a mixture of drift-diffusion, stationary, and uniform regimes. Applied to the Pfeiffer & Foster (2015) dataset, they find that ~20% of SWRs exhibit drift-diffusion dynamics at speeds nearly an order of magnitude faster than running, that MSD scaling is quadratic (inconsistent with pure Brownian motion), and that preplay is detectable only under the weakest shuffle control. The modeling framework is well-motivated and the parameter recovery analyses are solid. The switching HMM framework is a methodological advance over traditional two-step replay detection; by jointly inferring dynamics and positions in a single model, it avoids the problems of first committing to a decoded trajectory and then testing it against a template. The drift and diffusion parameters are interpretable and map directly onto biologically meaningful quantities (replay speed and trajectory coherence). However, several issues, most critically, the pooling of sleep and awake SWRs and the lack of controls for non-spatial activity in the decoder, limit the interpretability of the key results and weaken the paper's claims to resolve discrepancies with prior work.

Major Points

1. Awake and sleep SWRs are pooled without justification or separate analysis. The main analyses (Applications 1 and 2) pool awake immobility and post-run sleep SWRs, a fact stated only in the Figure 5 caption and not discussed in the text. This is a significant problem because the prior work the authors compare against was brain-state specific: Stella et al. [31] analyzed SWRs during post-exploration rest/sleep in a separate rest box after 2D open-field foraging (finding Brownian-like dynamics), and Denovellis et al. [25] analyzed awake SWRs on linear tracks (finding real-world speeds). Sleep and awake replay are known to differ in temporal compression, sequential structure, and content (Joo & Frank, 2018, Nat Rev Neurosci). The authors claim their results "clarify" or "resolve" these prior discrepancies, but without separating brain states, they cannot determine whether the discrepancy reflects genuine methodological differences or simply the mixing of two populations with different dynamics. The authors should present the key analyses (speed distributions (Fig. 6), stationary fractions (Fig. 5), and MSD scaling (Fig. 7)) separately for awake and sleep SWRs. This is feasible with the existing dataset. At minimum, the brain state composition of the analyzed events should be clearly stated in the results text.

Response: This is a great point. We agreed that it makes sense to separately analyze the awake and sleep SWRs. Following this suggestion, we have performed an additional analysis by separating awake and post-run sleep SWRs. This is relevant for the composition of the spatiotemporal dynamics of SWRs, replay-speed distributions, tests of the Brownian motion hypothesis, and the structure of pre-run sleep SWRs. One new finding from this analysis is that there is a substantially higher fraction of SWRs that were classified as drift-diffusion events for awake state compared to sleep. We also find that the slope that characterizes the scaling between root-mean-square displacement and time (in Application 2) is smaller for post-run sleep SWRs than awake SWRs. The main results remain consistent with respect to awake vs. sleep SWRs.

We have generated several new figures (also included below) and incorporated these results and their interpretation in the revised version of the manuscript. We have also revised the Results text and figure captions to clearly specify the brain-state composition of the analyzed events. In places where we compare our results with prior studies, we now state the context of each comparison more clearly. We felt that these additional results further strengthen the paper. Thank you for this important suggestion.

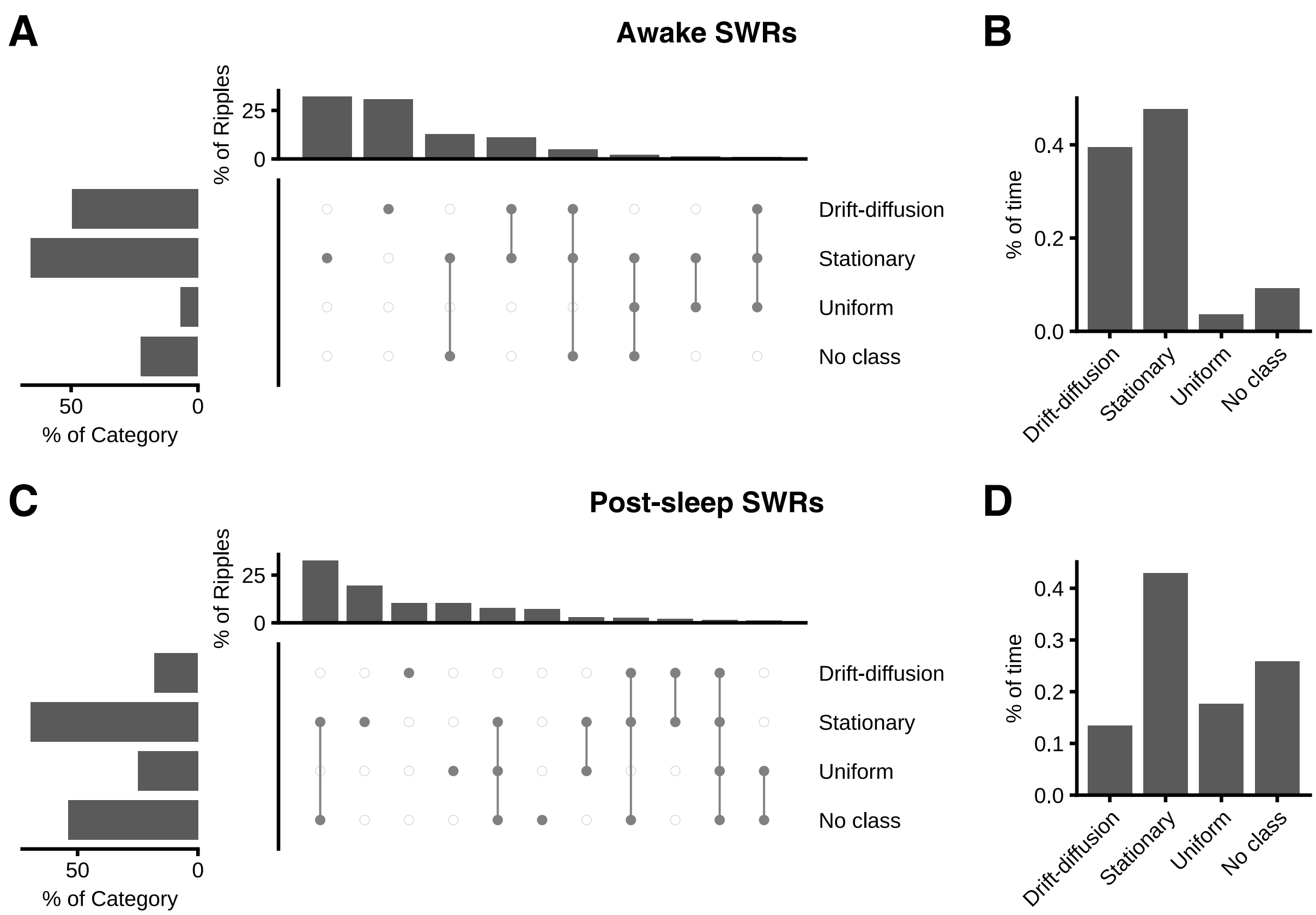

Figure 4: Composition of inferred spatiotemporal dynamics in awake and post-sleep SWRs. The marginal posterior of dynamics of each SWR was assigned a class label following the procedure described in Fig. 2. For event-level summaries, a dynamic class was counted as present in an SWR only when it persisted for at least two consecutive 10 ms bins. **A**. UpSet-plot Lex et al. (2014) summary of dynamic-class combinations in awake SWRs. The top bar plot shows the percentage of SWRs exhibiting each class combination, as indicated by the dot matrix below. Filled dots mark classes present in the combination, connected filled dots indicate co-occurring classes within the same SWR, and open dots mark absent classes. The left horizontal bar plot shows the percentage of SWRs containing each individual class. **B**. Fraction of awake SWR time bins assigned to each dynamic class. **C**. UpSet-plot summary of dynamic-class combinations in post-sleep SWRs, using the same conventions as in **A**. **D**. Fraction of post-sleep SWR time bins assigned to each dynamic class. Only class combinations occurring in more than 1% of SWRs are shown.

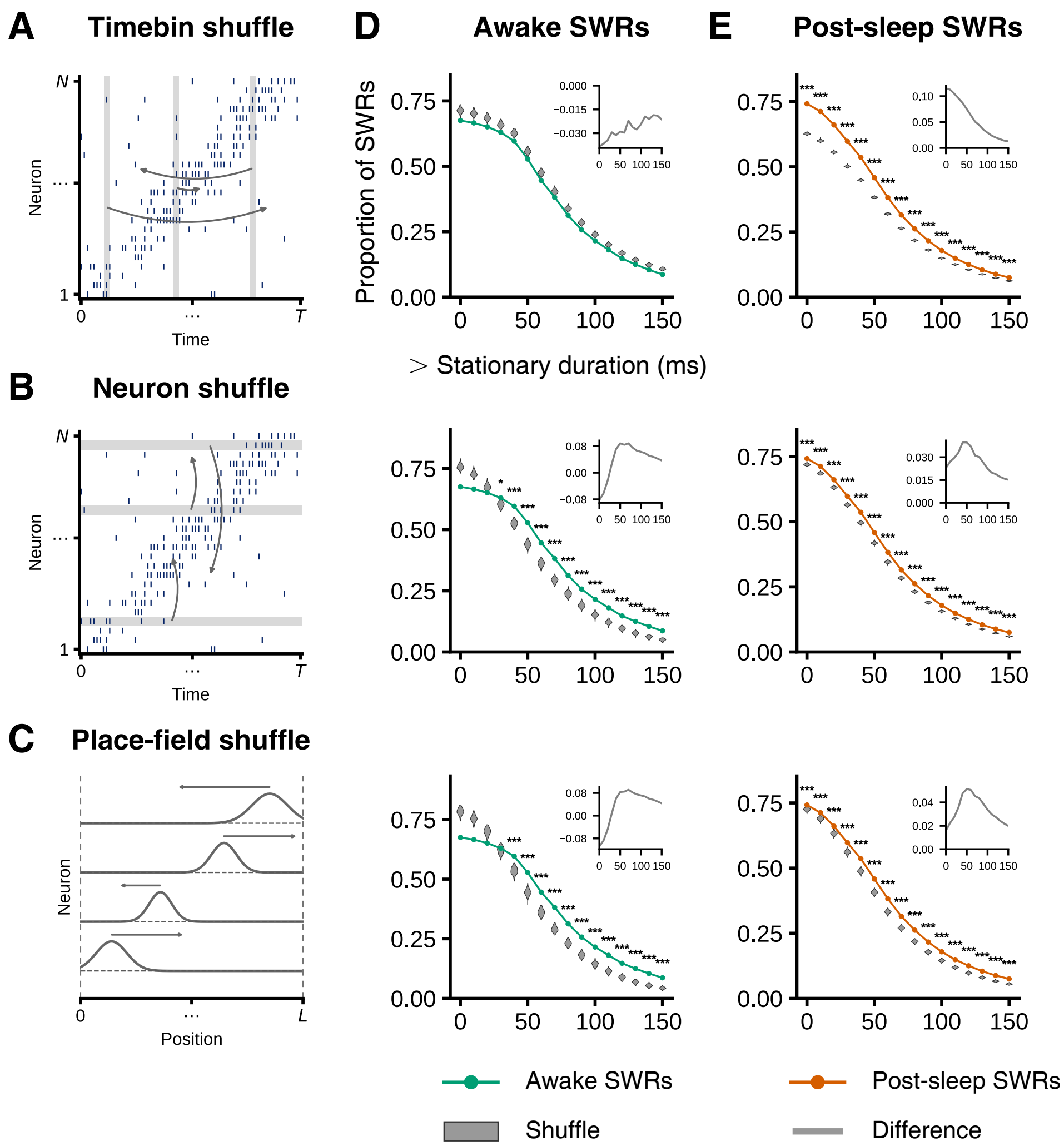

Figure 5: The fraction of stationary events in awake and post-sleep SWRs slightly exceeds chance levels. A–C. Schematic of the three shuffle controls used throughout the shuffle analyses. **A**. Time-bin shuffle, which permutes time bins within each SWR and disrupts temporal ordering while preserving the population activity in each time bin. **B**. Neuron-identity shuffle, which permutes neuron indices and disrupts the correspondence between spiking activity and place-field order. **C**. Place-field rotation shuffle, which circularly shifts each neuron's place field along the linear track while preserving field shape. **D,E**. Real-versus-shuffle comparison of stationary duration in **D** awake and **E** post-sleep SWRs. Rows correspond to the shuffle controls in **A–C**: time-bin shuffle, neuron-identity shuffle, and place-field rotation shuffle. For each SWR, stationary duration was defined as the total time assigned to the stationary regime from the marginal posterior of dynamics. Curves show the proportion of real SWRs whose stationary duration exceeded each threshold on the x-axis. Gray violins show the corresponding distribution from 120 shuffle repeats per SWR. Insets show the difference between the real proportion and the mean shuffled proportion across thresholds, where positive values indicate more stationary structure in the data than expected under the shuffle. Asterisks indicate one-sided permutation tests comparing real data against the shuffle distribution (*$p < 0.05$, **$p < 0.01$, ***$p < 0.001$).

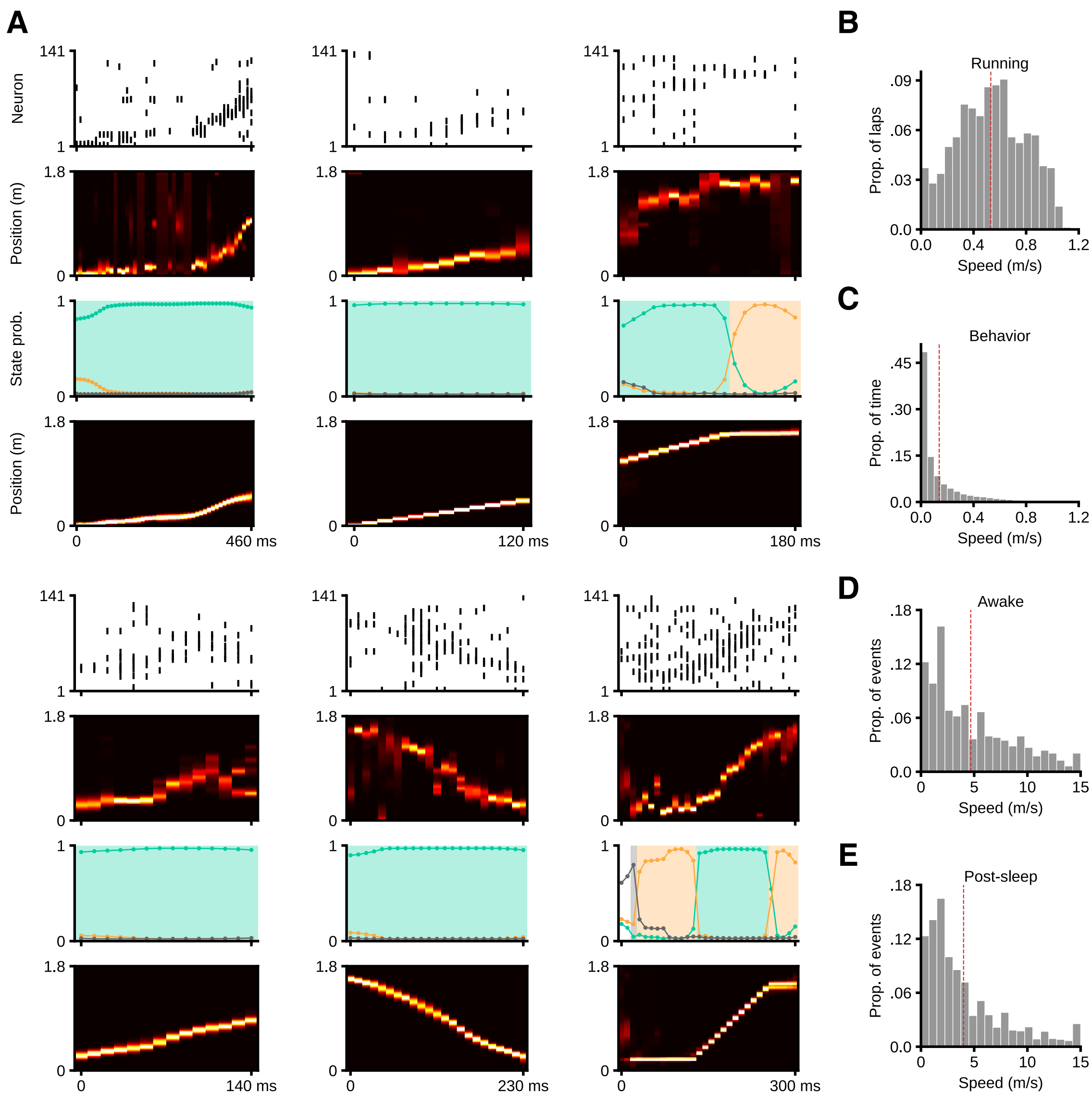

Figure 6: Replay trajectories are substantially faster than overt behavior. A. Representative events with different replay speeds. Plotting conventions follow Fig. 2B–H: spike raster, memory-less Bayesian position posterior, switching-HMM dynamics posterior, and switching-HMM position posterior are shown from top to bottom for each event. **B**. Distribution of per-lap running speeds on the linear track. Running speed was estimated for each traversal by fitting a linear regression to position as a function of time. **C**. Distribution of instantaneous behavioral speeds during running. Behavioral speed was estimated from the finite-difference derivative of tracked position. **D**. Distribution of replay speeds for awake events classified as drift-diffusion events. **E**. Distribution of replay speeds for post-sleep events classified as drift-diffusion events. Replay speed was estimated from the inferred drift rate. Drift-diffusion events were defined as events with at least five consecutive 10 ms bins assigned to the drift-diffusion regime. Red dashed lines indicate distribution means: running, 0.531 m/s; behavior, 0.138 m/s; awake replay, 4.677 m/s; post-sleep replay, 4.002 m/s.

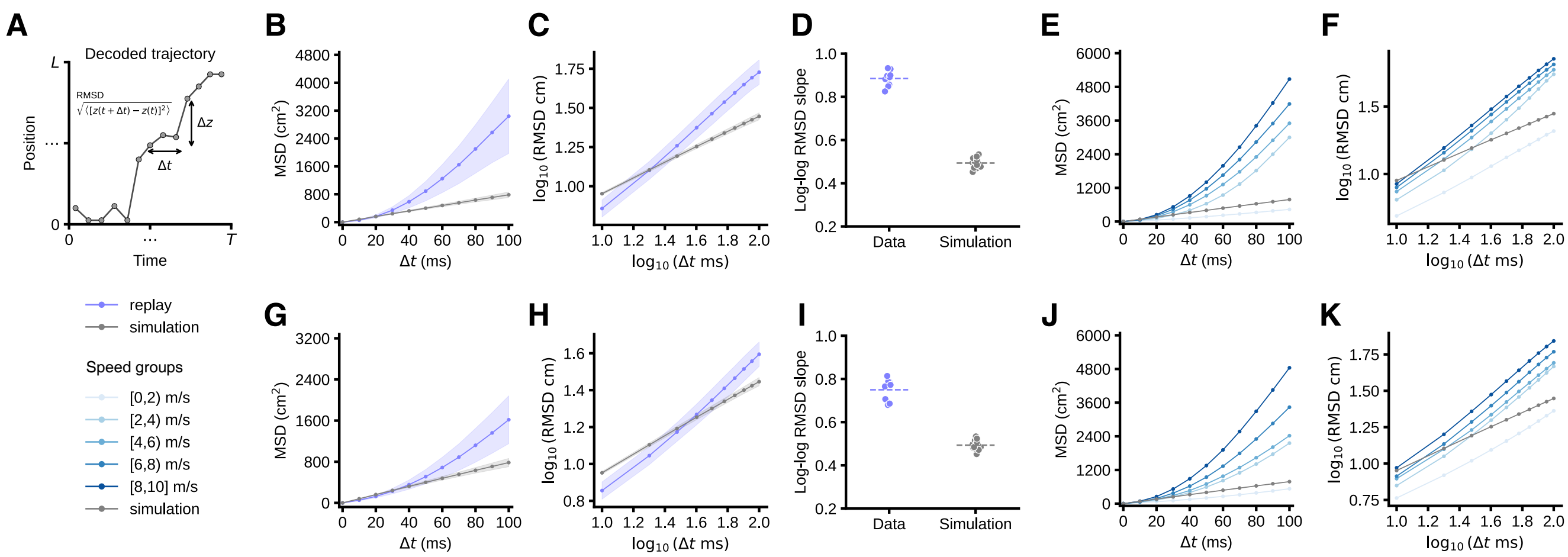

Figure 7: Replay trajectory differs from pure diffusion dynamics (Brownian motion), and is more consistent with drift-diffusion. A. Schematic of the displacement analysis. For a decoded latent trajectory, displacement was measured as $\Delta z = |z(t + \Delta t) - z(t)|$ over time lags Δt. Mean-square displacement (MSD), $\langle \Delta z^2 \rangle$, and root-mean-square displacement (RMSD), $\sqrt{\langle \Delta z^2 \rangle}$, were then computed across trajectory samples. **B–F**. Displacement analysis applied to awake SWRs classified as drift-diffusion events. Replay trajectories were decoded under a fixed pure-diffusion model ($\sigma = 0.8$ m/$\sqrt{\text{s}}$) and compared with pure Brownian-motion simulations Krause and Drugowitsch (2022). **B**. MSD as a function of Δt for replay (purple) and simulated diffusion trajectories (gray). **C**. Log-log RMSD as a function of Δt for the same replay and simulation data. **D**. Slopes from linear fits to the log-log RMSD curves, with each point representing one recording session or one simulated session. Dashed horizontal lines indicate session means. **E**. MSD as a function of Δt for awake replay trajectories grouped by inferred replay speed. **F**. Log-log RMSD scaling for replay trajectories grouped by inferred replay speed. **G–K**. Displacement analysis applied to post-sleep SWRs classified as drift-diffusion events. **G**. MSD for replay and simulated diffusion trajectories. **H**. Log-log RMSD for replay and simulated diffusion trajectories. **I**. Slopes from linear fits to log-log RMSD curves. **J**. MSD by replay-speed group. **K**. Log-log RMSD scaling by replay-speed group. Speed groups are $\lambda \in [0, 2), [2, 4), [4, 6), [6, 8)$, and $[8, 10]$ m/s. In **C**, **D**, **H**, and **I**, solid lines and shaded bands show the mean ± 1 standard deviation across sessions. Mean log-log RMSD slopes were 0.885 for awake replay, 0.750 for post-sleep replay, and 0.493 for simulated Brownian trajectories.

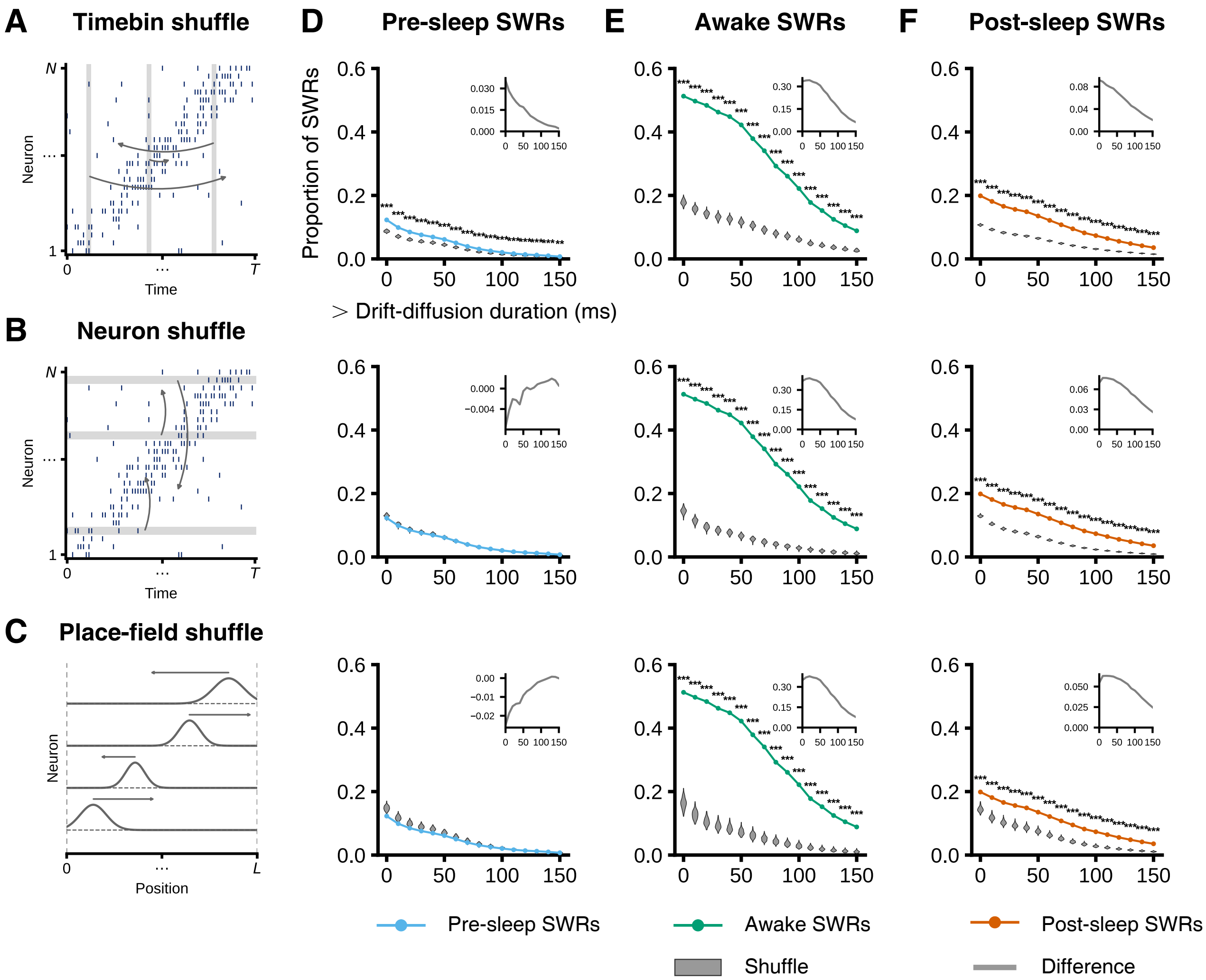

Figure 8: Preplay events show substantially weaker temporal structure than replay. A–C. Schematic of the same shuffle controls described in Fig. 5: time-bin shuffle, neuron-identity shuffle, and place-field rotation shuffle. **D–F**. Real-versus-shuffle comparison of drift-diffusion duration in **D** pre-sleep, **E** awake, and **F** post-sleep SWRs. Rows correspond to the shuffle controls in **A–C**: time-bin shuffle, neuron-identity shuffle, and place-field rotation shuffle. For each SWR, drift-diffusion duration was defined as the total time assigned to the drift-diffusion regime from the marginal posterior of dynamics. Curves show the proportion of real SWRs whose drift-diffusion duration exceeded each threshold on the x-axis. Gray violins show the corresponding distribution from 120 shuffle repeats per SWR. Insets show the difference between the real proportion and the mean shuffled proportion across thresholds, where positive values indicate more drift-diffusion structure in the data than expected under the shuffle. Asterisks indicate one-sided permutation tests comparing real data against the shuffle distribution (*$p < 0.05$, **$p < 0.01$, ***$p < 0.001$).

2. The decoder assumes all SWR activity encodes a position on the track. The model assumes that at every time bin during an SWR, the hippocampal population is encoding some position on the current track. However, a substantial fraction of SWR activity may not encode any spatial position. It may represent a different environment, non-spatial information, or simply lack coherent spatial content. When such activity is forced through the Bayesian decoder, it will produce posteriors biased toward positions with high place field density, likely classified as "stationary" or "uniform." This means these categories may be substantially inflated by non-spatial activity rather than reflecting genuine spatial dynamics. Figure S4 in the SI provides direct evidence for this concern: decoded positions during stationary periods cluster heavily at the track ends (0 and 1.8 m), exactly where reward wells are located and place field density is likely non-uniform. I would suggest the authors test this with a control analysis. For example, decoding SWR activity using place fields from a different recording session or environment, or through simulation. This would characterize the model's false positive profile when activity genuinely does not encode positions on the current track. Understanding this baseline is essential for interpreting the ~80% of SWRs that lack drift-diffusion dynamics. If possible, it would be a significant improvement to the method to include a "not-on-track" state, though I recognize that this is not a straightforward addition to the modeling framework.

Response: Thank you for these thoughtful comments.

The reviewer noted that, if SWR activity does not encode position on the current track, a decoder that requires every time bin to occupy a track position could assign posterior probability to positions favored by the place-field structure and thereby potentially inflate the “stationary” or “uniform” categories. We agree that this is an important point to take into account when interpreting the non-drift-diffusion components in our results, especially for the stationary dynamics. We believe that this issue can be at least partially addressed by comparing the fraction of stationary events from the data with shuffles. We have added additional analyses using three different shuffle controls: time-bin shuffle, neuron shuffle, and place-field shuffle. We have carefully revised the Results section to compare our decoding results against these shuffles. In particular, when reporting results around stationary dynamics, we now emphasize the comparison with these controls beyond interpreting the raw stationary fraction alone. As another effort to address this concern, we also added an analysis comparing the decoded positions of stationary periods in the real data against the decoded positions of stationary periods in each of the three shuffle controls (see Fig. S5 and Fig. S6).

The reviewer also suggested that the model could be improved by adding a “not-on-track” state to capture activity that does not encode position in the current environment. We agree that adding such a state would be valuable, because it would explicitly separate weak current-environment spatial evidence from position representations on the analyzed track. As the reviewer correctly pointed out, adding this additional state is indeed not as straightforward. In particular, it requires the likelihood estimation to involve both real data and an appropriate null distribution for activity that is not represented on the current track. This would change the overall structure of the probabilistic inference. We would also like to mention that another way to address this concern would be to model the latent state as a probabilistic distribution over location (instead of a point or delta function in the generative model). In this formulation, if the neural activity represents states on a different track, it would correspond to a flat probability distribution for locations on the current track. This formulation also naturally takes into account the uncertainty of the state when the neural activity represents the current track. While we consider these important future extensions, we also feel that they are beyond the scope of the current paper. We have added a discussion of these points in the Discussion section. Now the text reads:

"*Third, the present model assumes that the latent state of neural activity at any moment represents a position in the current environment. Neural activity representing another environment or only weakly representing spatial information of the current environment may also be present in the data. To further improve the model, one can incorporate a "not-on-track state" or, more generally, model the latent state as a distribution over position.*"

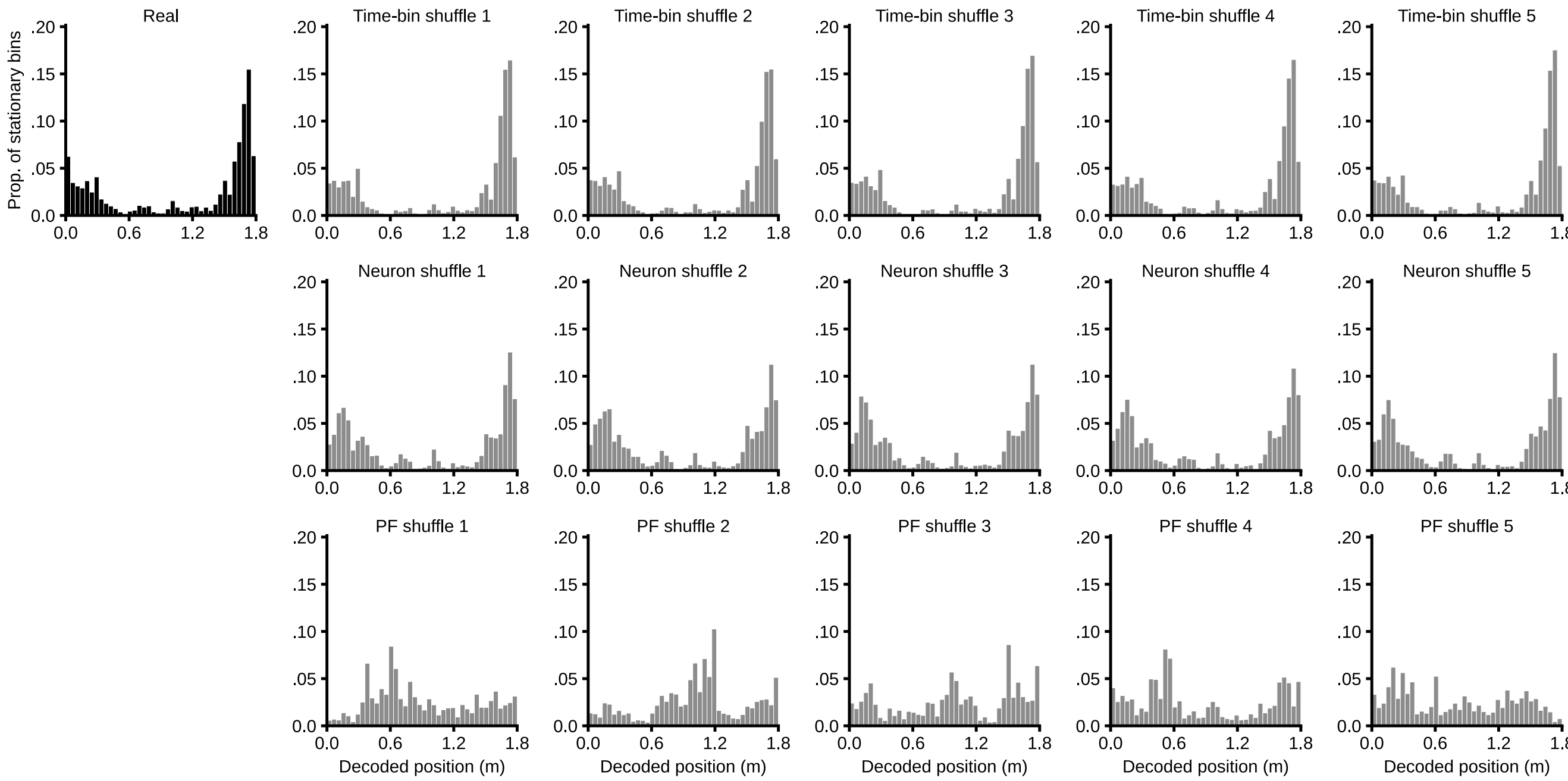

Figure S5: Decoded position distributions for stationary dynamics in real data and shuffles from one session of rat Janni. Histograms show the decoded positions of time bins classified as stationary, pooled across awake and post-sleep SWRs. For each stationary bin, decoded position was defined as the maximum of the position posterior after marginalizing over dynamics states. Bar heights give the proportion of stationary bins in each position interval along the 1.8 m track. The dark gray histogram at upper left shows the distribution for the real data. Gray histograms show five independent realizations of each control: time bins were permuted within each SWR (top row), neuron identities were permuted within each SWR (middle row), or each neuron's place field was independently circularly shifted along the track (bottom row). The model was fit and posterior inference was performed separately for each shuffled realization.

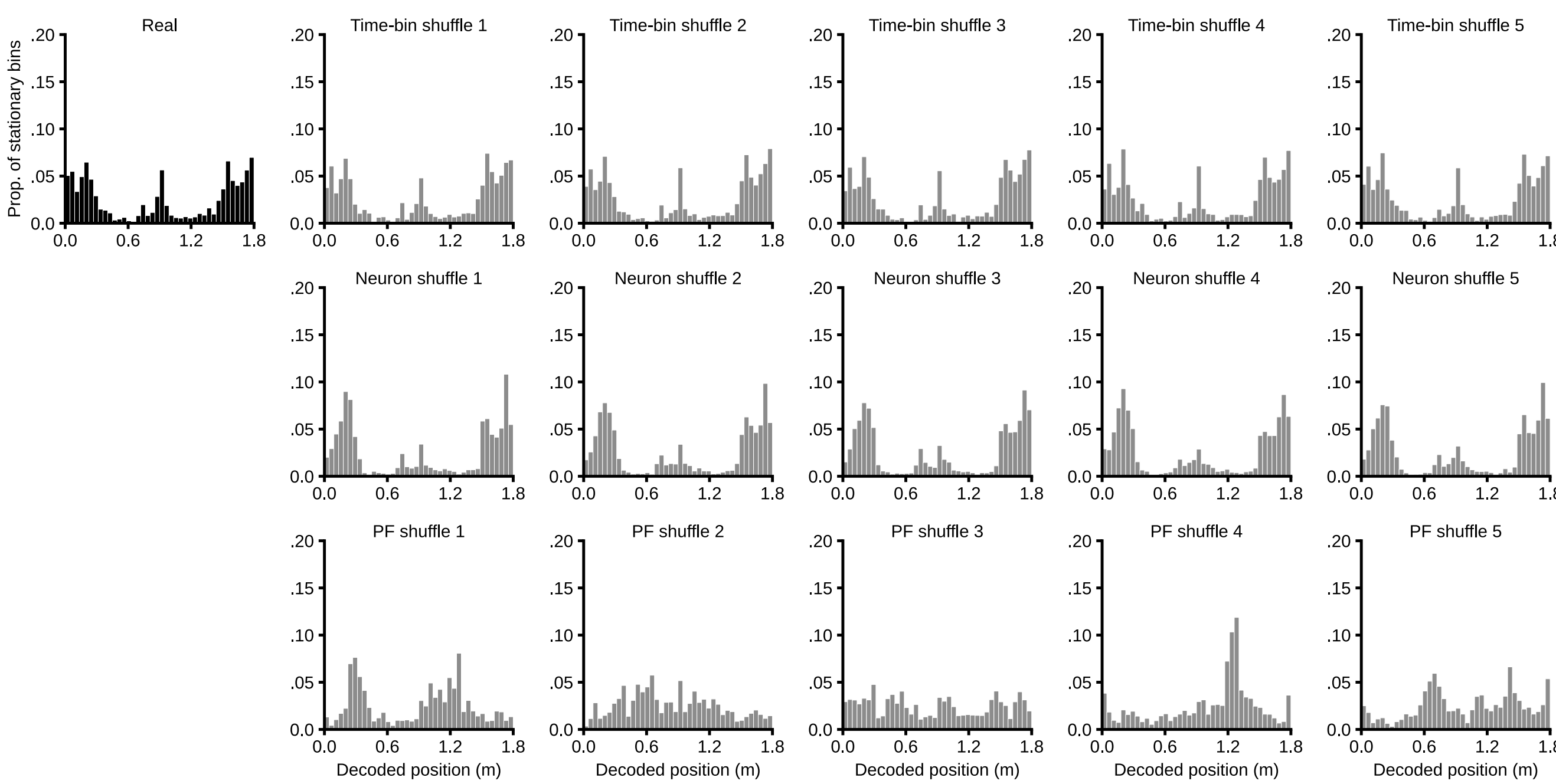

Figure S6: Decoded position distributions for stationary dynamics in real data and shuffles from one session of rat Ettin. Same analysis and conventions as in Fig. S5, shown for a different recording session.

3. The high false positive rate for stationary classification needs more rigorous treatment. The shuffle analyses (Fig. 5) show that shuffled (temporally meaningless) data produces nearly as many "stationary" classifications as real data (the difference is only ~5%). The authors note this finding but do not adequately address its implications. Several issues: (a) The "no class" category is never discussed in the main text. Figure S6B shows that for pre-run SWRs, "no class" accounts for ~27% of total SWR time, comparable to stationary and uniform. This is a substantial fraction that deserves discussion. The authors should report the "no class" prevalence for both pre- and post-run conditions and explain what this category represents. (b) The discussion (p. 21) hypothesizes two reasons for false stationary detection (heterogeneous place field sampling and high-firing-rate neuron subsets) but tests neither, despite Figure S4 providing relevant evidence for the former. These hypotheses should be tested with the current data. One possible solution is replacing the uniform distribution with a fitted stationary distribution that formalizes the null hypothesis of non-uniform but random sampling in the data. (c) The conceptual distinction between "uniform" and "no class" is unclear. If activity has no spatial structure, would the model assign it to "uniform" or "no class"? This needs clarification.

Response:

The reviewer noted that the “No Class” category should be defined and reported explicitly. This is a good point and we apologize for the confusion. To address this issue, we have revised the manuscript to give a clearer definition of “No Class” in the Fig. 2 caption. We have also revised the figures and Results text to report the prevalence of “No Class” for pre-run sleep, awake-rest, and post-run sleep SWRs.

The reviewer also raised the point that the stationary category might include false positives generated by non-spatial activity or by decoder biases related to non-uniform place-field sampling. We agree that the high stationary rate under shuffle controls is important and should be treated more carefully. We have revised the text in the Results section. Now the results on stationary dynamics are compared against three shuffle-control analyses: time-bin shuffle, neuron shuffle, and place-field shuffle. We also emphasize these control comparisons when interpreting stationary dynamics. In addition, we compared the decoded positions of stationary periods in the real data against the decoded positions of stationary periods in each of the three shuffle controls.

The reviewer further noted that the conceptual distinction between “Uniform” and “No Class” was unclear. We would like to provide the following clarification. Our model infers the dynamics of a time bin based on how its spiking pattern, and therefore the underlying position encoding, relates to temporally nearby time bins. For “Uniform” dynamics, the time bins still carry position information within the model, but the inferred sequence of latent positions forms a non-coherent structure. In contrast, “No Class” indicates that one cannot clearly identify strong alignment of the spiking pattern with any of the three candidate dynamics: drift-diffusion, stationary, or uniform.

4. Claims about resolving discrepancies with prior work are confounded by multiple uncontrolled variables. The paper frames its contribution partly as resolving discrepancies between Stella et al. [31] (Brownian motion, post- exploration rest after 2D foraging) and Denovellis et al. [25] (real-world speeds in awake). However, this study differs from both on multiple axes simultaneously: environment dimensionality (1D vs. 2D), behavioral task (back-and-forth running vs. open-field foraging), brain state (pooled awake+sleep vs. state-specific), recording context (on-track immobility vs. separate rest box), and analysis method. Without controlling for these variables, the source of any discrepancy is unidentifiable, yet the abstract claims the approach provides "unambiguous interpretations" and the discussion claims to "resolve" or "clarify" prior inconsistencies. The authors should:

(a) explicitly enumerate the variables that differ between their study and the prior work they compare against,

(b) temper claims about resolving discrepancies accordingly, particularly in the abstract and introduction, and

(c) separate awake and sleep analyses (see Major Point 1), which would at least control for one of these confounds.

Applying the method to a 2D dataset (e.g., the Csicsvari lab data used by Stella et al., if publicly available) would substantially strengthen the paper, but at minimum the claims should be qualified to reflect the specific experimental context: a single 1D linear track dataset with pooled brain states.

Response: The reviewer notes that our comparisons with prior studies are confounded by multiple differences, including environment dimensionality, behavioral task, brain state, recording context, and analysis method. We agree that it is important to state these factors more clearly. We have revised the main text to address this point by making these differences explicit as well as toning down our claims.

As mentioned earlier, we have also revised the relevant figures, figure captions, and the Results section to separately report the results for awake-rest and sleep periods.

The reviewer also suggests that applying the method to a 2D dataset would substantially strengthen the manuscript. We agree that incorporating 2D datasets would be valuable. While extending the model to 2D is feasible, it requires additional model assumptions. In particular, the 2D setting requires decisions about how to parameterize drift-diffusion in a two-dimensional environment, how to represent movement direction and diffusion jointly, and how to sensibly detect jumping events within the same probabilistic framework. We are currently working on this extension and plan to report it in a future study. We have revised the Discussion section to further elaborate on the possibility of extending our model framework to 2D environments. Now the text reads:

"*Other types of environments have also been used in the study of replay, such as 2-D open fields and 1-D environments with more complex geometry. Adaptation of our approach to these environments should be feasible, but would require modifications to the probabilistic transition matrix for latent positions. In particular, extending the model to a 2-D environment would require additional considerations on how to parameterize drift and diffusion in a two-dimensional environment jointly and how to detect jumping events within the same probabilistic framework.*"

Minor Points

1. The paper conflates SWRs and replay throughout (e.g., "dynamics of replayed positions z during putative replays (population activity during SWRs)."). These are distinct phenomena: many SWRs do not contain replay (as the paper itself shows, only ~20% exhibit drift-diffusion dynamics), and there are many replay events that occur not during SWRs (Widloski & Foster, 2025, Nat Comms). The authors should use more careful terminology, distinguishing between SWR events (the electrophysiological phenomenon) and replay events (the subset with sequential spatial content).

Response: The reviewer notes that SWRs and replay events are distinct phenomena and the terminology should be used precisely. We completely agree. We have carefully revised the term usage throughout the manuscript, especially in the Methods section and figure legends. Where the analysis refers to the electrophysiological events detected in the data, we use terms such as “SWRs” or “SWR events.” Where the analysis refers specifically to sequential spatial content inferred within SWRs, we use “replay” or clearer phrases such as “drift-diffusion events within post-sleep SWRs.”

2. The drift-diffusion framework is a specific parametric assumption. Replay trajectories could exhibit dynamics that this model cannot capture. For example, Levy flight-like jumps between distant locations, acceleration/deceleration within events, or non-stationary drift rates. Recent modeling work has shown that continuous attractor networks with firing rate adaptation can generate replay dynamics spanning stationary, Brownian-diffusive, and super-diffusive regimes (Dong, Chu, Huang, Ji & Wu, NeurIPS 2021; Ji et al., Nat Comms 2026).

The paper validates parameter recovery from simulated data (Fig. 3), but does not assess goodness of fit to real data. A goodness-of-fit analysis (e.g., comparing likelihood to a more flexible nonparametric model, or examining residuals) would strengthen confidence that drift-diffusion captures the relevant structure in replay dynamics.

Response: The reviewer points out that the parameter recovery experiments using simulations do not by themselves establish goodness of fit to real data and that a direct goodness-of-fit analysis would strengthen the interpretation of the fitted dynamics. We have revised the manuscript to add Fig. S2, which shows a residual analysis comparing our position decoding with memory-less Bayesian decoding. For the three dynamics considered in the model, the residual error is much lower than the shuffle control and remains small. In awake SWRs, the 75th percentiles of the absolute residual were 12 cm for drift-diffusion bins, 10 cm for stationary bins, and 10 cm for uniform bins on a 180 cm track. In post-sleep SWRs, the corresponding values were 20, 32, and 2 cm. These values were far below the time-permuted controls, whose 75th percentiles were 116, 138, and 100 cm in awake SWRs and 112, 128, and 110 cm in post-sleep SWRs. This analysis indicates that the switching-HMM decoding remains close to the position information supported directly by the instantaneous spike activity. We would also like to note that our model includes switching dynamics to account for temporal structure in the position posterior. Therefore, we expect some difference between our decoding and memory-less Bayesian decoding, and the residual analysis is intended to verify that this difference is modest, rather than a large mismatch between the model and the spike-based position evidence.

The reviewer also notes that the drift-diffusion model is a specific parametric assumption and may not capture all possible replay dynamics, such as Levy flight-like jumps, within-event acceleration or deceleration, or non-stationary drift rates. We have revised the Discussion section to discuss the choice of parametric assumptions more clearly. It is useful to constrain the parametric space to be simple, as the parameters are estimated from a small amount of data for each individual SWR. In this setting, a more flexible parameterization could fit more trajectory forms, but would also increase the risk of unstable or non-identifiable event-level estimates. Nevertheless, we believe that the current parametric setting is flexible and trustworthy enough to capture the major spatiotemporal dynamics in SWRs that are the focus of this manuscript. This is supported by the new goodness-of-fit residual analysis, which shows that the decoded positions remain close to the memory-less Bayesian decoder, and by the recovery analysis, which shows that the maximum-likelihood estimates can recover the model parameters from simulated data.

The reviewer further mentioned recent modeling studies showing that continuous attractor networks with firing-rate adaptation can generate stationary, Brownian-diffusive, and super-diffusive replay-like dynamics. Thank you for bringing these to our attention. These studies are definitely relevant. These studies develop mechanistic models for the neural dynamics of SWRs, while our study provides statistical estimation procedures to infer the neural dynamics from the data. Thus the two approaches are complementary to each other. We have revised the Discussion section to discuss the mentioned recent studies (Dong et al., 2021; Ji et al., 2026) and their relevance in interpreting the mechanistic origins of the dynamics we infer. Citing these studies enables us to place our work in a broader context and slightly strengthen the paper. Thank you for this suggestion. We have added the following text:

"*Mechanistically, recent computational models show that firing-rate adaptation in continuous attractor neural networks (CANNs) can generate stationary, diffusive, and super-diffusive dynamics that resemble replays [77,78]. These studies address how such dynamics may arise from network computations, whereas the present framework estimates these dynamics statistically from population recording data, and thus is synergistic with the recent mechanistic modeling.*"

3. The preplay analysis compares pre-run SWRs only to post-run sleep replay. Since awake replay is generally more structured, this comparison may make preplay look relatively stronger than it would against the most stringent comparator. The authors should note this. Additionally, the prior preplay literature (e.g.,

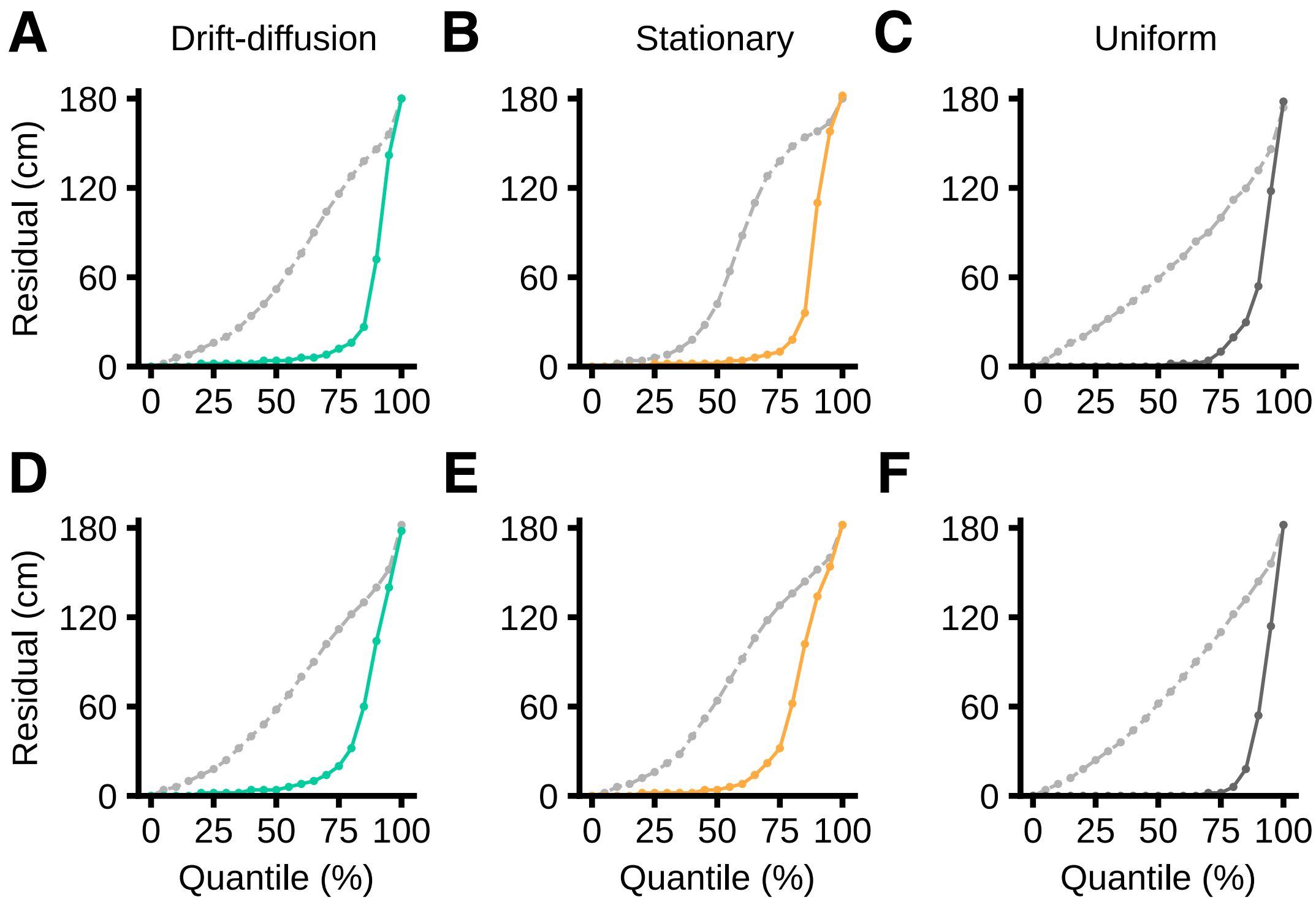

Figure S2: Residuals between switching-HMM and Bayesian decoded positions during sharp-wave ripples. Quantiles of the absolute position residual between the maximum a posteriori (MAP) estimate from the switching-HMM position marginal and the MAP estimate from the memory-less Bayesian decoder. Position residuals of SWR time bins are grouped by brain state and assigned dynamics class. Rows, top to bottom, show awake and post-sleep SWRs. Columns, left to right, show drift-diffusion, stationary, and uniform dynamics. Solid colored curves show the residual distributions in SWRs. Dashed light gray curves show a control baseline in which Bayesian MAP positions were permuted across time bins before recomputing the residual.

Dragoi & Tonegawa 2011; Farooq et al. 2019) could be discussed more thoroughly to contextualize where these results land, and it would be good to confirm that the previous methods used do in fact recover preplay in the dataset (per those methods), for comparison.

Response: The reviewer notes that comparing pre-run sleep SWRs only with post-run sleep SWRs could make preplay appear stronger than it would under comparison with awake-rest SWRs, which are often more structured. The reviewer also suggests placing our results more clearly in the context of prior preplay literature. We agree with these suggestions. As mentioned earlier, we have now separated pre-sleep, awake-rest, and post-sleep SWRs throughout the relevant analyses, allowing a clearer comparison among these brain states. We have revised the text in the Discussion and SI to state the relationship to the preplay literature more precisely, particularly the studies by Dragoi and Tonegawa (2011) and Farooq et al. (2019). We would like to note that, in our analysis, evidence for preplay is observed only under the time-bin shuffle control. This is generally consistent with Farooq et al. (2019), which relied mainly on a time-bin shuffle for detecting pre-existing spike patterns in SWRs.

4. The gain parameter γ in the emission model (Section 4.4.2, which scales place field firing rates for SWRs) is never specified. It is not listed among the optimized parameters $\{\lambda, \sigma, q\}$, and no fixed value is reported. The authors should state how γ is determined, report its value, and discuss sensitivity of results to this choice, since it directly affects decoder confidence and could bias regime classification.

Response: Thank you for pointing this out. We would like to clarify that, in all results presented in this study, we followed the emission-likelihood assumption from Pfeiffer and Foster (2015), with no extra gain rescaling of place-field firing rates during SWRs. Equivalently, $\gamma = 1.0$ for all analyses. This choice is also consistent with Denovellis et al. (2021). We have revised the Methods section accordingly to remove this notation. This

γ parameter was initially considered in an earlier formulation of our model, and we should have removed it in the editing process. We apologize for the confusion.

5. When the authors first apply their model to real data (p. 9), almost no information is provided about the dataset. Key details (recording location, which behavioral epochs are included, number of neurons per session, SWR detection criteria) should be briefly stated at the point of first use, not only in the Methods.

Response: The reviewer notes that the first application of the model to real data did not provide enough immediate context about the dataset, including recording location, behavioral epochs, neuron counts, and SWR detection criteria. Thanks for this suggestion. We have revised this by revising the text. Now, in the Results section, we briefly introduce the key dataset details when the real-data analysis first appears.

6. The "no class" label appears in figures (Fig. 4, Fig. S6) but is never defined in the main text. It should be briefly explained when first referenced.

Response: The reviewer notes that the “No Class” label appeared in figures without a clear definition in the main text. We have revised the manuscript to give a clearer definition of “No Class” in the Fig. 2 caption and in the main text.

Typos and grammar:

- p. 12: "the existence stationary dynamics" $\rightarrow$ "the existence of stationary dynamics"

- p. 17: "cannot be interpreted as conclusively evidence" $\rightarrow$ "conclusive evidence"

- p. 17: "may contribute it" $\rightarrow$ "may contribute to it"

- p. 17: "may cause segments posterior to be consistent over space" – this sentence is garbled and the meaning is unclear

- p. 12: "Though this a conceptually provocative result" $\rightarrow$ "Though this is a..."

- p. 16: "neuron-identify shuffle" $\rightarrow$ "neuron-identity shuffle"

Response: We have fixed these. Thank you for pointing these out and your careful reading!

Reviewer #2

The paper demonstrates a novel method for analyzing replay events in the hippocampus, which improves on prior methods by being more principled rather than ad hoc. The authors use the method to address some outstanding questions in the field of reactivation/replay. In this manner the paper helps advance the field. I do have a few critiques, which I think the authors can address.

Major.

1. The principled system is set up as having three states, two of which (stationary and uniform) are highly constrained, the other of which (drift diffusion–DD) has more free parameters. Especially since a stationary state is a subset of drift diffusion models (just with drift and diffusion parameters of zero) it is important that the extra constraints (which reduce the likelihood of finding a transition to a state) are accounted for.

Moreover, why not allow the DD state to transition to another DD state with any set of different parameters as opposed to only transition to a DD state with drift of zero (the stationary state)?

Finally, on this topic, it would seem that to rule out the stationary state, one could simply provide the confidence interval of DD parameters and show that zero drift is not within the interval. The latter question also applies to the tests on whether data are fitted by a random walk, which is simply a DDM with drift of zero – why not simply calculate the likelihood of parameters for the DDM and show that zero drift has much lower probability than the maximum likelihood drift?

Response:

Thank you for these thoughtful comments. The reviewer notes that the stationary state is a constrained special case of drift-diffusion dynamics, and that this relationship should be handled carefully when interpreting transitions among dynamic states. We agree that the stationary state is a subset of drift-diffusion dynamics, corresponding operationally to a zero-drift, zero-diffusion limit. We would like to point out that the model-recovery analysis in Fig. 3 was designed to validate whether the model behaves as expected when there is a switch among dynamics. When there are significant non-zero-drift DD dynamics, the model recovers the dynamics well. When an SWR contains only stationary dynamics, the drift-diffusion component can collapse operationally toward stationary dynamics with zero drift. Our dynamics classification criterion, outlined in Fig. 2, accounts for this behavior: time bins with strong stationary posterior are classified as stationary, and mixtures of stationary and drift-diffusion evidence are treated as stationary unless there is sufficiently strong drift-diffusion evidence.

The reviewer also mentioned the possibility of allowing transitions from one drift-diffusion state to another drift-diffusion state with a different set of parameters. Indeed, this would be a natural way to represent within-event changes in drift rate or diffusion scale, such as acceleration, deceleration, or changes in movement direction. We constrained the parametric space to be simple because we estimate one set of parameters for each individual SWR, and each SWR contains only a limited amount of data. In this setting, a more flexible parameterization, while being more expressive, could increase the risk of unstable or non-identifiable event-level estimates. Nevertheless, we believe the current parametric setting is flexible and reliable enough to capture the basic patterns of the spatiotemporal dynamics in SWRs, which are the focus of this manuscript. This is additionally supported by the new goodness-of-fit residual analysis (Fig. S2), which shows that the decoded positions remain close to the memory-less Bayesian decoder, and by the recovery analysis, which shows that the maximum-likelihood estimates can recover the model parameters from simulated data. We have revised the text in the Discussion section to make these points clearer. Now the text reads: "*Second, another natural extension would be a switching drift-diffusion model that switches between multiple drift-diffusion processes with distinct parameters or other types of dynamics (such as Levy flight). However, practically the limited amount of data available per SWR event may prevent one from estimating the parameters reliably*."

The reviewer further suggests using statistical tests such as confidence intervals to directly evaluate whether zero drift can explain events classified as drift-diffusion. We have revised the analysis presented in Fig. S3 to compare the replay-speed distribution of drift-diffusion events against a zero-drift pure-diffusion null. For awake-rest and post-sleep drift-diffusion events, replay speed was estimated from the fitted drift rate. For the null distribution, we used the corresponding pure-diffusion model: for each drift-diffusion event, a zero-drift diffusion model was fit by maximum likelihood to estimate the diffusion parameter, and then 10 pure-diffusion trajectories with the same event duration were simulated. The speed of each simulated trajectory was defined as the absolute slope from a linear regression of position against time. This analysis shows that the observed replay speeds are substantially larger than what the zero-drift pure-diffusion null predicts. The mean replay speed was 4.677 m/s for awake-rest events and 4.002 m/s for post-sleep events, compared with 1.488 m/s for the simulated pure-diffusion trajectories. One-sided Welch two-sample tests showed that both awake-rest and post-sleep drift-diffusion events were significantly faster than the pure-diffusion null (awake vs. pure diffusion, $p < 0.001$; post-sleep vs. pure diffusion, $p < 0.001$). Thus, we would like to note that the revised Fig. S3 provides a direct zero-drift control and supports the conclusion that the observed drift-diffusion events are not simply random walks with zero drift.

2. Testing: the authors test spiking implementations of their HMM and assess how traditional models fail to capture some of its features. However, they do not do the converse: for example, traditional methods look at "maximum jump" in decoded location. It is one thing to find that a simulated DDM can appear to be decoded incorrectly as having a jump and to therefore say the new method is better, but that is somewhat tautological.

One also needs to compare simulations where there are jumps in location and see how the HMM proposed decodes the results – does it capture the "ground truth" when the "ground truth" follows the types of measures in previous decoding.

Again, if the HMM allowed for a state transition to the *same type of state but with different parameters* I think it would be more general and capture an event such as stationary at one location followed by a jump to stationary at another location.

With or without this decoding possibility, it is important to test how the new method deals with such situations (which in my view are quite likely/common in the data) as compared with the old methods that directly reveal such "jumps".

Response: The reviewer raises an important point about a complementary direction of our validation: when conventional spike-based decoding supports position trajectories or apparent jumps, does the switching-HMM preserve that information rather than imposing a different trajectory structure. We agree that this is another important control for interpreting the relationship between our model and previous decoding approaches.

We have revised the manuscript to add Fig. S2, which shows a residual analysis comparing our position decoding with memory-less Bayesian decoding. This analysis directly asks whether the switching-HMM decoded position remains close to the position information supported by the instantaneous spike activity, which is the information used by traditional measures such as maximum jump. For the three dynamics considered in the model, the residual error is much lower than the shuffle control and remains small, less than 15 cm on a 180 cm track at the 75% quantile. This result indicates that the switching-HMM decoding recapitulates the position information estimated by memory-less Bayesian decoding, while also providing a temporal-dynamical interpretation of that information. Thus, when previous decoding methods identify strong spike-based position evidence, our model generally remains close to that evidence rather than producing a substantially different decoded trajectory.

We would also like to note that our model includes switching dynamics to account for temporal structure in the position posterior. Therefore, one expects some differences between our decoding and memory-less Bayesian decoding, especially near transitions between dynamic regimes. The purpose of the residual

analysis is to verify that the difference is modest, not a large mismatch between the model and the spike-based position evidence.

The reviewer also suggests a more flexible model in which the HMM could transition between states of the same dynamic type but with different parameters. As discussed above, this is a natural future extension, but in the present submission we kept the event-level parameterization simple to avoid unstable or non-identifiable estimates from short SWR events. We would like to acknowledge that more flexible approaches of this kind may become useful in the future if one can record much larger neural populations from the hippocampus (e.g., thousands of place cells simultaneously) with increased statistical power. We have added a discussion on this in the paper. Here is the updated relevant text:

"*Second, another natural extension would be a switching drift-diffusion model that switches between multiple drift-diffusion processes with distinct parameters or other types of dynamics (such as Levy flight). However, practically the limited amount of data available per SWR event may prevent one from estimating the parameters reliably. In this paper, we therefore adopt a more parsimonious model by explicitly including two stereotyped dynamics (i.e., stationary and uniform sampling) alongside a single drift-diffusion component. This choice preserves tractability while maintaining flexibility to capture the spatiotemporal heterogeneity of individual SWRs. Crucially, by allowing stationary and uniform regimes in the model, the method can isolate SWR segments with substantial drift and spatial coherence. Future experiments with much larger numbers of neurons recorded simultaneously from the hippocampus may enable reliable inference of parameters from these even more sophisticated models.*"

Minor.

Other than these concerns, the paper is well-written and clear, and I have no concerns about methodology.

Response: Thank you for this positive evaluation and your suggestions for further improving our work.

Reviewer #3:

The manuscript by Wu and Wei outlines a novel approach to characterizing hippocampal replay. The existing literature consists of several different approaches that have led to confusion and differing points of view in the field. This study introduces a physics-inspired method to characterize candidate replays by drift and diffusion parameters and applies it to existing data to extract some important insights. I believe this represents an important contribution to the field based on the intuitive reasoning and meaningful results it provides. Nevertheless, there are a few areas in which the manuscript could be significantly improved.

Major

1. The study notes that "12.2% of events can be classified as drift-diffusion...." A key aspect that could affect the findings is how events are identified. In this study, ripple band power is used to identify candidate events, but this depends largely on electrode placement and ripple detection, which can be variable. Therefore many other studies rely on the detection of "population bursts" or "high-frequency events" using multi-unit activities that appear to be more reliable. Many replay studies also implement additional inclusion/exclusion criterion, such as ripple frequency power, or a threshold on the number of active cells. It's unclear whether and how such choices could affect the results? I would recommend that the authors examine how their replay percentages and other results vary based on other criteria that are commonly used in the literature.

Response:

Thank you for these suggestions.

The reviewer points out that the event-detection criterion may influence the estimated fraction of events assigned to different replay dynamics. This is a valid concern. Following the original analysis of this dataset (Pfeiffer and Foster, 2015), we have used ripple-band power for the initial candidate detection. We agree with the reviewer that it is important to examine the impact of the event-detection criterion. To address this concern, we have revised the manuscript to add a new event-detection control analysis comparing the LFP-defined SWR candidates with events detected from multi-unit population activity (Fig. R1). In this analysis, we detected population-burst events by summing spikes across cells in each 10 ms bin, smoothing the population activity with the same 12.5 ms Gaussian kernel used for ripple-band detection, z-scoring the smoothed activity within session, and defining MUA events as intervals crossing 3 standard deviations above the session mean. Event boundaries were extended to the surrounding mean crossings, and the same duration and run-speed criteria were then applied. We first compared these MUA-defined events with the LFP-defined SWR candidates. After the event-detection step used for our spike-train analyses, 74.5% of the LFP-defined candidate events were also 3-SD MUA events. For the matched events, the temporal overlap was substantial: the median intersection-over-union (IoU) between matched LFP and MUA event intervals was 0.67. This indicates that the majority of events entering our analysis are also supported by strong population-burst activity.

Motivated by this comparison, we further repeated our main analyses on a more stringent subset of events: LFP-defined SWR candidates that also overlapped with a 3-SD MUA population-burst event. The resulting dynamic composition (Fig. R2) and replay-speed (Fig. R3) analyses were consistent with the results from the full event set. The fraction of events containing drift-diffusion dynamics was very similar in the full and MUA-matched datasets for pre-run sleep (9.82% vs. 9.99%), awake rest (49.72% vs. 55.05%), and post-run sleep (18.05% vs. 20.34%). The replay-speed estimates were also similar: mean drift-diffusion replay speed was 4.40 vs. 4.37 m/s in pre-run sleep, 4.68 vs. 5.13 m/s in awake rest, and 4.00 vs. 4.12 m/s in post-run sleep for the full and MUA-matched datasets, respectively.

In addition, we would like to clarify that the original event-selection procedure already included a population-activity criterion after ripple-band detection. Specifically, after LFP-defined SWR candidates were binned onto 10 ms spike-counts, the event window used for downstream spike-train analyses was restricted to the contiguous population-burst interval spanning from the first to the last bin whose mean population

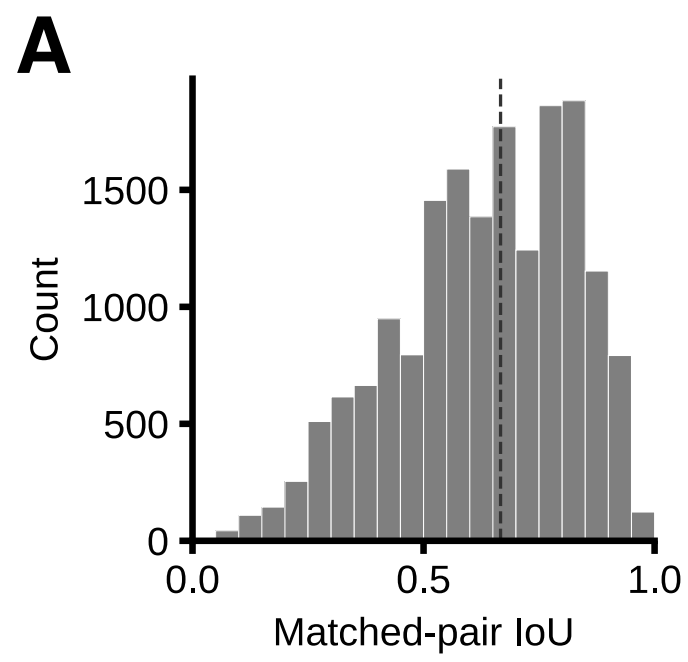

Figure R1: Temporal overlap between the candidate SWRs analyzed in the manuscript and population bursts detected from multiunit activity (MUA). Candidate SWRs were detected by band-pass filtering the LFP at 150-250 Hz, extracting and Gaussian-smoothing the amplitude envelope (σ = 12.5 ms), and identifying excursions exceeding 3 SD above the mean, with event boundaries extended to the mean. Events lasting 50–2000 ms were retained, and events occurring during run epochs were restricted to mean speeds below 5 cm/s. Candidate boundaries were subsequently trimmed to the first and last 10 ms bins with a mean firing rate greater than 2 Hz per neuron, and trimmed events shorter than 50 ms were excluded. MUA population bursts were detected from the total spike count across neurons using the same smoothing, threshold, boundary, duration, and speed criteria. Temporally overlapping candidate SWRs and MUA bursts were matched one-to-one. For each matched pair, intersection over union (IoU) is the duration of overlap between the two events, divided by the duration from the earliest start to the latest end. An IoU of 1 indicates identical boundaries. The histogram pools 17,339 matched pairs from 23,272 candidate SWRs across N = 9 sessions. The dashed vertical line indicates the median IoU of 0.67.

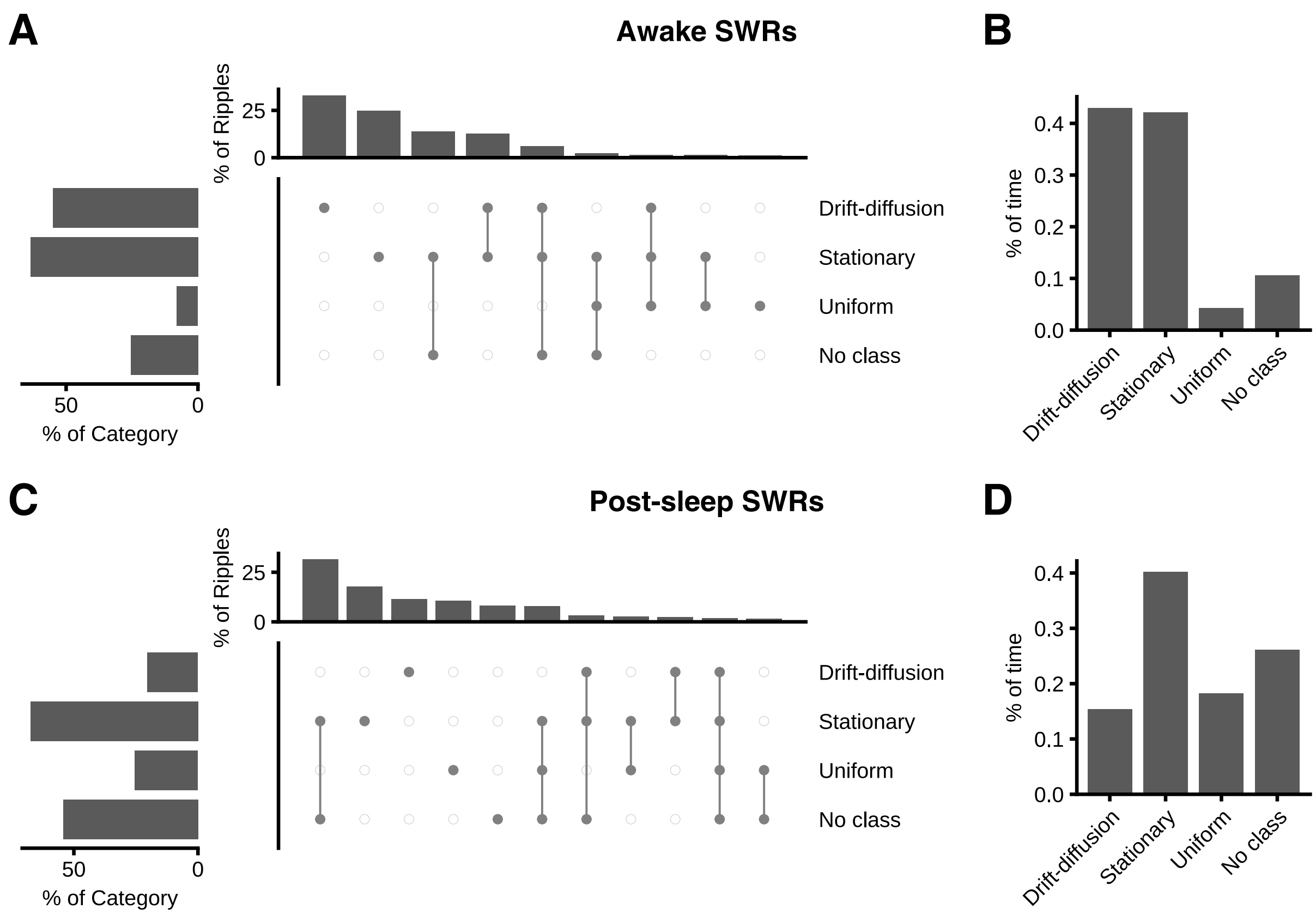

Figure R2: Composition of inferred spatiotemporal dynamics in the subset of candidate SWRs matched to MUA population bursts. The analysis and plotting conventions are the same as in Fig. 4, but are restricted to candidate SWRs that were temporally matched one-to-one with an independently detected MUA population burst. **A–B**. Dynamic-class combinations and fractions of classified time bins for awake SWRs. **C–D**. Corresponding results for post-sleep SWRs. Events were pooled across N = 9 sessions.

firing rate exceeded 2 spikes/second/neuron, and the resulting interval was required to last at least 50 ms. Therefore, ripple-band power was used to identify the initial candidate intervals, and we further constrained

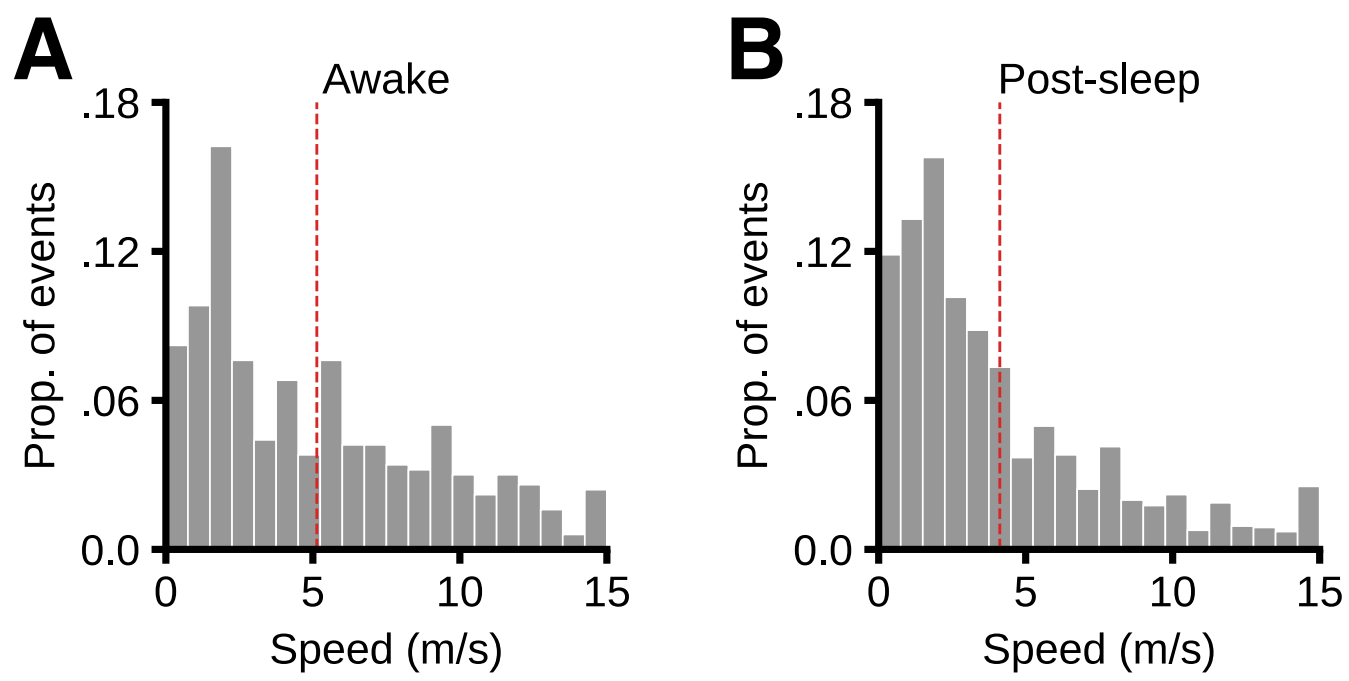

Figure R3: Replay-speed distributions in the subset of drift-diffusion events matched to MUA population bursts. The analysis and plotting conventions are the same as in Fig. 6D,E, but are restricted to candidate SWRs that were temporally matched one-to-one with an independently detected MUA population burst and classified as drift-diffusion events. **A**. Replay speeds for awake events. **B**. Replay speeds for post-sleep events. Red dashed lines indicate the distribution means (awake, 5.134 m/s; post-sleep, 4.120 m/s).

the events analyzed in this study by population activity.

Overall, the new analysis presented above now shows that the results remain consistent when a more stringent population-burst criterion is applied. Thank you for raising this concern.

2. The study analyzes so-called stationary events and compares these with shuffles. However, this is done without regards to the locations that are decoded during these events. In previous studies, stationary events were found to encode the animals current location, during immobility. It's not clear whether this is also the case among shuffles in the current study. This may be an important distinction. It may also be that false-positive events (e.g. with spurious ripple power caused by chewing, etc.) are more likely to be stationary, which is another point that merits consideration. It would be helpful if the study provided example stationary events in the data as well as in the shuffles. In fact, the study should also provide more examples of other types of events, including uniform and "no class".

Response: Thank you for these comments.

The reviewer noted that our stationary-event analysis compared real data against shuffle controls but did not sufficiently address where the stationary positions were decoded in shuffles. The reviewer further points out that previous work reported stationary events encoding the animal's current position, asks whether similar decoded positions also appear in the shuffled data, and raises the possibility that some stationary classifications could arise from false-positive candidate events or other non-replay activity.

We have revised the text for Fig. 4 (composition of SWR dynamics) and Fig. 6 (replay speed) to make our interpretation of stationary periods more cautious. We emphasize that the raw fraction of SWR time assigned to stationary dynamics must be interpreted together with shuffle controls (Fig. 5). Across time-bin shuffle, neuron-identity shuffle, and place-field rotation shuffle, the fraction of events with stationary periods in real data is only modestly larger than the corresponding shuffled fractions. In particular, the fraction of SWRs with a substantial stationary period (> 50 ms total stationary duration) is 52.76% for awake events, compared with 55.65% for the time-bin shuffle, 43.93% for the neuron-identity shuffle, and 44.44% for the place-field rotation shuffle. Thus, awake SWRs do not significantly exceed the time-bin shuffle, although the real fraction exceeds the neuron-identity and place-field rotation shuffles by 8.83% and 8.32%, respectively. For post-sleep SWRs, the real fraction is 45.82%, compared with 38.31%, 41.84%, and 40.68% for the three controls, resulting in differences of 7.51%, 3.98% and 5.14%, respectively. Overall, these results suggest that only a small fraction of SWR events are *bona fide* stationary events.

To address the reviewer's question about decoded positions, we added a new analysis of the decoded positions during stationary-labeled time bins (Fig. S5 and Fig. S6). For each real SWR and for each

shuffle control, we used the same posterior-based classification criterion as in the main analysis to identify stationary time bins. We then computed the marginal posterior over position at those time bins and summarized the maximum a posteriori decoded position. This new analysis shows that decoded positions during stationary-labeled time bins can be spatially concentrated in both real data and shuffled data. In particular, in the time-bin shuffle and neuron-identity shuffle, where the original non-uniform place-field distribution remains part of the decoder, the decoded stationary positions remain concentrated at the two ends of the track. This supports the reviewer's point that the position distribution of stationary-labeled time bins may not by itself be taken as evidence for coordinated stationary replay. Instead, it indicates that stationary decoded positions can arise from multiple sources, including false positive SWRs and structure in the place-field ensemble. We have revised the Discussion to clarify possible sources of this phenomenon. Here is the relevant text:

"*This suggests that stationary dynamics observed in decoding may arise from multiple sources and by itself cannot be interpreted as conclusive evidence for strongly coordinated neural activity with slow dynamics. While the exact reasons for observing many stationary events in the shuffle data remain to be determined in future work, we hypothesize that the following two factors may contribute to it. First, the heterogeneity in the distribution of place fields may bias the decoded posterior, causing it to remain concentrated at the same spatial position across consecutive time bins. Second, if a small subset of neurons consistently exhibits high firing rates during segments of SWRs, stationary posterior distribution over position may arise in decoding*."

3. The second event (top row) in Figure 6A (& Fig S2) raises some questions. To me, visually this does not appear to be a "drift" replay. I would guess it would qualify as a "fragmented" replay using the Denovellis criterion. The study should do more to convince the readers why we should trust this to be a true drift replay rather than a misclassification by the algorithm.

Response: The reviewer pointed out that one of the example events previously shown as a drift-diffusion event did not visually appear to be a clear replay event. The reviewer therefore asks for stronger justification that events classified as drift-diffusion are not simply misclassified by the model. To address this concern, we have performed an additional analysis (reported in Fig. S2 of the revised manuscript), which quantifies the difference between our position decoding and memory-less Bayesian decoding. For the three types of dynamics considered in the model, the residual error (the difference in the decoded location between the two decoders) is much lower than the shuffle control and remains small, less than 15 cm on a 180 cm track at the 75% quantile. This analysis indicates that the switching-HMM decoding remains close to the position information supported directly by the instantaneous spike activity. We would also like to note that our model includes switching dynamics to account for temporal structure in the position posterior. Therefore, we expect some difference between our decoding and memory-less Bayesian decoding, and the residual analysis is intended to verify that this difference is modest, rather than a large mismatch between the model and the spike-based position evidence. In addition, the model-recovery analysis validates model identifiability by showing that the fitted model can recover the simulated dynamic structure and parameters when the ground truth is known. Together, the residual analysis and the recovery analysis would support the interpretation that the drift-diffusion classifications reflect reproducible position dynamics based on the spike data.

Regarding the specific example event noted by the reviewer, we agree that this event had a lower drift-diffusion posterior value than the other displayed drift-diffusion examples, although it still satisfied our event inclusion criterion for drift-diffusion classification. We would also like to emphasize that our main results are based on population-level statistics from a large number of SWR events. For clarity, we have replaced this example with an event that has stronger drift-diffusion posterior support. We have also revised the presentation of the figure so that the inferred dynamic posterior is accompanied by the spike raster and memory-less Bayesian decoder's posterior, making the basis for the classification more transparent.

4. The study also makes it a point to compare results from a study based on foraging in a 2D environment with results from rewarded behavior on a 1D linear track. This could be a bit misleading because the

animals' experienced trajectories are considerably different in the two cases, as is the geometry of the environment, which makes these comparisons problematic. One option to remedy this situation is for the authors to apply their methods to behavior in a 2D environment. This should be doable given that the Pfeiffer and Foster dataset used in this study contains many such 2D sessions.

Response:

Thank you for these suggestions.

The reviewer noted that comparisons between SWR dynamics observed after 2D open-field foraging and those observed on a rewarded 1D linear track could be ambiguous if the differences in environment geometry and behavioral experience are not stated clearly. To address this concern, we have revised the manuscript text to avoid making any direct comparison between our 1-D results and the 2-D results reported in Stella et al. (2019). For example, now we directly state: "*While our results are based only on data collected in 1-D environments and do not directly contradict the 2-D results reported in [31], they do motivate revisiting or further exploration of replay dynamics in 2-D and additional environment and behavioral settings.* "

The reviewer also suggests that applying the method to a 2D dataset would make the comparisons more precise. We agree that incorporating 2D datasets would be valuable, especially because one major prior study comparison involves sleep recordings after 2D open-field foraging. We have revised the Discussion section to further elaborate on the possibility of extending our model framework to 2D environments. We would also like to note that extending the model to 2D is feasible, but it requires additional model assumptions. In particular, the 2D setting requires decisions about how to parameterize drift-diffusion in a two-dimensional environment, how to represent movement direction and diffusion jointly, and how to sensibly detect jumping events within the same probabilistic framework. We are currently working on this extension and plan to report it in a future study.

5. Finally, I found the treatment of the pre-run SWRs to be rather confusing, and the study appears to make contradictory conclusions. They appear to find that the data is above chance only if "chance" is assessed using one of the several shuffle possibilities, yet they conclude that their data supports "preplay". But doesn't this potentially have more to do with the suitability of different shuffles than it does of pre-existing structure, as they allude to in the discussion? It seems an equally valid conclusion could be that their analysis does not support the existence of pre-existing temporal structure, unless the study can provide arguments or evidence for trusting one surrogate dataset more than the others. Please clarify.

Response:

Applogize for the confusion. We agree that this is an important point and that the interpretation of the results on pre-run SWRs should be stated more clearly. We have revised the text in the Results section and the corresponding figure to make this point clearer. In the revised manuscript, we present the three shuffle controls together in order to test different null hypotheses and to provide a fuller picture of how pre-run sleep SWRs differ from awake-rest and post-run sleep SWRs. As shown in Fig. 8, post-run SWRs show robust drift-diffusion structure relative to all three shuffle controls, whereas pre-run SWRs differ only from the time-bin shuffle and not from the neuron-identity or place-field rotation shuffles. We now state this result more directly in the Results section.

The reason for including the time-bin shuffle is that it is closely related to the analysis used in a recent study that reports evidence supporting pre-existing structure (Farooq et al., 2019). Thus, the time-bin shuffle result is relevant for comparison with prior work. At the same time, the neuron-identity and place-field rotation shuffles also provide stringent tests that disrupt the mapping between spikes and place-field order. Under these controls, we do not find significant evidence for preplay.

To avoid overstatement, we have revised the manuscript to emphasize that pre-existing temporal structure,

if present, is much weaker than replay after spatial experience. This conclusion is supported in two ways. First, pre-run SWRs exceed chance only under the time-bin shuffle, whereas awake-rest and post-run SWRs show stronger and more consistent drift-diffusion structure across shuffle controls. Second, after accounting for shuffles, the estimated fraction of *de novo* long drift-diffusion events is much smaller in pre-run sleep than after experience. Using the difference between real and shuffled drift-diffusion duration distributions, the fraction of pre-run SWRs with drift-diffusion duration longer than 100 ms is only 0.64%, compared with 16.07% for awake SWRs and 4.21% for post-run SWRs under the same time-bin shuffle comparison. Thus, our revised conclusion is that pre-run SWRs show weak, shuffle-dependent evidence for pre-existing temporal structure, while long replay sequences are rare. We have also revised the Discussion to state the corresponding caveat more clearly: evidence for pre-existing structure is detected only under particular analysis choices, and caution is warranted because time-bin shuffle may provide a weaker null distribution than neuron-identity or place-field rotation shuffle. The new text reads:

"*Third, regarding the question of the existence of pre-existing temporal structures before spatial experience in a given environment Dragoi and Tonegawa (2011), we only found weak evidence suggestive of sequential structure in pre-run sleep when comparing the data distribution to the null distribution generated through time-bin shuffle [47,50]. No sequential structure was detected based on two other shuffle methods (neuron shuffle and place-field shuffle). Furthermore, the results based on time-bin shuffle show that long drift-diffusion sequences that are comparable to classic replay events with a timescale of 100 ms are exceptionally rare (less than 1% of SWRs in pre-run sleep). Additional caution needs to be taken, because time-based shuffle may destroy the temporal structure in individual neurons (i.e., the firing rates of individual neurons become less smooth over time) and thus may provide a weaker null distribution and inflate the estimated sequential structure.* "

Minor

1. The claim of being "the most powerful way" in the Results section needs further explicit support.

Response: This is a good point. We have toned this down in the text.

2. What is the basis for choosing a threshold of 0.7 for the drift rate? Is this arbitrary?

Response: Thank you for this question. The basic rationale for the threshold is to require evidence substantially above chance. Because there are three dynamic categories, chance-level posterior mass for a category is 1/3 $\approx$ 0.33. Doubling this chance level gives 2/3 $\approx$ 0.66, which we rounded to 0.7 as a cutoff.

We would like to further clarify that this threshold is an inclusion criterion used in the analysis stage to convert the continuous marginal posterior probability of each dynamic state into a discrete label for each event time bin. Specifically, after model fitting, each time bin has a marginal posterior over the three dynamic categories, with probabilities in $[0, 1]$. The 0.7 threshold is then used to label a time bin as drift-diffusion, stationary, or uniform only when the corresponding marginal posterior probability is sufficiently high. Thus, this threshold would not have affected the maximum-likelihood parameter fitting but is relevant to the downstream classification and summary of inferred dynamics.

3. Please show some sample events for Fig S3B.

Response: The reviewer asks us to show sample events for the analysis in Fig. S3B. We would like to note that after revision this analysis corresponds to the current Fig. S3C. We have revised the analysis presented in Fig. S3 to compare the replay-speed distribution of drift-diffusion events against a zero-drift pure-diffusion null. For awake-rest and post-sleep drift-diffusion events, replay speed was estimated from the fitted drift rate. For the null distribution, we used the corresponding pure-diffusion model: for each drift-diffusion event, a zero-drift diffusion model was fit by maximum likelihood to estimate the diffusion parameter, and then 10 pure-diffusion trajectories with the same event duration were simulated. The speed of each simulated

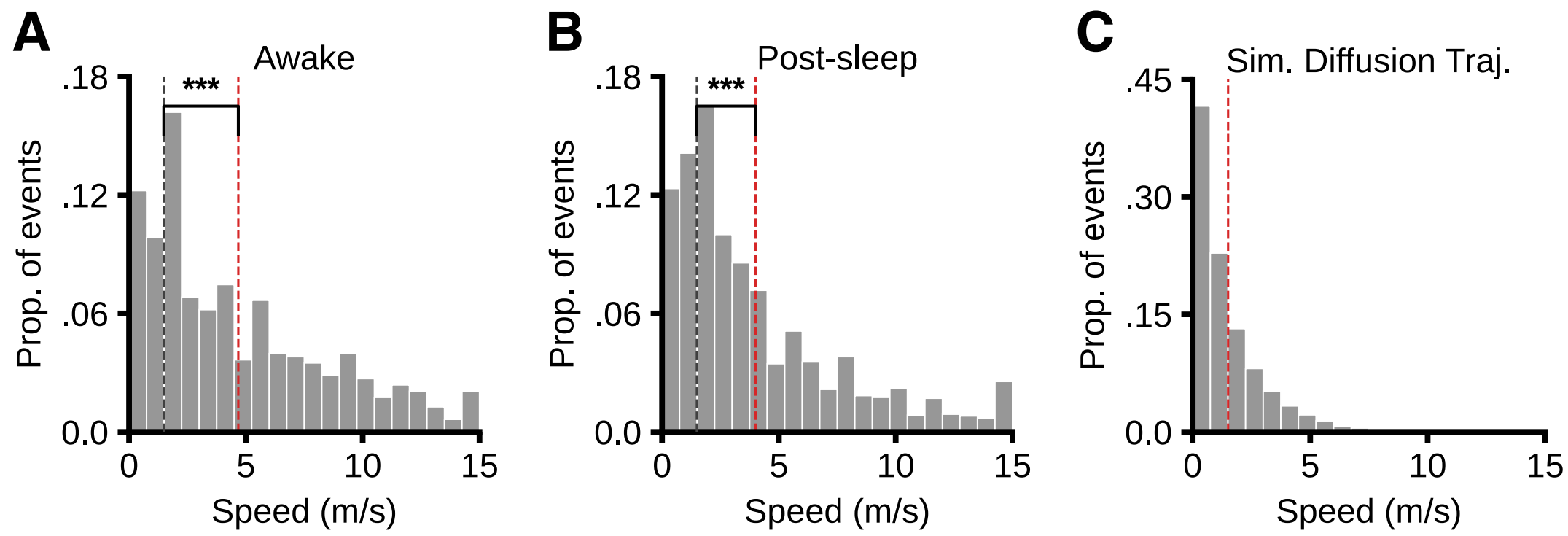

Figure S3: Replay-speed distributions are inconsistent with a pure-diffusion null. A–B. Histograms of replay speed for drift-diffusion events in awake and post-sleep periods. Drift-diffusion events were identified from the marginal posterior of dynamics using a posterior threshold of 0.7 sustained for at least five consecutive 10 ms bins. Replay speed was estimated from the fitted drift rate. **C.** Speed distribution under a zero-drift diffusion model. For every drift-diffusion event in **A–B**, a pure-diffusion model was fit by maximum likelihood to estimate the diffusion parameter. From the fitted pure-diffusion model, 10 trajectories with the same duration as the corresponding event were simulated. The speed of each simulated trajectory was defined as the absolute slope from a linear regression of position against time. Dashed red lines mark the mean of each distribution: 4.677 m/s (awake events), 4.002 m/s (post-sleep events) and 1.488 m/s (pure-diffusion trajectories). Dashed dark gray lines in **A–B** mark the pure-diffusion mean. Brackets indicate one-sided Welch two-sample tests comparing the mean replay speed in awake or post-sleep against the mean speed of simulated pure-diffusion trajectories, with the alternative hypothesis that replay speed is greater than the pure-diffusion speed. Asterisks denote statistical significance (***, $p < 0.001$; awake vs. pure diffusion, $p = 8.1 \times 10^{-74}$; post-sleep vs. pure diffusion, $p = 1.7 \times 10^{-191}$).

trajectory was defined as the absolute slope from a linear regression of position against time. Because this null analysis simulates latent position trajectories directly, there are no corresponding spike rasters, decoded posteriors, or event-level examples to plot in the same format as empirical SWR examples. For this reason, we summarize the pure-diffusion null with its trajectory speed distribution in Fig. S3C.

4. Extra comma in abstract "... that, only ..." "... or is even stationary?" doesn't make sense.

5. Missing "of" in "the existence stationary dynamics"

Response: We have fixed these. Thanks for pointing these out.

References

Eric L Denovellis, Anna K Gillespie, Michael E Coulter, Marielena Sosa, Jason E Chung, Uri T Eden, and Loren M Frank. Hippocampal replay of experience at real-world speeds. *Elife*, 10:e64505, 2021.

Xingsi Dong, Tianhao Chu, Tiejun Huang, Zilong Ji, and Si Wu. Noisy adaptation generates lévy flights in attractor neural networks. *Advances in neural information processing systems*, 34:16791–16804, 2021.

George Dragoi and Susumu Tonegawa. Preplay of future place cell sequences by hippocampal cellular assemblies. *Nature*, 469(7330):397–401, 2011.

Usman Farooq, Jeremie Sibille, Kefei Liu, and George Dragoi. Strengthened temporal coordination within pre-existing sequential cell assemblies supports trajectory replay. *Neuron*, 103(4):719–733, 2019.

Zilong Ji, Tianhao Chu, Xingsi Dong, Changmin Yu, Daniel Bush, Neil Burgess, and Si Wu. Dynamical modulation of hippocampal replay through firing rate adaptation. *Nature Communications*, 2026.

Emma L Krause and Jan Drugowitsch. A large majority of awake hippocampal sharp-wave ripples feature spatial trajectories with momentum. *Neuron*, 110(4):722–733, 2022.

Alexander Lex, Nils Gehlenborg, Hendrik Strobelt, Romain Vuillemot, and Hanspeter Pfister. Upset: visualization of intersecting sets. *IEEE transactions on visualization and computer graphics*, 20(12): 1983–1992, 2014.

Brad E Pfeiffer and David J Foster. Autoassociative dynamics in the generation of sequences of hippocampal place cells. *Science*, 349(6244):180–183, 2015.
