# Supplementary material for: Drift-diffusion dynamics of hippocampal replay
Source: PLoS Comput Biol. 2026 Sep 22;22(9):e1014761. doi: 10.1371/journal.pcbi.1014761 (PMC13614660; doi:10.1371/journal.pcbi.1014761)
Supplement: Supplementary file 9 [file pcbi.1014761.s024.pdf]

Response to the reviews

Summary

We thank the reviewers for their careful evaluation of our revised manuscript and for their constructive feedback. Reviewer 1 and Reviewer 2 were satisfied with the revised manuscript. Reviewer 3 still had a few additional concerns, which we now have addressed in a further revised manuscript by performing additional control analyses and clarifying the analysis procedures. The main changes are summarized below, followed by figures and detailed point-by-point responses.

1. We have analyzed events detected using a standard multiunit-activity (MUA) criterion without requiring ripple-band activity in the LFP. We applied the same switching-HMM and dynamics-classification pipeline to all 38,935 pure-MUA events identified across nine recording sessions. The qualitative relationships among pre-sleep, awake, and post-sleep dynamics were consistent with the manuscript results. The pure-MUA analysis contained a lower fraction of drift-diffusion dynamics, particularly during awake rest, suggesting that the broader criterion includes additional population bursts with weaker sequential spatial structure. We retain the LFP-based selection for the primary analysis and present the pure-MUA result as a complementary robustness analysis in the supplemental information.

2. We have further strengthened the presentation of the modeling framework. We show a broad set of real events with drift-diffusion, stationary, uniform, and “No Class” dynamics, together with drift-diffusion fits to all three types of shuffle surrogates. We have also expanded the Methods description of the shuffle controls.

In making these changes, we feel that the manuscript and its supporting response materials have been further improved. We thank the reviewers again for their thoughtful comments and valuable suggestions.

Point-by-point response

Reviewer #1: The authors have addressed my concerns in their revised manuscript.

Response: Thank you for your careful evaluation and the constructive feedback on our manuscript during the review process.

Reviewer #2: The authors have made a thorough set of responses to the original critiques.

Response: Thank you for your suggestions and critiques on our manuscript.

Reviewer #3: The manuscript by Wu and Wei has been substantially improved. However, in several areas, my concerns were only partially addressed, and it seems some of my original comments may have been missed or misunderstood.

1. I previously recommended examining how the results would vary if standard detection criteria were used. Instead of doing this, the authors applied even more restrictive criteria to their event detection, which misses the point. As Reviewer 1 also noted, not all replay events occur during sharp-wave ripples (SWRs). The core concern is that the study needs to show how the results would look when using the standard methods applied in other papers, which typically use just MUA, without ripples in the LFP. This remains unaddressed.

Response: Thank you for clarifying this concern. To directly address this point, we have now detected population-burst events using MUA alone as suggested, without requiring ripple detection in the LFP.

We summed spikes across neurons in non-overlapping 10 ms bins, smoothed the resulting population activity with a Gaussian kernel (σ = 12.5 ms), and z-scored the smoothed activity within each recording session. MUA events were defined as excursions exceeding 3 SD above the session mean, with event boundaries extended to the surrounding mean crossings. We retained events lasting 50–2000 ms and, for events occurring during run epochs, required a mean running speed below 5 cm/s.

This procedure identified 38,935 pure-MUA events across nine recording sessions, more than the 23,272 events considered in the manuscript. The numbers of events detected only by the manuscript procedure, matched between procedures, or detected only from MUA were:

Epoch	Manuscript only	Matched	Pure MUA only
Pre-sleep	1,301	5,448	5,961
Awake	393	1,019	4,418
Post-sleep	4,239	10,872	11,217
Total	5,933	17,339	21,596

We then applied the same switching-HMM fitting and dynamics-classification pipeline to all pure-MUA events (Fig. S3). Qualitatively, the main results were consistent with those obtained from the events considered in the manuscript: stationary dynamics occupied the largest fraction of time in every epoch, and awake events exhibited more drift-diffusion dynamics than pre- or post-sleep events. At the event level, the fractions of pure-MUA events containing at least one drift-diffusion segment were 10.78%, 38.27%, and 16.46% for pre-sleep, awake, and post-sleep, respectively, compared with 9.82%, 49.72%, and 18.05% for the manuscript events. At the time-bin level, drift-diffusion accounted for 5.81%, 28.12%, and 10.27% of pure-MUA event time, compared with 5.83%, 39.53%, and 13.46% for the manuscript events.

One quantitative difference was the lower fraction of drift-diffusion dynamics in the pure-MUA analysis during awake rest, with a smaller reduction during post-sleep and nearly unchanged results during pre-sleep. This suggests that the broader MUA-only criterion admits additional population bursts with weaker sequential spatial structure. Nevertheless, the qualitative relationships across brain states and the overall composition of dynamics remained consistent with the manuscript results.

For the primary analyses, we retain the LFP-based event-selection procedure to remain consistent with the original analysis of this dataset (Pfeiffer and Foster, 2015). The pure-MUA analysis above provides a complementary control analysis showing events detected independently of ripple-band LFP activity.

We have incorporated these results into the revised version of the paper by adding a new figure to the SI (Fig. S3), a new subsection to the SI (Section A.3), and pointers to these results in the main text (pages 10-11) and in the Methods section.

2. The related overlap analysis was restricted only to the subset of events that were matched. This artificially inflates the median intersection-over-union (IoU) values because it completely ignores all the instances where IoU = 0 (no match), while still including instances where IoU = 1.

Response: We understand the reviewer's concern. The median IoU of 0.67 that we previously reported was calculated across the 17,339 matched event pairs. Therefore, it describes the similarity of event boundaries conditional on a match.

For the present concern, we would like to note that the model has been validated separately through parameter-recovery analyses using simulated data (Fig. 3) and a residual analysis on the experimental data comparing switching-HMM position estimates with memory-less Bayesian decoding (Fig. S2). More importantly, we have now applied the full analysis to all 38,935 pure-MUA events, including those without an LFP-based match. As shown in Fig. S3, the qualitative relationships among pre-sleep, awake, and post-sleep dynamics remain consistent with the manuscript results.

For the primary analyses, we retain the LFP-based event-selection procedure because it follows the original analysis of this dataset (Pfeiffer and Foster, 2015) and identifies a set with stronger sequential structure, as indicated by the larger fraction of drift-diffusion dynamics described above. We now present the pure-MUA analysis as a complementary robustness validation in the paper.

3. I previously requested more examples, including different types of real events as well as drift-diffusion fits to shuffle surrogates, but these were not provided. With only several hand-picked examples to go by (and the authors even removed one event that showed ambiguity), it is difficult to evaluate the true effectiveness of the technique.

Response: We thank the reviewer for clarifying this point and we understand the concern about the robustness of the method. We would like to mention that the robustness is evaluated through recovery validation and residual analysis. Our parameter-recovery analysis uses simulated events with known ground truth and shows that the switching-HMM can recover the dynamic regimes and drift-diffusion parameters under experimentally realistic event durations and neural-population sizes (Fig. 3). Our goodness-of-fit residual analysis further shows that switching-HMM position estimates remain close to memory-less Bayesian position estimates supported directly by the instantaneous spike activity (Fig. S2). These analyses test both recovery when the ground truth is known and consistency with the position information in the experimental spike data.

Regarding the example removed from the previous figure, that event met our classification rule but had lower drift-diffusion posterior support than the other displayed drift-diffusion examples. Thus, the model itself indicated weaker evidence for interpreting that event as drift-diffusion. We replaced it to avoid presenting an ambiguous event as a canonical example. We note that ambiguity in an individual event can arise from recording noise or finite sampling of the neural population, and may not by itself demonstrate a systematic bias of the method. We agree such events should be interpreted cautiously, while the recovery and residual analyses should provide quantitative validation under realistic experimental settings.

Moreover, we provide additional example events with drift-diffusion, stationary, uniform, and "No Class" dynamics in real SWRs (Fig. S4). We also provide drift-diffusion fits to surrogate events generated by each of the three shuffle procedures (Fig. R1).

4. The Methods section needs to explicitly describe the shuffles applied. For example, for the neuron-identity shuffle, it is unclear whether the shuffle was performed across all recorded neurons or just the subset active within a given event. I guess it is per-event based on Figures S5 and S6, but it is hard to be sure. Additionally, for Figure 5C, the text states that the place-field shift preserves field shape. I don't see how this is possible if a circular shift moves the field center to one of the edges—wouldn't that split the field into two halves on opposite ends?

Response: Thank you for these comments. We agree that it is helpful to explain the shuffle procedures in the Methods section. To clarify, for the neuron-identity shuffle, the shuffle was performed separately for each SWR. For each SWR and each shuffle repeat, we randomly permuted all neurons included in the analysis for that recording session, including neurons that may not spike during that particular SWR. The same permutation was applied across all time bins of the SWR, so that a neuron's complete within-event spike train was reassigned to another neuron identity while the place fields remained unchanged. For the place-field rotation shuffle, we circularly shifted each neuron's discretized place-field vector. This operation preserves the firing-rate values and their circular ordering, but a field that crosses a boundary of the linearized track wraps around and can therefore appear split between the two ends.

We have also revised the Methods section to add the following paragraph:

> ***Shuffle analysis***. *Null distributions were generated using three shuffle procedures, each repeated for 120 times. For the time-bin shuffle, the time bins of the spike-count raster were randomly permuted within each SWR using a single permutation shared across neurons. This preserved the population activity vector in each time bin and the total spike count of each neuron while disrupting temporal order. For the neuron-identity shuffle, all neuron columns included in the analysis for a recording session were randomly permuted within each SWR, including neurons that were silent during that event. A single permutation was applied across all time bins of the SWR, and an independent permutation was drawn for each SWR and shuffle repeat. For the place-field rotation shuffle, the spike rasters were left unchanged and each neuron's place field was independently circularly shifted by a uniformly sampled integer number of spatial bins. One shuffled place-field ensemble was generated for each repeat and used for all SWRs from that recording session. Each shuffled realization was fitted and analyzed using the same pipeline as the real data.*

5. It is currently unclear how stationary events and Brownian motion are expressed during pre-sleep. Figures 5 and 7 only look at awake and post-sleep periods; pre-sleep should be included for a complete picture.

Response: Thank you for this suggestion. We agree that adding pre-sleep panels to the analyses in Figs. 5 and 7 might provide a more complete comparison. However, after examining the pre-sleep results in greater detail, we believe that such extensions might imply a stronger interpretation of pre-sleep dynamics than is supported by the data.

Figure 8 provides our analysis of sequential structure during pre-sleep. The pre-sleep drift-diffusion structure exceeded the time-bin-shuffle control but did not differ significantly from either the neuron-identity or place-field rotation controls. Moreover, under the time-bin-shuffle comparison, only 0.64% of pre-sleep SWRs were estimated to contain drift-diffusion structure lasting longer than 100 ms. These results indicate that long sequential events are exceptionally rare before environment exposure and that the candidate pre-sleep drift-diffusion events may be false positives. Estimating replay speed or MSD/Brownian scaling after conditioning on this weakly supported subset could therefore produce quantitative results that are difficult to interpret biologically.

Furthermore, we would like to note that Figures 5 and 7 address the first two applications of the manuscript: stationary content and Brownian-motion-like scaling in replay after environment exposure. These analyses are designed for direct comparison between awake and post-sleep replay and for comparison with previous

literature on replay trajectories. In contrast, Fig. 8 treats pre-sleep activity as a separate and more tentative question about preplay, using the relevant shuffle controls.

For these reasons, we prefer to keep the current panels in Figs. 5 and 7. We believe that this avoids a possible over-interpretation of weakly supported pre-sleep drift-diffusion events while preserving the direct awake/post-sleep comparison.

6. Figure S7 is a nice addition because it examines mean-squared displacement (MSD) in stationary and uniform events. However, it is unclear why the "no-class" events were not also considered here.

Response: Thank you for this comment. “No Class” denotes time bins when the posterior probability does not meet the classification criterion for any of the three candidate regimes: drift-diffusion, stationary, or uniform. Events containing “No Class” bins do not share a single well-defined dynamical model and may comprise heterogeneous cases, such as ambiguous dynamics, transitions between regimes, or short-lived structures that do not meet the classification criterion. Thus, pooling these events as a separate group might not provide a clearly interpretable regime-specific MSD comparison.

Minor

1. Figure 2E doesn’t look right. Why is the posterior probability distribution whited out?

Response: Thank you for raising this point. The posterior heatmaps in Fig. 2 use the Python Matplotlib colormap `"hot"` with `vmin=0.0` and `vmax=0.2`. Under this color scale, values near 0 are black, intermediate values are dark red, and values at 0.2 are white. Thus, the white bins in panel E indicate that the posterior probability reaches 0.2.

References

Brad E Pfeiffer and David J Foster. Autoassociative dynamics in the generation of sequences of hippocampal place cells. *Science*, 349(6244):180–183, 2015.

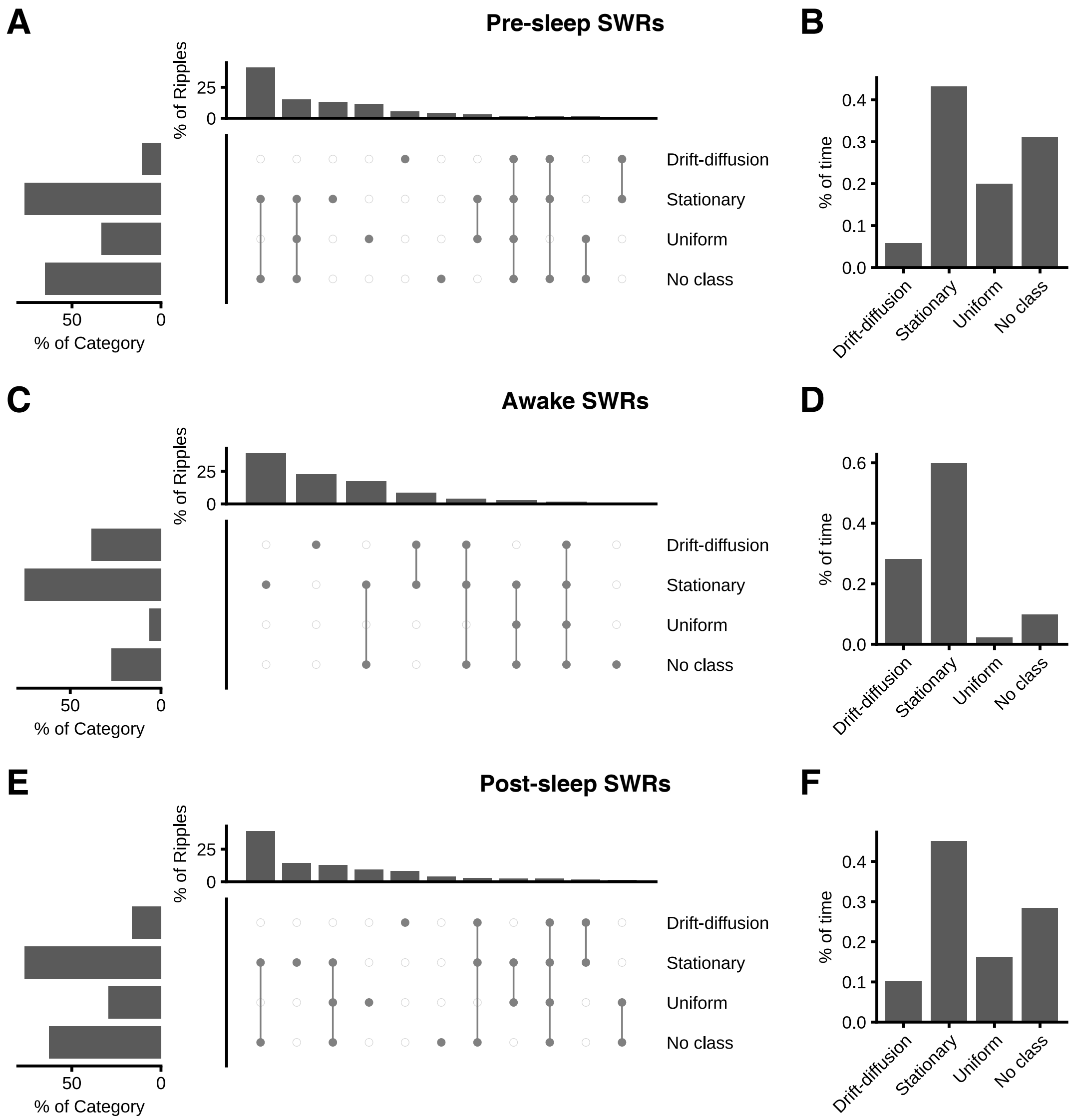

Figure S3: Composition of inferred spatiotemporal dynamics in events detected from multiunit activity alone. Population-burst events were detected independently of the LFP by summing spikes across neurons in 10 ms bins, smoothing the population activity with a Gaussian kernel (σ=12.5 ms), and identifying excursions exceeding 3 SD above the session mean, with event boundaries extended to the surrounding mean crossings. Events lasting 50–2000 ms were retained, and events during run epochs were restricted to mean speeds below 5 cm/s. The same switching-HMM and dynamics-classification procedure used for the manuscript events was applied to these pure-MUA events. For event-level summaries, a dynamic class was counted as present only when it persisted for at least two consecutive 10 ms bins. **A,C,E**. UpSet-plot summaries of dynamic-class combinations in pre-sleep, awake, and post-sleep events, respectively. Filled dots mark classes present in each combination, connected filled dots indicate co-occurring classes within the same event, and open dots mark absent classes. The left horizontal bars show the percentage of events containing each class. **B,D,F**. Fractions of event time bins assigned to each dynamic class for the corresponding epochs. Only class combinations occurring in more than 1% of events are shown. Events were pooled across N=9 recording sessions (pre-sleep, n=11,409; awake, n=5,437; post-sleep, n=22,089).

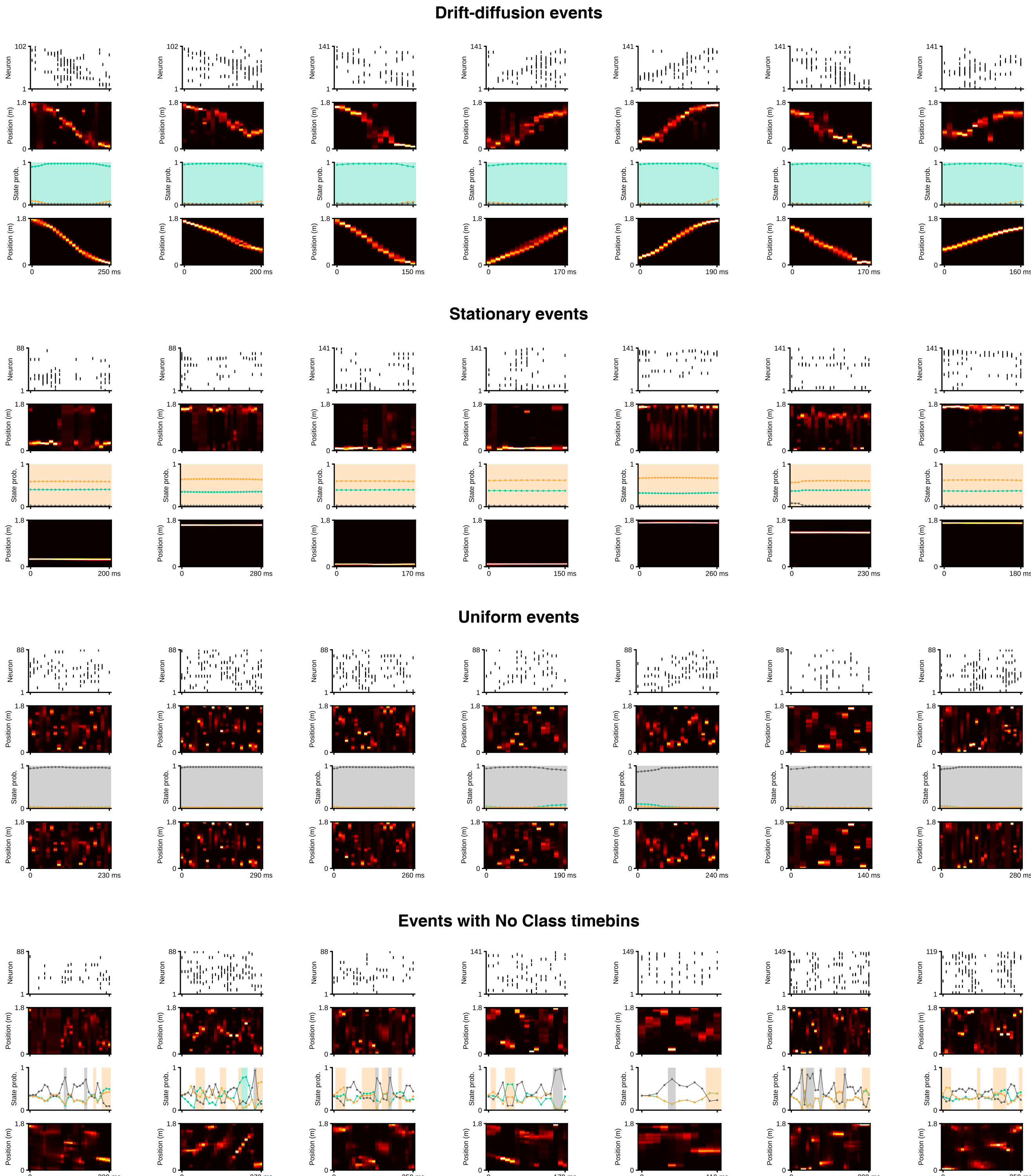

Figure S4: Additional examples of inferred spatiotemporal dynamics in real SWR events. Rows show drift-diffusion events, stationary events, uniform events, and events containing "No Class" time bins; columns show individual examples. For each event, panels from top to bottom show the spike raster, memory-less Bayesian position posterior, posterior probabilities of the three dynamic regimes, and switching-HMM position posterior. In the dynamic-probability panels, green, orange, and gray curves denote drift-diffusion, stationary, and uniform probabilities, respectively; background shading in the same colors indicates the assigned regime, and unshaded intervals indicate "No Class." In the position-posterior heat maps, brighter colors indicate higher posterior probability.

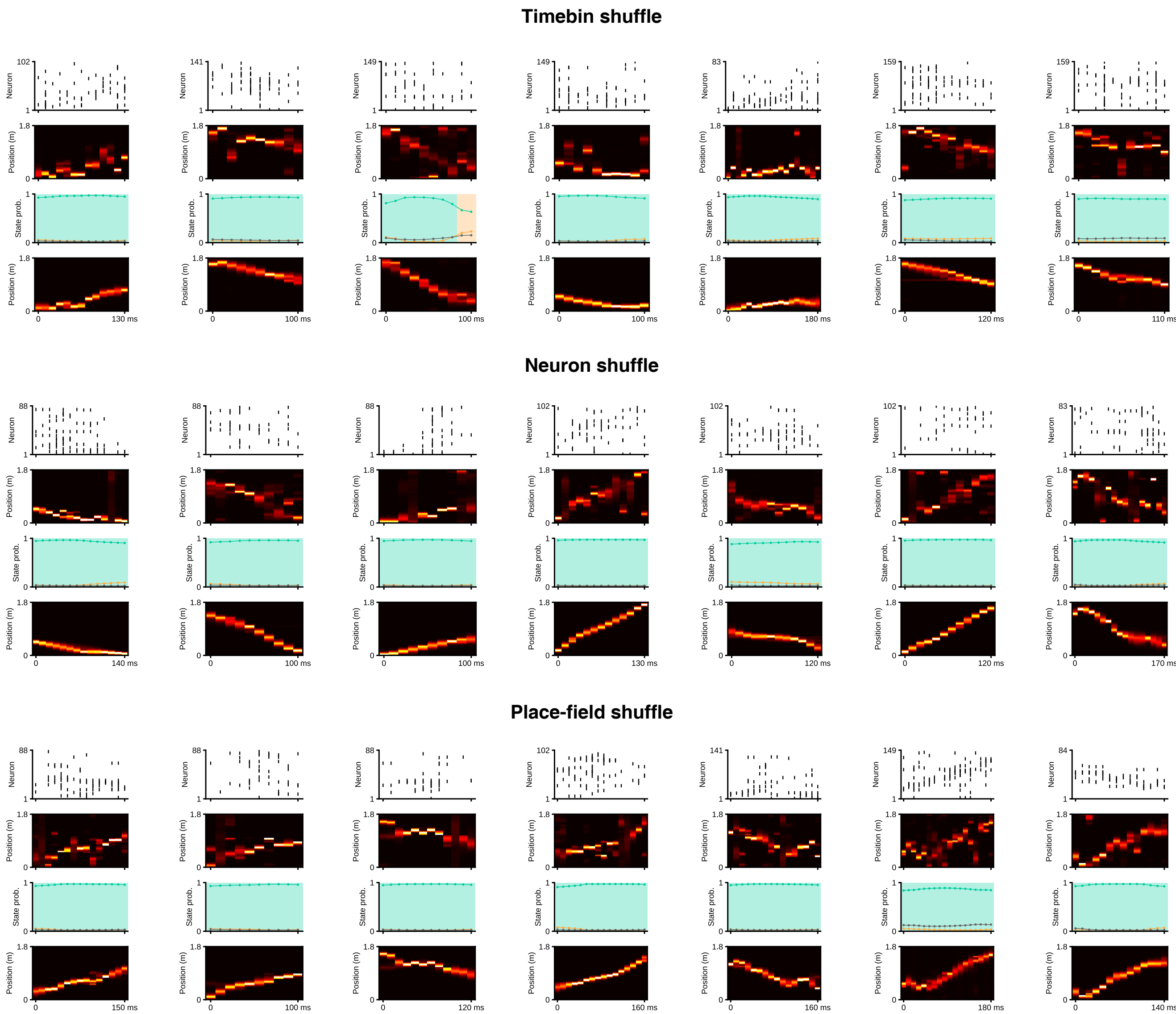

Figure R1: Examples of drift-diffusion fits to shuffled SWR surrogates. Row blocks show examples from the time-bin, neuron-identity, and place-field rotation shuffles; columns show individual surrogate examples. For each surrogate, panels from top to bottom show the spike raster analyzed for that surrogate, memory-less Bayesian position posterior, posterior probabilities of the three dynamic regimes, and switching-HMM position posterior. In the dynamic-probability panels, green, orange, and gray curves denote drift-diffusion, stationary, and uniform probabilities, respectively; background shading in the same colors indicates the assigned regime, and unshaded intervals indicate “No Class.” In the position-posterior heat maps, brighter colors indicate higher posterior probability.
