# Supplementary material for: Drift-diffusion dynamics of hippocampal replay
Source: PLoS Comput Biol. 2026 Sep 22;22(9):e1014761. doi: 10.1371/journal.pcbi.1014761 (PMC13614660; doi:10.1371/journal.pcbi.1014761)
Supplement: Peer review history

# Peer review: Decision Letter 0

**DOI:** 10.1371/journal.pcbi.1014761.r001

**Authors:** Thomas Serre, Matthias Helge Hennig

19 Apr 2026

Drift-diffusion dynamics of hippocampal replay

PLOS Computational Biology

Dear Dr. Wei,

Thank you for submitting your manuscript to PLOS Computational Biology. After careful consideration, we feel that it has merit but does not fully meet PLOS Computational Biology's publication criteria as it currently stands. Therefore, we invite you to submit a revised version of the manuscript that addresses the points raised during the review process.

Please submit your revised manuscript by Jun 19 2026 11:59PM. If you will need more time than this to complete your revisions, please reply to this message or contact the journal office at ploscompbiol@plos.org. When you're ready to submit your revision, log on to https://www.editorialmanager.com/pcompbiol/ and select the 'Submissions Needing Revision' folder to locate your manuscript file.

Please include the following items when submitting your revised manuscript:

* A letter that responds to each point raised by the editor and reviewer(s). You should upload this letter as a separate file labeled 'Response to Reviewers'. This file does not need to include responses to formatting updates and technical items listed in the 'Journal Requirements' section below.

* A marked-up copy of your manuscript that highlights changes made to the original version. You should upload this as a separate file labeled 'Revised Manuscript with Track Changes'.

* An unmarked version of your revised paper without tracked changes. You should upload this as a separate file labeled 'Manuscript'.

If you would like to make changes to your financial disclosure, competing interests statement, or data availability statement, please make these updates within the submission form at the time of resubmission. Guidelines for resubmitting your figure files are available below the reviewer comments at the end of this letter

We look forward to receiving your revised manuscript.

Kind regards,

Matthias Helge Hennig, Ph.D.

Academic Editor

PLOS Computational Biology

Thomas Serre

Section Editor

PLOS Computational Biology

**Journal Requirements:**

1) We ask that a manuscript source file is provided at Revision. Please upload your manuscript file as a .doc, .docx, .rtf or .tex. If you are providing a .tex file, please upload it under the item type u2018LaTeX Source Fileu2019 and leave your .pdf version as the item type u2018Manuscriptu2019.

2) Please provide an Author Summary. This should appear in your manuscript between the Abstract (if applicable) and the Introduction, and should be 150-200 words long. The aim should be to make your findings accessible to a wide audience that includes both scientists and non-scientists. Sample summaries can be found on our website under Submission Guidelines:

https://journals.plos.org/ploscompbiol/s/submission-guidelines#loc-parts-of-a-submission

3) Please upload all main figures as separate Figure files in .tif or .eps format. For more information about how to convert and format your figure files please see our guidelines:

https://journals.plos.org/ploscompbiol/s/figures

4) We have noticed that you have uploaded Supporting Information files, but you have not included a list of legends. Please add a full list of legends for your Supporting Information files after the references list.

5) Some material included in your submission may be copyrighted. According to PLOS’s copyright policy, authors who use figures or other material (e.g., graphics, clipart, maps) from another author or copyright holder must demonstrate or obtain permission to publish this material under the Creative Commons Attribution 4.0 International (CC BY 4.0) License used by PLOS journals. Please closely review the details of PLOS’s copyright requirements here: PLOS Licenses and Copyright. If you need to request permissions from a copyright holder, you may use PLOS's Copyright Content Permission form.

Please respond directly to this email and provide any known details concerning your material's license terms and permissions required for reuse, even if you have not yet obtained copyright permissions or are unsure of your material's copyright compatibility. Once you have responded and addressed all other outstanding technical requirements, you may resubmit your manuscript within Editorial Manager.

Potential Copyright Issues:

Figure 1A. Please confirm whether you drew the images / clip-art within the figure panels by hand. If you did not draw the images, please provide (a) a link to the source of the images or icons and their license / terms of use; or (b) written permission from the copyright holder to publish the images or icons under our CC BY 4.0 license. Alternatively, you may replace the images with open source alternatives. See these open source resources you may use to replace images / clip-art:

- https://commons.wikimedia.org

- https://openclipart.org/".

6) Please provide a completed 'Competing Interests' statement, including any COIs declared by your co-authors. If you have no competing interests to declare, please state "The authors have declared that no competing interests exist". Otherwise please declare all competing interests beginning with the statement "I have read the journal's policy and the authors of this manuscript have the following competing interests:".

Note: If the reviewer comments include a recommendation to cite specific previously published works, please review and evaluate these publications to determine whether they are relevant and should be cited. There is no requirement to cite these works unless the editor has indicated otherwise.

**Reviewers' comments:**

Reviewer's Responses to Questions

Reviewer #1: The manuscript presents a switching hidden Markov model that characterizes hippocampal population dynamics during sharp-wave ripples as a mixture of drift-diffusion, stationary, and uniform regimes. Applied to the Pfeiffer & Foster (2015) dataset, they find that ~20% of SWRs exhibit drift-diffusion dynamics at speeds nearly an order of magnitude faster than running, that MSD scaling is quadratic (inconsistent with pure Brownian motion), and that preplay is detectable only under the weakest shuffle control. The modeling framework is well-motivated and the parameter recovery analyses are solid. The switching HMM framework is a methodological advance over traditional two-step replay detection; by jointly inferring dynamics and positions in a single model, it avoids the problems of first committing to a decoded trajectory and then testing it against a template. The drift and diffusion parameters are interpretable and map directly onto biologically meaningful quantities (replay speed and trajectory coherence). However, several issues, most critically, the pooling of sleep and awake SWRs and the lack of controls for non-spatial activity in the decoder, limit the interpretability of the key results and weaken the paper's claims to resolve discrepancies with prior work.

Major Points

1. Awake and sleep SWRs are pooled without justification or separate analysis

The main analyses (Applications 1 and 2) pool awake immobility and post-run sleep SWRs, a fact stated only in the Figure 5 caption and not discussed in the text. This is a significant problem because the prior work the authors compare against was brain-state specific: Stella et al. [31] analyzed SWRs during post-exploration rest/sleep in a separate rest box after 2D open-field foraging (finding Brownian-like dynamics), and Denovellis et al. [25] analyzed awake SWRs on linear tracks (finding real-world speeds). Sleep and awake replay are known to differ in temporal compression, sequential structure, and content (Joo & Frank, 2018, Nat Rev Neurosci). The authors claim their results "clarify" or "resolve" these prior discrepancies, but without separating brain states, they cannot determine whether the discrepancy reflects genuine methodological differences or simply the mixing of two populations with different dynamics. The authors should present the key analyses (speed distributions (Fig. 6), stationary fractions (Fig. 5), and MSD scaling (Fig. 7)) separately for awake and sleep SWRs. This is feasible with the existing dataset. At minimum, the brain state composition of the analyzed events should be clearly stated in the results text.

2. The decoder assumes all SWR activity encodes a position on the track

The model assumes that at every time bin during an SWR, the hippocampal population is encoding some position on the current track. However, a substantial fraction of SWR activity may not encode any spatial position. It may represent a different environment, non-spatial information, or simply lack coherent spatial content. When such activity is forced through the Bayesian decoder, it will produce posteriors biased toward positions with high place field density, likely classified as "stationary" or "uniform." This means these categories may be substantially inflated by non-spatial activity rather than reflecting genuine spatial dynamics. Figure S4 in the SI provides direct evidence for this concern: decoded positions during stationary periods cluster heavily at the track ends (0 and 1.8 m), exactly where reward wells are located and place field density is likely non-uniform.

I would suggest the authors test this with a control analysis. For example, decoding SWR activity using place fields from a different recording session or environment, or through simulation. This would characterize the model's false positive profile when activity genuinely does not encode positions on the current track. Understanding this baseline is essential for interpreting the ~80% of SWRs that lack drift-diffusion dynamics. If possible, it would be a significant improvement to the method to include a "not-on-track" state, though I recognize that this is not a straightforward addition to the modeling framework.

3. The high false positive rate for stationary classification needs more rigorous treatment

The shuffle analyses (Fig. 5) show that shuffled (temporally meaningless) data produces nearly as many "stationary" classifications as real data (the difference is only ~5%). The authors note this finding but do not adequately address its implications. Several issues:

(a) The "no class" category is never discussed in the main text. Figure S6B shows that for pre-run SWRs, "no class" accounts for ~27% of total SWR time, comparable to stationary and uniform. This is a substantial fraction that deserves discussion. The authors should report the "no class" prevalence for both pre- and post-run conditions and explain what this category represents.

(b) The discussion (p. 21) hypothesizes two reasons for false stationary detection (heterogeneous place field sampling and high-firing-rate neuron subsets) but tests neither, despite Figure S4 providing relevant evidence for the former. These hypotheses should be tested with the current data. One possible solution is replacing the uniform distribution with a fitted stationary distribution that formalizes the null hypothesis of non-uniform but random sampling in the data.

(c) The conceptual distinction between "uniform" and "no class" is unclear. If activity has no spatial structure, would the model assign it to "uniform" or "no class"? This needs clarification.

4. Claims about resolving discrepancies with prior work are confounded by multiple uncontrolled variables.

he paper frames its contribution partly as resolving discrepancies between Stella et al. [31] (Brownian motion, post-exploration rest after 2D foraging) and Denovellis et al. [25] (real-world speeds in awake). However, this study differs from both on multiple axes simultaneously: environment dimensionality (1D vs. 2D), behavioral task (back-and-forth running vs. open-field foraging), brain state (pooled awake+sleep vs. state-specific), recording context (on-track immobility vs. separate rest box), and analysis method. Without controlling for these variables, the source of any discrepancy is unidentifiable, yet the abstract claims the approach provides "unambiguous interpretations" and the discussion claims to "resolve" or "clarify" prior inconsistencies.

The authors should: (a) explicitly enumerate the variables that differ between their study and the prior work they compare against, (b) temper claims about resolving discrepancies accordingly, particularly in the abstract and introduction, and (c) separate awake and sleep analyses (see Major Point 1), which would at least control for one of these confounds. Applying the method to a 2D dataset (e.g., the Csicsvari lab data used by Stella et al., if publicly available) would substantially strengthen the paper, but at minimum the claims should be qualified to reflect the specific experimental context: a single 1D linear track dataset with pooled brain states.

Minor Points

1. The paper conflates SWRs and replay throughout (e.g., "dynamics of replayed positions z during putative replays (population activity during SWRs)."). These are distinct phenomena: many SWRs do not contain replay (as the paper itself shows, only ~20% exhibit drift-diffusion dynamics), and there are many replay events that occur not during SWRs (Widloski & Foster, 2025, Nat Comms). The authors should use more careful terminology, distinguishing between SWR events (the electrophysiological phenomenon) and replay events (the subset with sequential spatial content).

2 The drift-diffusion framework is a specific parametric assumption. Replay trajectories could exhibit dynamics that this model cannot capture. For example, Lévy flight-like jumps between distant locations, acceleration/deceleration within events, or non-stationary drift rates. Recent modeling work has shown that continuous attractor networks with firing rate adaptation can generate replay dynamics spanning stationary, Brownian-diffusive, and super-diffusive regimes (Dong, Chu, Huang, Ji & Wu, NeurIPS 2021; Ji et al., Nat Comms 2026). The paper validates parameter recovery from simulated data (Fig. 3), but does not assess goodness of fit to real data. A goodness-of-fit analysis (e.g., comparing likelihood to a more flexible nonparametric model, or examining residuals) would strengthen confidence that drift-diffusion captures the relevant structure in replay dynamics.

3. The preplay analysis compares pre-run SWRs only to post-run sleep replay. Since awake replay is generally more structured, this comparison may make preplay look relatively stronger than it would against the most stringent comparator. The authors should note this. Additionally, the prior preplay literature (e.g., Dragoi & Tonegawa 2011; Farooq et al. 2019) could be discussed more thoroughly to contextualize where these results land, and it would be good to confirm that the previous methods used do in fact recover preplay in the dataset (per those methods), for comparison.

4. The gain parameter γ in the emission model (Section 4.4.2, which scales place field firing rates for SWRs) is never specified. It is not listed among the optimized parameters {λ, σ, q}, and no fixed value is reported. The authors should state how γ is determined, report its value, and discuss sensitivity of results to this choice, since it directly affects decoder confidence and could bias regime classification.

5. When the authors first apply their model to real data (p. 9), almost no information is provided about the dataset. Key details (recording location, which behavioral epochs are included, number of neurons per session, SWR detection criteria) should be briefly stated at the point of first use, not only in the Methods.

6. The "no class" label appears in figures (Fig. 4, Fig. S6) but is never defined in the main text. It should be briefly explained when first referenced.

Typos and grammar:

- p. 12: "the existence stationary dynamics" → "the existence of stationary dynamics"

- p. 17: "cannot be interpreted as conclusively evidence" → "conclusive evidence"

- p. 17: "may contribute it" → "may contribute to it"

- p. 17: "may cause segments posterior to be consistent over space" — this sentence is garbled and the meaning is unclear

- p. 12: "Though this a conceptually provocative result" → "Though this is a..."

- p. 16: "neuron-identify shuffle" → "neuron-identity shuffle"

Reviewer #2: 2026_03 Review of Wu & Wei PLoS Comp Biol

The paper demonstrates a novel method for analyzing replay events in the hippocampus, which improves on prior methods by being more principled rather than ad hoc. The authors use the method to address some outstanding questions in the field of reactivation/replay. In this manner the paper helps advance the field.

I do have a few critiques, which I think the authors can address.

1) The principled system is set up as having three states, two of which (stationary and uniform) are highly constrained, the other of which (drift diffusion--DD) has more free parameters. Especially since a stationary state is a subset of drift diffusion models (just with drift and diffusion parameters of zero) it is important that the extra constraints (which reduce the likelihood of finding a transition to a state) are accounted for. Moreover, why not allow the DD state to transition to another DD state with any set of different parameters as opposed to only transition to a DD state with drift of zero (the stationary state)? Finally, on this topic, it would seem that to rule out the stationary state, once could simply provide the confidence interval of DD parameters and show that zero drift is not within the interval. The latter question also applies to the tests on whether data are fitted by a random walk, which is simply a DDM with drift of zero -- why not simply calculate the likelihood of parameters for the DDM and show that zero drift has much lower probability than the maximum likelihood drift?

2) Testing: the authors test spiking implementations of their HMM and assess how traditional models fail to capture some of its features. However, they do not do the converse: for example, traditional methods look at "maximum jump" in decoded location. It is one thing to find that a simulated DDM can appear to be decoded incorrectly as having a jump and to therefore say the new method is better, but that is somewhat tautological. One also needs to compare simulations where there are jumps in location and see how the HMM proposed decodes the results -- does it capture the "ground truth" when the "ground truth" follows the types of measures in previous decoding. Again, if the HMM allowed for a state transition to the *same type of state but with different parameters* I think it would be more general and capture an event such as stationary at one location followed by a jump to stationary at another location. With or without this decoding possibility, it is important to test how the new method deals with such situations (which in my view are quite likely/common in the data) as compared with the old methods that directly reveal such "jumps".

Other than these concerns, the paper is well-written and clear, and I have no concerns about methodology.

Reviewer #3: The manuscript by Wu and Wei outlines a novel approach to characterizing hippocampal replay. The existing literature consists of several different approaches that have led to confusion and differing points of view in the field. This study introduces a physics-inspired method to characterize candidate replays by drift and diffusion parameters and applies it to existing data to extract some important insights. I believe this represents an important contribution to the field based on the intuitive reasoning and meaningful results it provides. Nevertheless, there are a few areas in which the manuscript could be significantly improved.

Major

The study notes that “12.2% of events can be classified as drift-diffusion….” A key aspect that could affect the findings is how events are identified. In this study, ripple band power is used to identify candidate events, but this depends largely on electrode placement and ripple detection, which can be variable. Therefore many other studies rely on the detection of “population bursts” or “high-frequency events” using multi-unit activities that appear to be more reliable. Many replay studies also implement additional inclusion/exclusion criterion, such as ripple frequency power, or a threshold on the number of active cells. It’s unclear whether and how such choices could affect the results? I would recommend that the authors examine how their replay percentages and other results vary based on other criteria that are commonly used in the literature.

The study analyses so-called stationary events and compares these with shuffles. However, this is done without regards to the locations that are decoded during these events. In previous studies, stationary events were found to encode the animals current location, during immobility. It’s not clear whether this is also the case among shuffles in the current study. This may be an important distinction.It may also be that false-positive events (e.g. with spurious ripple power caused by chewing, etc.) are more likely to be stationary, which is another point that merits consideration. It would be helpful if the study provided example stationary events in the data as well as in the shuffles. In fact, the study should also provide more examples of other types of events, including uniform and “no class”.

The second event (top row) in Figure 6A (& Fig S2) raises some questions. To me, visually this does not appear to be a “drift” replay. I would guess it would qualify as a “fragmented” replay using the Denovellis criterion. The study should do more to convince the readers why we should trust this to be a true drift replay rather than a misclassification by the algorithm.

The study also makes it a point to compare results from a study based on foraging in a 2D environment with results from rewarded behavior on a 1D linear track. This could be a bit misleading because the animals’ experienced trajectories are considerably different in the two cases, as is the geometry of the environment, which makes these comparisons problematic. One option to remedy this situation is for the authors to apply their methods to behavior in a 2D environment. This should be doable given that the Pfeiffer and Foster dataset used in this study contains many such 2D sessions.

Finally, I found the treatment of the pre-run SWRs to be rather confusing, and the study appears to make contradictory conclusions. They appear to find that the data is above chance only if “chance” is assessed using one of the several shuffle possibilities, yet they conclude that their data supports “preplay”. But doesn’t this potentially have more to do with the suitability of different shuffles than it does of pre-existing structure, as they allude to in the discussion? It seems an equally valid conclusion could be that their analysis does not support the existence of pre-existing temporal structure, unless the study can provide arguments or evidence for trusting one surrogate dataset more than the others. Please clarify.

Minor

The claim of being “the most powerful way” in the Results section. needs further explicit support.

What is the basis for choosing a threshold of 0.7 for the drift rate? Is this arbitrary?

Please show some sample events for Fig S3B.

Extra comma in abstract “... that, only …”

“... or is even stationary?” doesn’t make sense.

Missing “of” in “the existence stationary dynamics”

**********

**Have the authors made all data and (if applicable) computational code underlying the findings in their manuscript fully available?**

The PLOS Data policy requires authors to make all data and code underlying the findings described in their manuscript fully available without restriction, with rare exception (please refer to the Data Availability Statement in the manuscript PDF file). The data and code should be provided as part of the manuscript or its supporting information, or deposited to a public repository. For example, in addition to summary statistics, the data points behind means, medians and variance measures should be available. If there are restrictions on publicly sharing data or code —e.g. participant privacy or use of data from a third party—those must be specified.

Reviewer #1: Yes

Reviewer #2: **No:**  All the analysis codes are available, but the paper includes analysis results based on experimental data previously published elsewhere that is not available -- the owners of the data are not authors of this paper.

Reviewer #3: Yes

**********

PLOS authors have the option to publish the peer review history of their article (what does this mean?). If published, this will include your full peer review and any attached files.

If you choose “no”, your identity will remain anonymous but your review may still be made public.

**Do you want your identity to be public for this peer review?**  For information about this choice, including consent withdrawal, please see our Privacy Policy.

Reviewer #1: No

Reviewer #2: No

Reviewer #3: No

[NOTE: If reviewer comments were submitted as an attachment file, they will be attached to this email and accessible via the submission site. Please log into your account, locate the manuscript record, and check for the action link "View Attachments". If this link does not appear, there are no attachment files.]

**Figure resubmission:**

**Reproducibility:**

To enhance the reproducibility of your results, we recommend that authors of applicable studies deposit laboratory protocols in protocols.io, where a protocol can be assigned its own identifier (DOI) such that it can be cited independently in the future. Additionally, PLOS ONE offers an option to publish peer-reviewed clinical study protocols. Read more information on sharing protocols at https://plos.org/protocols?utm_medium=editorial-email&utm_source=authorletters&utm_campaign=protocols

# Author comment: Author response to Decision Letter 1

**DOI:** 10.1371/journal.pcbi.1014761.r002

17 Jun 2026

Attachment Submitted filename: Wu_Wei_response to review.pdf

# Peer review: Decision Letter 1

**DOI:** 10.1371/journal.pcbi.1014761.r003

**Authors:** Thomas Serre, Matthias Helge Hennig

10 Jul 2026

PCOMPBIOL-D-26-00459R1

Drift-diffusion dynamics of hippocampal replay

PLOS Computational Biology

Dear Dr. Wei,

Thank you for submitting your manuscript to PLOS Computational Biology. As you can see, reviewer 3 still raises some minor points. Please consider these in a minor revision before we make a final decision.

Please submit your revised manuscript by Sep 09 2026 11:59PM. If you will need more time than this to complete your revisions, please reply to this message or contact the journal office at ploscompbiol@plos.org. When you're ready to submit your revision, log on to https://www.editorialmanager.com/pcompbiol/ and select the 'Submissions Needing Revision' folder to locate your manuscript file.

Please include the following items when submitting your revised manuscript:

* A letter that responds to each point raised by the editor and reviewer(s). You should upload this letter as a separate file labeled 'Response to Reviewers'. This file does not need to include responses to formatting updates and technical items listed in the 'Journal Requirements' section below.

* A marked-up copy of your manuscript that highlights changes made to the original version. You should upload this as a separate file labeled 'Revised Manuscript with Track Changes'.

* An unmarked version of your revised paper without tracked changes. You should upload this as a separate file labeled 'Manuscript'.

If you would like to make changes to your financial disclosure, competing interests statement, or data availability statement, please make these updates within the submission form at the time of resubmission. Guidelines for resubmitting your figure files are available below the reviewer comments at the end of this letter.

As the corresponding author, your ORCID iD is verified in the submission system and will appear in the published article. PLOS supports the use of ORCID, and we encourage all coauthors to register for an ORCID iD and use it as well. Please encourage your coauthors to verify their ORCID iD within the submission system before final acceptance, as unverified ORCID iDs will not appear in the published article. Only the individual author can complete the verification step; PLOS staff cannot verify ORCID iDs on behalf of authors.

We look forward to receiving your revised manuscript.

Kind regards,

Matthias Helge Hennig, Ph.D.

Section Editor

PLOS Computational Biology

Thomas Serre

Section Editor

PLOS Computational Biology

**Reviewers' comments:**

Reviewer's Responses to Questions

**Comments to the Authors:**

**Please note here if the review is uploaded as an attachment.**

Reviewer #1: The authors have addressed my concerns in their revised manuscript.

Reviewer #2: The authors have made a thorough set of responses to the original critiques.

Reviewer #3: The manuscript by Wu and Wei has been substantially improved. However, in several areas, my concerns were only partially addressed, and it seems some of my original comments may have been missed or misunderstood.

1. I previously recommended examining how the results would vary if standard detection criteria were used. Instead of doing this, the authors applied even more restrictive criteria to their event detection, which misses the point. As Reviewer 1 also noted, not all replay events occur during sharp-wave ripples (SWRs). The core concern is that the study needs to show how the results would look when using the standard methods applied in other papers, which typically use just MUA, without ripples in the LFP. This remains unaddressed.

2. The related overlap analysis was restricted only to the subset of events that were matched. This artificially inflates the median intersection-over-union (IoU) values because it completely ignores all the instances where IoU = 0 (no match), while still including instances where IoU = 1.

3. I previously requested more examples, including different types of real events as well as drift-diffusion fits to shuffle surrogates, but these were not provided. With only several hand-picked examples to go by (and the authors even removed one event that showed ambiguity), it is difficult to evaluate the true effectiveness of the technique.

4. The Methods section needs to explicitly describe the shuffles applied. For example, for the neuron-identity shuffle, it is unclear whether the shuffle was performed across all recorded neurons or just the subset active within a given event. I guess it is per-event based on Figures S5 and S6, but it is hard to be sure. Additionally, for Figure 5C, the text states that the place-field shift preserves field shape. I don't see how this is possible if a circular shift moves the field center to one of the edges—wouldn't that split the field into two halves on opposite ends?

5. It is currently unclear how stationary events and Brownian motion are expressed during pre-sleep. Figures 5 and 7 only look at awake and post-sleep periods; pre-sleep should be included for a complete picture.

6. Figure S7 is a nice addition because it examines mean-squared displacement (MSD) in stationary and uniform events. However, it is unclear why the "no-class" events were not also considered here.

Minor

1. Figure 2E doesn’t look right. Why is the posterior probability distribution whited out?

**********

**Have the authors made all data and (if applicable) computational code underlying the findings in their manuscript fully available?**

The PLOS Data policy requires authors to make all data and code underlying the findings described in their manuscript fully available without restriction, with rare exception (please refer to the Data Availability Statement in the manuscript PDF file). The data and code should be provided as part of the manuscript or its supporting information, or deposited to a public repository. For example, in addition to summary statistics, the data points behind means, medians and variance measures should be available. If there are restrictions on publicly sharing data or code —e.g. participant privacy or use of data from a third party—those must be specified.

Reviewer #1: Yes

Reviewer #2: Yes

Reviewer #3: Yes

**********

PLOS authors have the option to publish the peer review history of their article (what does this mean?). If published, this will include your full peer review and any attached files.

If you choose “no”, your identity will remain anonymous but your review may still be made public.

**Do you want your identity to be public for this peer review?**  For information about this choice, including consent withdrawal, please see our Privacy Policy.

Reviewer #1: No

Reviewer #2: No

Reviewer #3: No

[NOTE: If reviewer comments were submitted as an attachment file, they will be attached to this email and accessible via the submission site. Please log into your account, locate the manuscript record, and check for the action link "View Attachments". If this link does not appear, there are no attachment files.]

**Figure resubmission:**

**Reproducibility:**

To enhance the reproducibility of your results, we recommend that authors of applicable studies deposit laboratory protocols in protocols.io, where a protocol can be assigned its own identifier (DOI) such that it can be cited independently in the future. Additionally, PLOS ONE offers an option to publish peer-reviewed clinical study protocols. Read more information on sharing protocols at https://plos.org/protocols?utm_medium=editorial-email&utm_source=authorletters&utm_campaign=protocols

# Author comment: Author response to Decision Letter 2

**DOI:** 10.1371/journal.pcbi.1014761.r004

18 Aug 2026

Attachment Submitted filename: Response to review_minor revision.pdf

# Editor report: Decision Letter 2

**DOI:** 10.1371/journal.pcbi.1014761.r005

**Authors:** Matthias Hennig

26 Aug 2026

Dear Dr. Wei,

We are pleased to inform you that your manuscript 'Drift-diffusion dynamics of hippocampal replay' has been provisionally accepted for publication in PLOS Computational Biology.

Before your manuscript can be formally accepted you will need to complete some formatting changes, which you will receive in a follow up email. A member of our team will be in touch with a set of requests.

Please note that your manuscript will not be scheduled for publication until you have made the required changes, so a swift response is appreciated.

IMPORTANT: The editorial review process is now complete. PLOS will only permit corrections to spelling, formatting or significant scientific errors from this point onwards. Requests for major changes, or any which affect the scientific understanding of your work, will cause delays to the publication date of your manuscript.

Should you, your institution's press office or the journal office choose to press release your paper, you will automatically be opted out of early publication. We ask that you notify us now if you or your institution is planning to press release the article. All press must be co-ordinated with PLOS.

Thank you again for supporting Open Access publishing; we are looking forward to publishing your work in PLOS Computational Biology.

Best regards,

Matthias Helge Hennig, Ph.D.

Section Editor

PLOS Computational Biology

Thomas Serre

Section Editor

PLOS Computational Biology

***********************************************************

# Editor report: Acceptance letter

**DOI:** 10.1371/journal.pcbi.1014761.r006

**Authors:** Matthias Hennig

PCOMPBIOL-D-26-00459R2

Drift-diffusion dynamics of hippocampal replay

Dear Dr Wei,

I am pleased to inform you that your manuscript has been formally accepted for publication in PLOS Computational Biology. Your manuscript is now with our production department and you will be notified of the publication date in due course.

The corresponding author will soon be receiving a typeset proof for review, to ensure errors have not been introduced during production. Please review the PDF proof of your manuscript carefully, as this is the last chance to correct any errors. Please note that major changes, or those which affect the scientific understanding of the work, will likely cause delays to the publication date of your manuscript.

Soon after your final files are uploaded, unless you have opted out, the early version of your manuscript will be published online. The date of the early version will be your article's publication date. The final article will be published to the same URL, and all versions of the paper will be accessible to readers.

For Research, Software, and Methods articles, you will receive an invoice from PLOS for your publication fee after your manuscript has reached the completed accept phase. If you receive an email requesting payment before acceptance or for any other service, this may be a phishing scheme. Learn how to identify phishing emails and protect your accounts at https://explore.plos.org/phishing.

Thank you again for supporting PLOS Computational Biology and open-access publishing. We are looking forward to publishing your work!

With kind regards,

Sharmila Kamatchi

PLOS Computational Biology | Carlyle House, Carlyle Road, Cambridge CB4 3DN | United Kingdom ploscompbiol@plos.org | Phone +44 (0) 1223-442824 | ploscompbiol.org | @PLOSCompBiol
